# Estimation of the mass density of biological matter from refractive index measurements

**DOI:** 10.1016/j.bpr.2024.100156

**Published:** 2024-04-24

**Authors:** Conrad Möckel, Timon Beck, Sara Kaliman, Shada Abuhattum, Kyoohyun Kim, Julia Kolb, Daniel Wehner, Vasily Zaburdaev, Jochen Guck

**Affiliations:** 1 Max Planck Institute for the Science of Light, Erlangen, Germany; 2 Max-Planck-Zentrum für Physik und Medizin, Erlangen, Germany; 3 Friedrich-Alexander-Universität Erlangen-Nürnberg, Erlangen, Germany

## Abstract

The quantification of physical properties of biological matter gives rise to novel ways of understanding functional mechanisms. One of the basic biophysical properties is the mass density (MD). It affects the dynamics in sub-cellular compartments and plays a major role in defining the opto-acoustical properties of cells and tissues. As such, the MD can be connected to the refractive index (RI) via the well known Lorentz-Lorenz relation, which takes into account the polarizability of matter. However, computing the MD based on RI measurements poses a challenge, as it requires detailed knowledge of the biochemical composition of the sample. Here we propose a methodology on how to account for assumptions about the biochemical composition of the sample and respective RI measurements. To this aim, we employ the Biot mixing rule of RIs alongside the assumption of volume additivity to find an approximate relation of MD and RI. We use Monte-Carlo simulations and Gaussian propagation of uncertainty to obtain approximate analytical solutions for the respective uncertainties of MD and RI. We validate this approach by applying it to a set of well-characterized complex mixtures given by bovine milk and intralipid emulsion and employ it to estimate the MD of living zebrafish (*Danio rerio*) larvae trunk tissue. Our results illustrate the importance of implementing this methodology not only for MD estimations but for many other related biophysical problems, such as mechanical measurements using Brillouin microscopy and transient optical coherence elastography.

## Why it matters

Mass density is a fundamental property of living matter. It is of central importance for understanding inherent functional mechanisms by quantifying (statistical) processes and associated mechanical properties. Currently, there is no experimental paradigm available to measure in vivo mass density directly. Indirectly, the mass density can be inferred from refractive index measurements. However, we lack a robust framework that is capable of accounting for the complex chemical composition of biological matter. Our manuscript directly addresses this gap by establishing an experimentally validated, cohesive theoretical framework on how mass density of biological matter can be estimated using (in vivo) refractive index measurements.

## Introduction

Quantifying the physical properties of biological matter has become increasingly important over recent decades. The notion that biological function of cells and tissues is affected by their physical phenotype and vice versa has been validated in many experimental studies (see, e.g., (1,2,3)). A fundamental property of matter, including living matter, is the mass density (MD) (4), which not only is associated with buoyancy, crowdedness (5), biomolecular condensation (6), and inherent dynamical processes of the sample of interest (7,8) but also plays a major role in elastography, particularly Brillouin microscopy (9,10,11,12) and transient optical coherence elastography (see, e.g., (13,14)). However, measuring the in vivo MD distribution in a direct manner poses a challenge that has not been resolved so far. One way of inferring the in vivo MD of a sample is to measure the refractive index (RI) via microscopy techniques, such as quantitative phase imaging (QPI), particularly optical diffraction tomography (ODT) (15,16). The Lorentz-Lorenz relation then connects the RI with the MD if the molar refractivity and partial specific volume (PSV) of the dry mass composition and the solvent content are known. This knowledge, however, is not trivially obtainable. A customary assumption regarding biological matter is that the dry mass composition is given by proteins only (16,17,18,19) and that the solvent content is then indirectly constrained by the measured RI. Although this approximation holds for binary solutions, it cannot be directly extended to samples with a complex dry mass composition.

In the context of cells and tissues, the dry mass composition may be thought of as a mixture of (phase-separated) proteins, lipids, sugars, etc. (20). By employing, e.g., mass spectrometry (MS) and/or (stimulated) Raman spectroscopy ((S)RS), individual components and their respective concentrations in the sample can be identified (21,22,23). Additionally, correlative fluorescence information could be employed to segment RI maps acquired by ODT (12,24). However, these experimental modalities might not be available or applicable for certain samples, which creates a degree of ignorance about the dry mass composition that should be considered in the inference process of obtaining an MD estimate. Another closely related aspect is the robust estimation of the uncertainty of the MD. Considering the previously mentioned degree of ignorance, these uncertainties are clearly not only of statistical but also of a systematic nature. Further, even if universal knowledge about the true distributions of the molar refractivity and PSV were available, to estimate the uncertainty of the MD adequately, the uncertainties of the individual parameters should be propagated.

Here, we present a robust methodology for estimating the uncertainties of the MD and the correlative RI by employing Monte-Carlo (MC) simulations. We provide analytical approximations for both the MD and RI distributions in dependence of the dry mass composition and the solvent content, employing Gaussian propagation of uncertainty.

To this end, we first motivate a simple mixture model to estimate the relationship between the MD and the RI from two material constants, namely the RI increment *α* and the PSV *θ*. We then extend the model toward unimodal distributions of *α* and *θ*, for which previously only precise values were assumed.

The distributions of the RI increment and PSV are remarkably narrow when only considering proteins in the mixture (15,25), resulting in sharp distributions of RI and MD. However, taking a second type of molecule, such as lipids or sugars, into account adds an additional complexity to the MD estimations since their values of RI increment and PSV differ drastically from those of proteins. Therefore, we derive an effective description of the system based on weighted mixture distributions. This allows for a correlative prediction of the RI and the MD, accounting, e.g., for the lipid and water content of the sample and fluctuations in both quantities. We then apply this approach to 1) bovine milk, a well-characterized mixture of water, proteins, and lipids; and 2) 20% intralipid emulsion (IL), which mainly consists of water and soybean oil. Comparing the measured values of the MD and RI acquired by pycnometry and Abbe refractometry, respectively, with our theoretical estimates, we find both to be in good agreement with each other.

After demonstrating the applicability of our model on bovine milk and IL, we explore the MD distribution of larval zebrafish trunk (comprising major tissue, including muscle and spinal cord) employing the recent RI and MS measurements of (21). Although the accurate determination of the biochemical composition of the tissue poses a challenge, beset by numerous (crude) uncertainties, the measured RI distribution and our prediction coincide within one standard deviation (SD). This purely optical and computational approach shows how the MD can be estimated in complex in vivo specimens, enabling a more profound interpretation of mechanical measurements.

## Materials and methods

### Sample preparation

The skim-milk powder (SM) was dissolved in distilled water while carefully stirring the solution to avoid foaming. The solution was then left on a tilt/roller mixer for approximately 30 min. For the IL, according amounts of water were added to the emulsion and the solution was left on a tilt/roller mixer for approximately 30 min as well. All samples were freshly prepared before the measurements were performed.

### Abbe refractometry and pycnometry

For measuring the RI of a liquid sample, 100 *μ*L of the sample were loaded into an Abbe refractometer (KERN ORT 1RS) and a commercially available flashlight light-emitting diode (LED) was employed as illumination source.

To determine the density of a liquid sample, a pycnometer (Blaubrand 43305) was employed. First, the volume of the pycnometer was determined by employing distilled water as a calibration sample (N=10 technical repetitions) as

(1) $$v_{\text{pyc}}=\frac{m_{w}-m_{\text{pyc}}}{\rho_{w,\text{lit}.}}=\left(4.9455\pm 0.0017\right)\;\text{mL,}$$

where $m_{w}$ is the mass of the pycnometer filled with water, $m_{\text{pyc}}$ is the mass of the empty pycnometer, and $\rho_{w,\text{lit}.}=0.997\;g/\text{mL}$ is the literature value of the density of water at 23°C. The respective masses were measured using a high-precision lab scale (Ohaus Pioneer PX124). The density of the liquid sample under study was then computed by

(2) $$\rho =\frac{m-m_{\text{pyc}}}{v_{\text{pyc}}}\text{.}$$

### Measurement uncertainties

The systematic uncertainties of the respective measurement devices under use were taken from the manuals and considered in all calculations together with the statistical uncertainties as

(3) $$\Delta z=\sqrt{\Delta z_{\text{sys}}^{2}+\Delta z_{\text{stat}}^{2}}\text{,}$$

where *z* is an arbitrary observable.

### Data analysis

All data analysis, plotting, and simulations were performed using custom scripts in Wolfram Research, Mathematica, Version 12.2 (26).

## Background

### A binary mixture model for MD estimations

In the following, we consider a mixture of a solute (denoted by index 2) in a solvent (denoted by index 1) with total mass *m* and volume *v*. The MD ρ=m/v of the mixture can be expressed in terms of the MDs $\rho_{i}$ and volume fractions $\varphi_{i}=c_{i}/\rho_{i}$ of its components as

(4) $$\rho =\varphi_{1}\rho_{1}+\varphi_{2}\rho_{2}\text{,}$$

where the $c_{i}=m_{i}/v$ are the concentrations of the respective constituents and we denote the total volume of the mixture by $v=v_{1}+m_{2}\vartheta$. Here, *ϑ* is the so-called apparent specific volume (ASV) of the solute, which describes the volume per gram of the solute in solution. As such, the ASV may be dependent on the mass of the solute $m_{2}$, since it accounts for solute-solute interactions under constant temperature *T*, pressure *p*, and solvent mass $m_{1}$. The change of the total volume of the solution *v* with respect to a change in the mass of the solute is then characterized by the PSV via

(5) $$\theta \equiv {\left(\frac{\partial v}{\partial m_{2}}\right)}_{T,p,m_{1}}=\vartheta +m_{2}{\left(\frac{\partial \vartheta }{\partial m_{2}}\right)}_{T,p,m_{1}}\text{,}$$

as motivated in (27). For the sake of simplicity, for all the following considerations, we employ the concept of volume additivity, for which it is straightforward to show that $\theta =\vartheta =1/\rho_{2}$ (see Eq. S3 in the Supporting Material).

By assuming volume additivity $\left[\left(1-\varphi_{2}\right)=\varphi_{1}\right]$, we may express Eq. 4 in terms of the solvent MD, PSV, and solute concentration as

(6) $$\rho =\rho_{1}\left(1-c_{2}\theta \right)+c_{2}\text{.}$$

In the next step, we connect the RI of the solution to the solute concentration $c_{2}$. For that purpose we employ the phenomenological mixing rule

(7) $$n=\varphi_{1}n_{1}+\varphi_{2}n_{2}\text{,}$$

where we assumed that the RI follows the same mixture rule as the MD, given in Eq. (4). In the following, we refer to Eq. 7 as Biot equation or mixing rule (28), which is commonly employed in the context of QPI and ODT (15). Note that, in a typical ODT experiment, we determine the RI contrast $\delta n\equiv n-n_{1}$, which has to fulfill a nonnegativity constraint (i.e., δn≥0). Evaluating Eq. 7 while assuming volume additivity, we arrive at

(8) $$\frac{\delta n}{c_{2}}=\theta \left(n_{2}-n_{1}\right)=\alpha \text{,}$$

where we identify the customarily designated RI increment $\alpha \equiv \partial n/\partial c_{2}$.

Finally, by replacing $c_{2}$ in Eq. 6 with the expression given in Eq. 8, we obtain an estimate of the solution MD in dependence of the RI as

(9) $$\rho =\frac{\delta n}{\alpha }+\rho_{1}\left(1-\theta \frac{\delta n}{\alpha }\right)\text{,}$$

which has been employed in (19), or stated equivalently in (29). Eq. 9 is the basis of all the following considerations and will be employed frequently throughout this study. We note that the relationship between MD and RI, given in Eq. 9 is linear and the slope $\partial \rho /\partial n=\left(1-\rho_{1}\theta \right)/\alpha$ is only determined by the PSV *θ* and the RI increment *α* of the solute. In biological matter, the solvent is assumed to be water, for which the MD $\rho_{1}$ and RI $n_{1}$ are accurately known.

Experimentally, the PSV of a solute is determined by measuring the density of the solution *ρ* in dependence of the solute concentration $c_{2}$ (27,30). A straightforward and common approach to obtain the MD of a liquid is given by pycnometry, where the mass of a precisely fixed volume is measured. The MD is then simply given by the mass-to-volume ratio. Further methods to measure MD are reviewed in (27) and references therein. Similarly, the RI increment is customarily determined by measuring the RI of the solution in dependence of the solute concentration (see Eq. 8), e.g., by employing an Abbe refractometer.

To our knowledge, the justification for or against the assumption of volume additivity in complex biological matter is yet to be given (experimentally). We may interpret Eq. 5 in first-order approximation as θ≈ϑ±Δϑ, where any potential deviation from the volume additivity Δϑ could be treated as an additional factor that will also contribute to the uncertainty of the RI and MD estimates.

**Figure 1:**
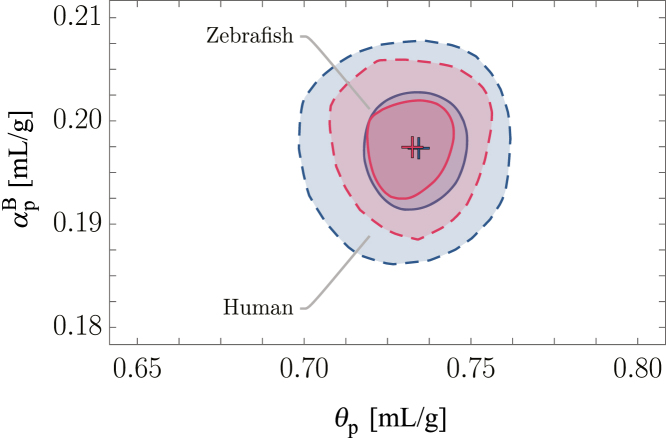
Correlative distributions $P\left(\alpha_{p}^{B},\theta_{p}\right)$ of the RI increment $\alpha_{p}^{B}$ and PSV $\theta_{p}$ for the human proteome (*blue*; 82,127 proteins included) and the zebrafish proteome (*red*; 46,517 proteins included). The solid and dashed lines indicate the 68% and 95% confidence contours, respectively.

As pointed out earlier, Eq. 8 is commonly used in the context of QPI and ODT to determine solute densities ($\rho_{2}=1/\theta$) and subsequently solute masses of complex biological samples from tomographic RI measurements (15). To that aim the RI increments of various proteins are customarily employed (17, 18, 31,32,33,34,35). Although this approach holds for the assumption that biological matter consists of protein and water, we will show later that it is not sufficient for inferences about complex solute composition with more than one constituent. In brief, this is because there exists no universal relationship α∼θ (i.e., n∼ρ) for different materials (see, e.g., (36); Fig. 1).

### Complex mixtures

In the context of biological matter, one may think of the solute as a complex composition of many constituents, which makes the experimental determination of both PSV and RI increment for all components and their combinations practically impossible; the human proteome alone consists of $∼10^{4}$ proteins (20,37). To resolve this problem, at least partially, without accounting for volume inadditivities, a method of determining the correlative distributions P(α,θ) of the proteomes of different organisms was introduced and experimentally validated for two amino acid sequences in (25). The authors computed the molar mass averages of the residue refractivity per gram and according PSV *θ* of proteins based on their respective amino acid sequence. The refractivity per gram $R_{i}$ of a molecule *i* is proportional to its polarizability ${\hat{\alpha }}_{i}$ and molar mass $M_{i}$ as $R_{i}∼{\hat{\alpha }}_{i}/M_{i}$. The refractivity per gram can then be connected to the RI and the MD of the solute via the Lorentz-Lorenz (or Clausius-Mossotti) relation (see, e.g., (38)) as

(10) $$R_{i}=\frac{1}{\rho_{i}}\frac{n_{i}^{2}-1}{n_{i}^{2}+2}\text{.}$$

We note that taking the mass averages over the amino acid residue refractivities per gram in combination with the Lorentz-Lorenz relation Eq. 10 leads to the Lorentz-Lorenz mixing rule of RIs Eq. S8.

However, assuming dilute solutions ($n\to n_{1}$) and volume additivity, the authors of (25) computed the RI increment for each protein (denoted by the index p) by employing the Wiener mixing rule of RIs (see, e.g., (28)) and Eq. 10 to compute the RI of each protein $n_{p}$ from the mass averaged refractions per gram and PSVs of the respective amino acid sequences, as described earlier. Repeating this procedure for all proteins presumed to be abundant in the different organisms under study, they obtained the bivariate distribution of RI increment and PSV. Zhao et al. (25) then fitted normal distributions to the univariate histograms to obtain the means and SDs for different organisms, of which we depict two in Table 1. We repeated the computations presented in (25) (see Supporting Material) for the updated human and zebrafish proteomes obtained from (37). Besides computing the RI increment from the Wiener relation for dilute solutions, we also employed the Biot Eq. 7 in combination with the volume additivity assumption to obtain an expression of the RI increment as given in Eq. 8.

**Table 1:** Mean and SD of the RI increments $\alpha_{p}^{i}$ and PSVs $\theta_{p}$ Distributions Based on Amino Acid Sequences of the Proteome of the Human and Zebrafish, and the Trunk Tissue of the Larval Zebrafish at 96 hpf

	${\bar{\alpha }}_{p}^{W}$ in mL/g	$\Delta \alpha_{p}^{W}$ in mL/g	${\bar{\alpha }}_{p}^{B}$ in mL/g	$\Delta \alpha_{p}^{B}$ in mL/g	${\bar{\theta }}_{p}$ in mL/g	$\Delta \theta_{p}$ in mL/g
Human						
Proteome^a^	0.1899	0.0030	N/A	N/A	0.735	0.010
Proteome^b^	0.188	0.004	0.197	0.004	0.734	0.012
Zebrafish						
Proteome^a^	0.1904	0.0030	N/A	N/A	0.735	0.010
Proteome^b^	0.1887	0.0031	0.1974	0.0034	0.732	0.010
Trunk tissue^b^	0.1883	0.0029	0.1971	0.0033	0.734	0.009

The calculation of $\alpha_{p}^{B}$ employed the Biot equation given in Eq. 7, whereas $\alpha_{p}^{W}$ was derived from the dilute limit of the Wiener mixing rule of refractive indices (28).

a Zhao et al. (25)

b This work

Additionally, different from (25), we employed the consensus averages for the amino acid residue molecular volumes of (30) instead of the ones of Cohn and Edsall (39). The full list of parameters employed here is given in Table S1. The resulting bivariate distributions P(α,θ) are given in Fig. 1. The corresponding mean values and SDs of the fits of the univariate histograms with a normal distribution are given in Table 1. Evidently, the PSV and RI increment values obtained here, using the Wiener equation for dilute solutions, coincide well with the values from (25). We note that the RI increments obtained using Eq. 8 are systematically higher than the ones obtained from the Wiener equation for dilute solutions.

Although it is not clear why the calculations proposed in Zhao et al. (25) result in distributions P(α,θ) that resemble uncorrelated bivariate normal distributions for the proteomes of different species, they facilitate the idea of taking the whole distribution P(α,θ) into account when estimating the MD via Eq. 9. Consistently, to obtain a reliable estimate of the MD distribution, a precise characterization of the solute composition is needed.

We note that, although relations similar to Eq. 9 can be found for different (phenomenological) RI mixing rules (see, e.g., (40,41,42)), or effective (nonlocal) RI descriptions based on light scattering theory (see, e.g., (32,43,44,45,46)), we expect limited additional qualitative insight compared to the Biot Eq. 7; ultimately, for each theory one may compute the RI increment $\alpha \left(c_{2}\right)$, (numerically) solve the expression for the solute concentration $c_{2}$, and substitute it in Eq. 6 to find a relationship ρ(δn) for the given RI mixture rule. To illustrate this procedure, we derive such an expression for the Lorentz-Lorenz mixing rule in the Supporting Material (see Eq. S11).

In the following, we construct and employ a theoretical framework in which the information of the solute composition is incorporated into the prediction of the MD. For that purpose, we employ the Biot Eq. 7 for the majority of our further considerations and denote $\alpha =\alpha^{B}$.

## Results

### Extension of the binary mixture model

When dealing with biological matter, the complexity of the solute composition should be taken into account to obtain reliable estimates of the MD. To that aim, we first extend Eq. 9 to the case of different solute constituents (e.g., proteins of the human proteome, lipids, and sugars) being dissolved in a solvent with corresponding RI $n_{1}$ and MD $\rho_{1}$.

We describe this problem by discretizing the total sample volume into $N_{v}$ voxels with volumes $v_{v}$

(11) $$v=N_{v}v_{v}\text{.}$$

Furthermore, we discretize the voxels into $N_{0}$ “voxelinos” with volumes $v_{0}$ as

(12) $$v_{v}=N_{0}v_{0}\text{.}$$

**Figure 2:**
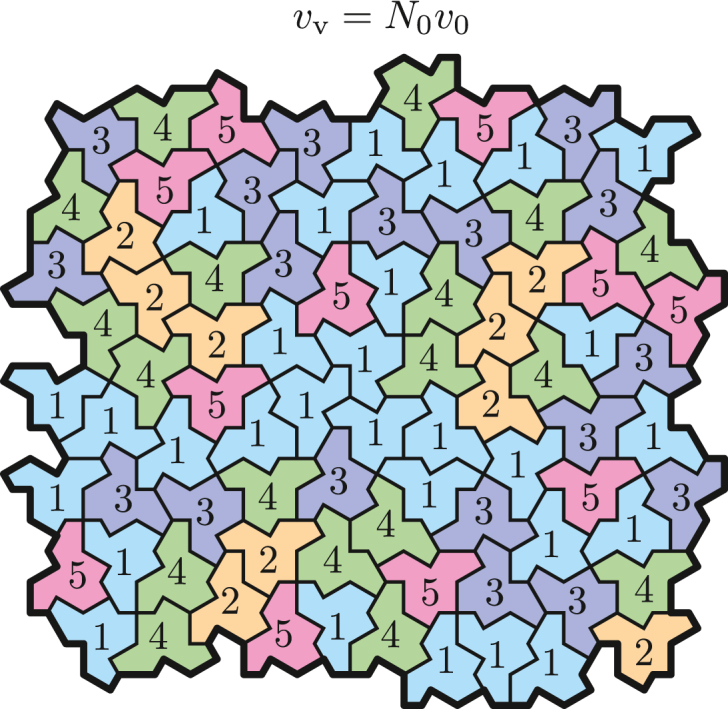
2D visual interpretation of a voxel with volume $v_{v}=N_{0}v_{0}$ consisting of $N_{0}$ voxelinos with volumes $v_{0}$. Each number stands for one type of voxelino; 1 corresponds to the solvent voxelinos and 2−5 correspond to the solute voxelinos, e.g., four different proteins. Each voxelino is characterized by its PSV $\theta_{i}$, refraction per gram $R_{i}$ and has a corresponding mass of $m_{i}=v_{0}/\theta_{i}$. This illustrative depiction is based on (47).

The motivation to divide a voxel into $N_{0}$ voxelinos is to obtain small, yet macroscopic, volume fractions with a constant volume $v_{0}$ that contain one and only one solution constituent. Hence, each voxelino can be either a solvent or solute voxelino and is inherently characterized by its respective PSV $\theta_{i}$ and refraction per gram $R_{i}$ (i.e., its MD and RI). The number of solvent voxelinos in the voxel, $N_{1}$, is given by $N_{1}=N_{0}-N_{s}$, where $N_{s}$ is the number of solute voxelinos (see Fig. 2 for a visual interpretation).

Accordingly, the solvent volume fraction of a voxel is given by

(13) $$\varphi_{1}\equiv 1-\frac{N_{s}}{N_{0}}\text{.}$$

Accounting for multiple types of solute molecules (e.g., proteins, lipids, sugars) being present in the solution, we chose the PSVs and refractions per gram of the solute voxelinos $\theta_{i+1}$ and $R_{i+1}$ to be random values, drawn from a weighted mixture distribution

(14) $$P_{\text{mix}}\left(R,\theta \right)=\sum_{j}x_{j}P_{j}\left(R,\theta \right)\text{,}$$

where *j* denotes the solute constituents. Further, the $P_{j}\left(R,\theta \right)$ represent the bivariate probability distributions of the refraction per gram and PSV of the respective constituents, and the associated weights $x_{j}$ are given by the relative volume fractions as $x_{j}\equiv N_{j}/N_{s}$. We note that Eq. 14 should be seen as a way of denoting that $N_{j}$ out of $N_{s}$ solute voxelinos of constituent *j* are present in a voxel. Consequently, $N_{j}$ solute voxelinos have the RI $n_{j}$ and MD $\rho_{j}$ and we have that $N_{s}=\sum_{j}N_{j}$, i.e., $\sum_{j}x_{j}=1$.

Employing Eqs. 6 and 7, we readily obtain the relation between the MD *ρ* and the RI *n* of one voxel

(15) $$\rho =\sum_{i=1}^{N_{s}}c_{i+1}+\rho_{1}\left(1-\sum_{i=1}^{N_{s}}\theta_{i+1}c_{i+1}\right)\equiv \frac{\delta n}{\alpha_{\text{eff}}}+\rho_{1}\left(1-\theta_{\text{eff}}\frac{\delta n}{\alpha_{\text{eff}}}\right)\text{,}$$

where we defined the effective RI increment $\alpha_{\text{eff}}$ and PSV $\theta_{\text{eff}}$. The RI of the solution *n* is then given by the Biot Eq. 7, where the RIs of the individual solute voxelinos $n_{i+1}$ could be known directly from RI measurements, or may be computed by employing the Lorentz-Lorenz relation, given in Eq. 10.

It can be shown (see Eqs. S17 and S18) that the effective parameters may be expressed by the mass averages of the respective parameters of the solute voxelinos as

(16) $$\begin{array}{l} \theta_{\text{eff}}=\sum_{i=1}^{N_{s}}y_{i+1}\theta_{i+1}\text{,}\\ \alpha_{\text{eff}}=\sum_{i=1}^{N_{s}}y_{i+1}\alpha_{i+1}\text{,} \end{array}$$

where we denoted the relative mass fraction of a solute voxelino by $y_{i}\equiv m_{i}/m_{s}\text{,}$ with $m_{s}$ being the total solute mass. Hence, the effective parameters absorb the complexity of the mixture, whereas the functional relationship of Eq. 6 is obeyed. With this at our disposal, we are able to compute the MD *ρ* and the corresponding RI *n* of a voxel, given a distribution $P_{\text{mix}}\left(R,\theta \right)$ and a solvent volume fraction of the voxels $\varphi_{1}$ (i.e., the ratio $N_{s}/N_{0}$). Repeating this procedure for $N_{v}$ voxels provides a map of RI values in resemblance of an ODT measurement alongside the corresponding MDs.

Since the framework introduced above is strongly dependent on a priori assumptions of the individual model parameters ($\varphi_{1}$, $x_{j}$, $\alpha_{j}$, $\theta_{j}$) for different complex mixtures, in the next step, we discuss the impact of these assumptions on the uncertainties of the RI and MD predictions.

### Uncertainty quantification of the extended model

Given access to experimental data (i.e., ODT tomograms of a sample of interest), we may compare not only the mean values of the measured and predicted RI distributions but also their widths. This in turn enables a more reliable estimate of the MD distribution. Hence, in the next step, we study the dependence of the uncertainty of the MD estimate *ρ*, defined in Eq. 15, and the RI *n* on the sample properties, namely, the solvent volume fraction $\varphi_{1}$, the effective RI increment $\alpha_{\text{eff}}$, the effective PSV $\theta_{\text{eff}}$, and their respective associated uncertainties.

To that aim, we investigated the impact of the presence of a second type of macro molecule in the mixture of proteins and water. Since lipids make up for about 13% of the solute mass fraction in mammalian cells (20) and typically exhibit an MD lower than water, they merit detailed scrutiny. In the following, we assume that the lipids are present in the form of lipid droplets and form an emulsion in the water + protein phase. For the sake of simplicity, we further assume that the lipid droplets consist only of the neutral lipid triolein, neglecting sterol esters, triacylglycerols, and phospholipids (48,49,50). The corresponding values of the RI, refraction per gram, PSV, and RI increment of the two types of solute molecules under investigation are given in Table S2.

Throughout this study, we assume that both RI and MD of the solvent are precisely known. We further point out that we implement the values given in Table S2 in our calculations as follows: if a quantity is stated as mean ± SD, we account for it as normally distributed with respective mean and SD. For the cases where we could not estimate an uncertainty, we assume the quantity to be delta distributed.

#### Effective RI increment and PSV

Examining the definitions of $\alpha_{\text{eff}}$ and $\theta_{\text{eff}}$, given in Eq. 16, we observe a dependence of both quantities on the number of solute voxelinos per voxel $N_{s}$, given as the upper limit of the sum. To obtain an intuition about the implications on the respective uncertainties, we first consider the case of proteins dissolved in water, which may be approximated by uncorrelated normal distributions N(μ,σ) of the RI increment and PSV, as shown in Fig. 1

(17) $$\begin{array}{l} \alpha^{N}∼N\left(\bar{\alpha },\Delta \alpha \right)\text{,}\\ \theta^{N}∼N\left(\bar{\theta },\Delta \theta \right)\text{,} \end{array}$$

with respective mean values and SDs (see, e.g., Table 1). Applying Gaussian propagation of uncertainty to Eq. 16, we have

(18) $$\begin{array}{l} \Delta \theta_{\text{eff}}=\frac{\Delta \theta }{\sqrt{N_{s}}}\text{,}\\ \Delta \alpha_{\text{eff}}=\frac{\Delta \alpha }{\sqrt{N_{s}}}\text{,} \end{array}$$

in which the relative mass fraction of each solute voxelino is given by $y_{i+1}=1/N_{s}$. The result of Eq. 18 is in concordance with the central limit theorem. In other words, the SD of the effective distributions corresponds to the SE of the mean of the initial distributions, given that the voxel contains $N_{s}$ protein voxelinos. Thus, considering that, in biological matter, the number of voxelinos per voxel would be typically larger than $∼10^{8}$ for experimentally accessible voxel sizes in the order of 1 ${\mathrm{\mu m}}^{3}$, the deviations of the effective RI increment and PSV for the case of proteins dissolved in water are negligibly small.

Next, we study the impact of lipids in the protein + water mixture. In this scenario, Eq. 14 takes the form

(19) $$P_{p+\text{lip}}\left(R,\theta \right)=\left(1-x_{\text{lip}}\right)P_{p}\left(R,\theta \right)+x_{\text{lip}}P_{\text{lip}}\left(R,\theta \right)\text{.}$$

Assuming that $P_{p}$ and $P_{\text{lip}}$ follow uncorrelated bivariate normal distributions, using Eq. 18, we find that the distributions of the effective PSV and RI increment follow normal distributions as

(20) $$\begin{array}{l} \theta_{\text{eff}}∼N\left({\bar{\theta }}_{\text{eff}},\Delta \theta_{\text{eff}}^{0}/\sqrt{N_{s}}\right)\text{,}\\ \alpha_{\text{eff}}∼N\left({\bar{\alpha }}_{\text{eff}},\Delta \alpha_{\text{eff}}^{0}/\sqrt{N_{s}}\right)\text{,} \end{array}$$

where the respective mean values of effective PSV and RI increment are given in Eq. 16 and the SDs $\Delta \theta_{\text{eff}}^{0}$, $\Delta \alpha_{\text{eff}}^{0}$ follow the SD of a mixture distribution (see Eq. S14).

With this, we further examine the implications of deviations of the relative lipid volume fraction $\Delta x_{\text{lip}}$ from voxel to voxel. Such deviations may be interpreted as a form of inhomogeneity of the system, which have been experimentally quantified in cells and tissues by stimulated Raman spectroscopy (SRS) measurements (22). Employing Gaussian propagation of uncertainty, we find the following analytical expression of the deviation of the mean effective PSV and RI increment with respect to $\Delta x_{\text{lip}}$ as

(21) $$\begin{array}{l} \Delta \theta_{\text{eff}}={\left[{\left(\frac{\Delta \theta_{\text{eff}}^{0}}{\sqrt{N_{s}}}\right)}^{2}+{\left(\frac{\partial {\bar{\theta }}_{\text{eff}}}{\partial {\bar{x}}_{\text{lip}}}\Delta x_{\text{lip}}\right)}^{2}\right]}^{1/2}\text{,}\\ \Delta \alpha_{\text{eff}}={\left[{\left(\frac{\Delta \alpha_{\text{eff}}^{0}}{\sqrt{N_{s}}}\right)}^{2}+{\left(\frac{\partial {\bar{\alpha }}_{\text{eff}}}{\partial {\bar{x}}_{\text{lip}}}\Delta x_{\text{lip}}\right)}^{2}\right]}^{1/2}\text{.} \end{array}$$

**Figure 3:**
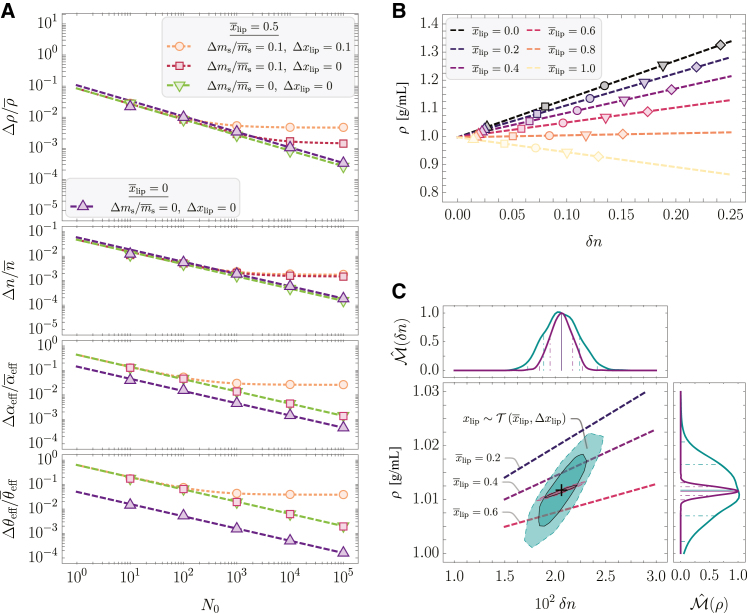
Results of MC simulations and according analytical solutions for the mixture of human proteins and the neutral lipid triolein in water. (*A*) Relative deviations of the MD *ρ*, the RI *n*, the effective RI increment $\alpha_{\text{eff}}$, and PSV $\theta_{\text{eff}}$ in dependence of the number of voxelinos per voxel $N_{0}$ obtained from MC simulations (*symbols*) and analytical solutions (*dashed lines*) for different mean relative lipid volume fractions ${\bar{x}}_{\text{lip}}$, associated deviations $\Delta x_{\text{lip}}$, and relative deviations of the number of the solute mass $\Delta m_{s}/{\bar{m}}_{s}$. The MC simulations were performed for a mean water volume fraction of ${\bar{\varphi }}_{1}=0.9$ and $N_{v}=10^{3}$. (*B*) MD *ρ* in dependence of the RI contrast δn for different mean relative lipid volume fractions ${\bar{x}}_{\text{lip}}$ and mean water volume fractions (${\bar{\varphi }}_{1}=0.1$, ${\bar{\varphi }}_{1}=0.3$, ${\bar{\varphi }}_{1}=0.5$,  ${\bar{\varphi }}_{1}=0.7$, ${\bar{\varphi }}_{1}=0.9$) for $\Delta x_{\text{lip}}=0$, $N_{0}=10^{3}$ and $N_{v}=10^{2}$. The dashed lines indicate the analytical solutions of Eq. 15. (*C*) Correlative distribution of the MD *ρ* and the RI contrast δn (the solid and dashed lines indicate the 68% and 95% confidence contours) with the corresponding normalized marginal probability density distributions $\hat{M}\left(\rho \right)$ and $\hat{M}\left(\delta n\right)$, respectively (the solid line represents the median, the dash-dotted and dashed lines indicate the 68% and 95% CIs), for the exact relative lipid volume fraction ${\bar{x}}_{\text{lip}}=0.5$ (*purple*) and the relative lipid volume fraction following a truncated normal distribution $x_{\text{lip}}∼T\left({\bar{x}}_{\text{lip}}=0.5,\Delta x_{\text{lip}}=0.1\right)$ (*cyan*; see main text). The MC simulations were performed for a water volume fraction following a truncated normal distribution $\varphi_{1}∼T\left({\bar{\varphi }}_{1}=0.9,\Delta \varphi_{1}=0.1\right)$, $N_{0}=10^{5}$ and $N_{v}=10^{3}$.

The partial derivatives in Eq. 21 are given in the Supporting Material (Eqs. S19 and S20). A visual depiction of Eq. 21 in dependence of the number of voxelinos per voxel $N_{0}=N_{s}/\left(1-\varphi_{1}\right)$ and the corresponding results of MC simulations for certain parameter configurations are shown in Fig. 3 *A*.

As becomes apparent, the analytical solution employing Gaussian propagation of uncertainty is in concordance with the simulated values. However, we note that this is due to the assumption of normal distributions for the individual effective RI increments and PSVs. For nonnormal distributions, Gaussian propagation of uncertainty might not be applicable.

By assuming the mixture distribution given by Eq. 19, consequently, the deviations $\Delta \alpha_{\text{eff}}$ and $\Delta \theta_{\text{eff}}$ are maximized for ${\bar{x}}_{\text{lip}}=0.5$. Secondly, although for nonfluctuating $x_{\text{lip}}$ (i.e., $\Delta x_{\text{lip}}=0$) the respective deviations of the effective RI increment and the PSV exhibit the $1/\sqrt{N_{0}}$ proportionality, for $\Delta x_{\text{lip}}>0$ we obtain nonvanishing deviations for a large number of voxelinos per voxel $N_{0}$.

Throughout this study we assume that all volume fractions, in particular $x_{\text{lip}}$, follow a normal distribution, truncated in the domain [0,1], since values outside of this interval are nonphysical under the assumption of volume additivity. For a normal distribution with mean *μ* and SD *σ*, the corresponding truncated distribution is denoted by T(μ,σ) (see Supporting Material). We note that another choice of distribution could be given by the beta distribution B(a,b) with shape parameters *a* and *b*, which is inherently bounded between 0 and 1. However, the interpretation of the shape parameters in this context is not as straightforward as the truncated normal distribution. Alternatively, a uniform distribution with a certain domain $\left[x_{\text{lip}}^{\min},x_{\text{lip}}^{\max}\right]$ could be employed. For more than two types of macro molecules, the Dirichlet distribution could be of use. However, we expect a similar qualitative behavior for all mentioned distributions.

#### Solvent volume fraction

Next, we study the uncertainty associated to the solvent volume fraction $\varphi_{1}$, defined in Eq. 13, in dependence of the number of solute voxelinos $N_{s}$ per voxel. By employing Gaussian propagation of uncertainty we find

(22) $$\Delta \varphi_{1}=\left(1-{\bar{\varphi }}_{1}\right)\frac{\Delta N_{s}}{{\bar{N}}_{s}}\text{,}$$

where we used the presumption that the number of voxelinos per voxel $N_{0}$ is constant. From Eq. 22, we obtain that a change in the solvent content from voxel to voxel is directly proportional to a relative change in the number of solute voxelinos per voxel $\Delta N_{s}/{\bar{N}}_{s}$. However, it is not clear whether $N_{s}$ follows a statistical distribution and, if so, how this distribution would be governed by active/passive processes in biological matter. An ingenuous guess is given by the equilibrium assumption that the number of solute voxelinos per voxel $N_{s}$ is binomially distributed as

(23) $$N_{s}∼\text{Bin}\left(N_{0},\left(1-{\bar{\varphi }}_{1}\right)\right)\text{,}$$

which may be interpreted as finding $N_{s}$ out of $N_{0}$ voxelinos in a voxel with a probability of $1-{\bar{\varphi }}_{1}$.

Besides a statistical argument, we may also compute a change in the number of solute voxelinos from voxel to voxel by considering $N_{s}=\left(m_{s}\theta_{\text{eff}}\right)/v_{0}$. Herewith, Eq. 22 may be written as

(24) $$\Delta \varphi_{1}=\left(1-{\bar{\varphi }}_{1}\right)\sqrt{{\left(\Delta \varphi_{1}^{0}\right)}^{2}+{\left(\Delta \varphi_{1}^{\infty }\right)}^{2}}\text{,}$$

with

(25) $$\begin{array}{l} \Delta \varphi_{1}^{0}={\left\{\frac{1}{N_{0}\left(1-{\bar{\varphi }}_{1}\right)}\left[{\bar{\varphi }}_{1}+{\left(\frac{\Delta \theta_{\text{eff}}^{0}}{{\bar{\theta }}_{\text{eff}}}\right)}^{2}\right]\right\}}^{1/2}\text{,}\\ \Delta \varphi_{1}^{\infty }={\left[{\left(\frac{\Delta m_{s}}{{\bar{m}}_{s}}\right)}^{2}+{\left(\frac{\partial {\bar{\theta }}_{\text{eff}}}{\partial {\bar{x}}_{\text{lip}}}\frac{\Delta x_{\text{lip}}}{{\bar{\theta }}_{\text{eff}}}\right)}^{2}\right]}^{1/2}\text{,} \end{array}$$

using the presumption of a constant voxelino volume $\Delta v_{0}=0$. This indicates that, similar to the effective RI increment and PSV, given by Eq. 18, the deviation of the solvent volume fraction $\Delta \varphi_{1}$ has two components; for once $\Delta \varphi_{1}^{0}$, which shows the $1/\sqrt{N_{0}}$ dependence, following the central limit theorem. Second, $\Delta \varphi_{1}^{\infty }$, which connects fluctuations in the solvent volume fraction to fluctuations in the solute mass and/or fluctuations in the solute composition. Hence, $\Delta \varphi_{1}^{\infty }$ may be interpreted as quantification of the degree of inhomogeneity of the sample connected to the amount of solute and its composition. Consequently, for a large number of voxelinos per voxel $N_{0}$, these inhomogeneities become the dominant contribution to the deviation of the solvent volume fraction.

We note that the presumption of a constant voxelino volume $\Delta v_{0}=0$ and a constant number of voxelinos per voxel $\Delta N_{0}=0$ from voxel to voxel is necessitated by the experimental boundary condition that all voxels have the same volume, i.e., $\Delta v_{v}=0$.

#### RI

Considering the previous derivations of the uncertainties of the effective PSV and RI increment and the water volume fraction, we now examine the refractive index of the solution. Based on Eq. 7, we readily obtain an estimate of the uncertainty of *n* by employing Gaussian propagation of uncertainty (neglecting potential correlations) as

(26) $$\Delta n={\left[\sum_{i}{\left(\frac{\partial n}{\partial \beta_{i}}\Delta \beta_{i}\right)}^{2}\right]}^{1/2}\text{,}$$

where the sum is taken over all $\beta =\left\{\alpha_{\text{eff}},\theta_{\text{eff}},\varphi_{1}\right\}$ and the respective deviations $\Delta \beta_{i}$ are given in Eqs. 21 and 24. The graphical representation of Eq. 26 in dependence of $N_{0}$ is given in Fig. 3 *A*.

As a consequence, for a vanishing relative deviation of the solute $\Delta m_{s}/{\bar{m}}_{s}$, the $1/\sqrt{N_{0}}$ scaling behavior of the effective RI increment and PSV determines the scaling behavior of the deviations in the RI Δn. However, for $\Delta m_{s}/{\bar{m}}_{s}>0$, we obtain a constant deviation of the RI for large $N_{0}$, consistent with the broad RI distributions obtained by ODT measurements of different cells and tissues (12,16,21,51) and fluctuations in the water volume fraction measured by SRS (22). Furthermore, although the dependence of Δn on the mean relative lipid volume fraction ${\bar{x}}_{\text{lip}}$ is determined by the deviations of the effective RI increment and PSV, we observe a vanishing impact of the deviation of the relative lipid volume fraction $\Delta x_{\text{lip}}$. This fact is due to the numerically small differences of the mean refractions per gram of the particular choice of proteins and the lipid $\left|{\bar{R}}_{\text{lip}}-{\bar{R}}_{p}\right|\approx 0.05$ in combination with larger differences of the mean PSVs $\left|{\bar{\theta }}_{\text{lip}}-{\bar{\theta }}_{p}\right|\approx 0.35$ (see Fig. S1).

#### MD

Finally, we investigate the uncertainty associated with the MD. From Eq. 15 we obtain the corresponding deviation, employing Gaussian propagation of uncertainty, as

(27) $$\Delta \rho ={\left[{\left(\frac{{\bar{\theta }}_{\text{eff}}\rho_{1}-1}{{\bar{\theta }}_{\text{eff}}}\Delta \varphi_{1}\right)}^{2}+{\left(\frac{{\bar{\varphi }}_{1}-1}{{\bar{\theta }}_{\text{eff}}^{2}}\Delta \theta_{\text{eff}}\right)}^{2}\right]}^{1/2}\text{,}$$

displayed in Fig. 3 *A*. As discussed for Δn, the magnitude of Δρ is affected by the mean relative lipid fraction ${\bar{x}}_{\text{lip}}$ as a consequence of the mixture distribution, given in Eq. 14. Furthermore, we have a nonvanishing deviation of the MD for $\Delta m_{s}/{\bar{m}}_{s}>0$ and large $N_{0}$. To no surprise, we observe a remarkable impact of deviation of the relative lipid fraction $\Delta x_{\text{lip}}$ on Δρ due to the strong scaling with the deviation of the effective PSV $\Delta \theta_{\text{eff}}$.

#### Correlation of RI and MD

Having studied the deviations associated with RI and MD, we next sought to illuminate the correlation between the distributions of the RI contrast δn, and the MD *ρ*, denoted by P(ρ,δn), in dependence of the model parameters introduced earlier. To that aim, we performed MC simulations of Eq. 15 for the case of human proteins and triolein in water for a range of different mean relative lipid and water volume fractions, as shown in Fig. 3 *B*.

Besides the intuitive behavior of ρ(δn) for the cases of ${\bar{x}}_{\text{lip}}=0$ (MD increases with increasing RI) and ${\bar{x}}_{\text{lip}}=1$ (MD decreases with increasing RI), for ${\bar{x}}_{\text{lip}}=0.8$ the MD is roughly constant for all RI values. This feature is quite remarkable since it demonstrates that, for certain solute compositions, the MD is decoupled from the RI for all possible water volume fractions, i.e., $\partial \rho /\partial n=0\Rightarrow \theta_{\text{eff}}\left({\bar{x}}_{\text{lip}}\right)=1/\rho_{1}$. Furthermore, as motivated earlier, and shown in Fig. 3 *C*, measuring a RI distribution (e.g., via ODT) may correspond to a range of different water and relative lipid volume fractions, resulting in drastically different estimates on the distribution of the MD from case to case. This in turn strongly motivates the necessity for detailed knowledge about not only the solute composition but also the solvent content of the sample.

In light of the theoretical implications delineated above, in the next step we want to examine the predictive capabilities of the model for a set of physiological complex mixtures that are well characterized in terms of their solute composition.

### Experimental validation and application

In the following, we scrutinize the applicability of previous findings to a set of well-characterized physiological substances: bovine SM (Millipore 70166) and 20% IL (Sigma-Aldrich I141), commonly used as compounds in tissue-mimicking samples because of their optical properties (52,53,54,55,56,57).

To that aim, we measured the solute-concentration-dependent RI and MD with an Abbe refractometer and a pycnometer, respectively. Both samples are particularly intriguing since they should exhibit different ρ(δn) dependencies; SM mainly consists of lactose and milk proteins, whereas IL is a stabilized emulsion of soybean oil (see Fig. 3 *B* for a reference).

**Figure 4:**
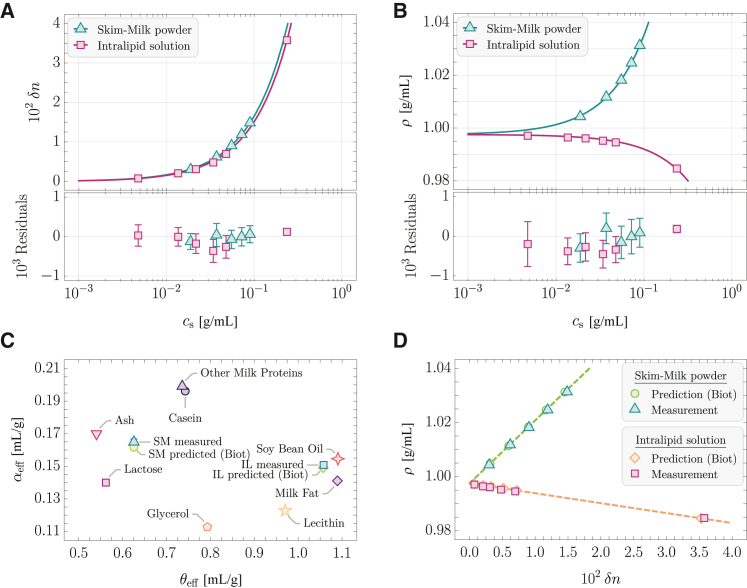
Results of the concentration-dependent measurements of the MD and RI of bovine SM and 20% IL in water as well as the theoretical predictions based on the chemical composition using Eq. 15. (*A*) RI contrast δn in dependence of the solute concentration $c_{s}$ of SM (△, N=5 technical repetitions, mean ± SD) and IL (□, N=5 technical repetitions, mean ± SD) with the respective fits of Eq. 8 (*solid lines*) and fit residuals. (*B*) MD *ρ* in dependence of the solute concentration $c_{s}$ of SM (△, N=5 technical repetitions, mean ± SD) and IL (□, N=5 technical repetitions, mean ± SD) with the respective fits of Eq. 6 (*solid lines*) and fit residuals. (*C*) Effective RI increment $\alpha_{\text{eff}}$ and PSV $\theta_{\text{eff}}$ of various substances that compose SM and IL, as well as the measured and predicted values for SM and IL. (*D*) MD *ρ* in dependence of the RI contrast δn for different concentrations of SM in water and IL. The symbols represent measured values (N=5 technical repetitions, mean ± SD) and the predicted values using the Biot mixing rule (Eq. 7) for $N_{0}=10^{4}$ and $N_{v}=10^{3}$. The dashed lines indicate Eq. 15 for the predicted values of the effective RI increment and PSV.

According to the chemical certificate of analysis provided by the manufacturers, the SM exhibits $y_{1}^{\text{SM}}=4\%$ and the IL exhibits $y_{1}^{\text{IL}}=76\%$ of water. Hence, we computed the respective solute concentrations as $c_{s}^{k}=y_{s}^{k}\rho_{k}$, where $\rho_{k}$ is the measured MD of the sample (SM or IL) at a given dilution, $y_{s}^{k}=m_{k}\left(1-y_{1}^{k}\right)/\left(m_{k}+m_{w}^{k}\right)$ is the solute mass fraction, $m_{k}$ denotes the mass of the sample, and $m_{w}^{k}$ is the mass of water added to the sample. The results of the measurements are shown in Fig. 4 *A* and *B*, where each point represents N=5 technical repetitions.

Using Eqs. 6 and 8, we fitted the data to obtain experimental values of $\theta_{\text{eff}}$ and $\alpha_{\text{eff}}$ with according uncertainties, respectively, for both SM and IL. Examining the fitting residuals, the RI contrast and MD scale linearly with the solute concentration, justifying our model assumptions of the Biot Eq. 7 rule and volume additivity.

We then employed the information about the chemical composition of the respective substances, as provided by the manufacturers (see Table S2), to compute the correlative RI contrast and MD according to Eq. 15, employing MC sampling. A graphical representation of the respective RI increments and PSVs of all substances considered, as well as the experimentally determined and predicted values of SM and IL, is given in Fig. 4 *C*.

As becomes apparent, the measured and predicted values of the SM and IL are in good agreement, whereas potential uncertainties regarding the exact chemical composition might be underappreciated here, as we have no means of estimating them. To further examine the goodness of our model, we compared the solute RIs obtained from fitting the data with various RI mixing rules and the according prediction, based on the chemical composition of the samples, as described in the Supporting Material (Table S3). By this analysis, we found the Biot Eq. 7 to yield the best agreement among a selection of RI mixture rules.

Furthermore, matching the experimental water volume fractions $\varphi_{1}=\left(\rho_{k}-c_{s}^{k}\right)/\rho_{1}$, we obtained a prediction of the MD in dependence of the RI, which was found to coincide well with the measurements for both SM and IL, as shown in Fig. 4 *D*.

#### Larval zebrafish trunk tissue

Having delineated the validity of Eq. 9 in the context of complex mixtures, we examined the capability of the proposed model in an in vivo scenario. To that aim, we chose the larval zebrafish model system at 96 h post fertilization (hpf), for which MS and RI data (employing ODT) of the trunk tissue were recently obtained (21). The RI data are given in Table S4. Because the MS data provide the protein content, we are able to estimate the RI increment and PSV distribution of the proteins present in the tissue, as demonstrated earlier, following Zhao et al. (25) (see Table 1).

In the following, we assume that the larval zebrafish trunk tissue primarily consists of mentioned proteins, lipids, and water, based on the findings of (58,59).

Long et al. (58) determined the wet mass $m_{\text{wet}}$ and dry mass $m_{\text{dry}}$ of larval zebrafish at 96 hpf by weighting (dried) pooled larvae (see their Table 1). Furthermore, they estimated the protein mass $m_{p}$ and lipid mass $m_{\text{lip}}$ of larvae by measuring the optical absorbance using bovine serum albumin and corn oil as calibration materials, respectively. Notably, the measurements of Long et al. (58) were performed on whole animals, including the yolk sac, which is rich in lipids. Hence, the assumed relative volume fraction of the lipids and the water volume fraction are likely to be different from the trunk tissue.

The lipid composition of zebrafish larvae was determined by Hachicho et al. (59), from which we adopted the four phospholipid fatty acids (PLFAs) with the highest abundance. The respective relative volume fractions of the PLFAs for zebrafish larvae at 96 hpf were roughly digitally obtained from Fig. 3 of Hachicho et al. (59) and are provided in Table S2. These four PLFAs make up about 72% of the total PLFA amount in the zebrafish larva. Additionally, based on (59), we assumed that the overall lipid composition of the tissue is only given by triolein and mentioned PLFAs.

**Figure 5:**
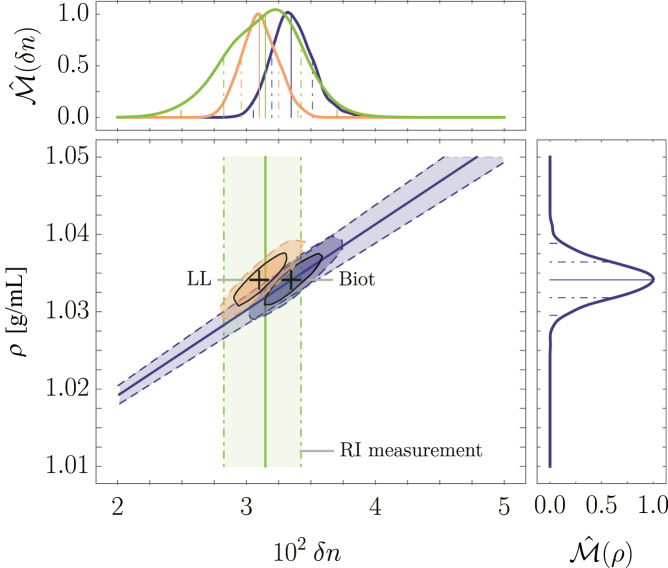
Results of MC simulations and according analytical solutions for the trunk tissue of larval zebrafish. Predicted correlative distributions of the MD *ρ* and the RI contrast δn of larval zebrafish trunk tissue with the corresponding normalized marginal probability density distributions $\hat{M}\left(\rho \right)$ and $\hat{M}\left(\delta n\right)$, respectively (the solid line represents the median, the dash-dotted and dashed lines indicate the 68% and 95% CIs). The ellipsoids indicate the predictions using Eq. 9 (blue) and the Lorentz-Lorenz mixing rule of RIs Eq. S8 (*orange*). The green band indicates the RI measurement of (21). The MC simulations were performed for $N_{0}=10^{5}$ and $N_{v}=10^{3}$.

Using these estimations of the biochemical composition of the larvae and the corresponding material properties given in Table S2, we inferred the distributions of the relative lipid volume fraction $x_{\text{lip}}=0.220\pm 0.022$ and the water volume fraction $\varphi_{1}=0.860\pm 0.006$ (Eqs. S23 and S24)). Employing Eq. 15, we obtained the correlative RI and MD distribution P(δn,ρ) by MC sampling shown in Fig. 5. We then computed the marginalized distributions $\hat{M}\left(\delta n\right)$ and $\hat{M}\left(\rho \right)$ from which we obtained the following median values and 68% confidence intervals (CIs) ρ=(1.0341±0.0024)g/mL and $n_{B}=1.3675\pm 0.0017$. Comparing $n_{B}$ with the RI measurements $n_{\text{meas}}=1.3655_{-0.0032}^{+0.0028}$, evidently, although both values coincide within the 68% CIs, our prediction yields a narrower distribution compared to the measurement, which may be attributed to the narrow assumed distribution of $\varphi_{1}$. Using the Lorentz-Lorenz mixing rule of RIs (Eq. S8), we obtained $n_{\text{LL}}=1.3650\pm 0.0014$, which coincides better with the measured value. However, as pointed out earlier, the Lorentz-Lorenz mixing rule did not yield results that coincided well with the measurements of the validation samples shown above.

In essence, at present, it is not clear which RI mixing rule should be employed in this in vivo scenario without a more comprehensive and quantitative understanding of the biochemical composition of the sample. However, once this insight becomes available (e.g., by measuring (S)RS), different mixing rules could be compared to each other, as presented earlier. This in turn would also allow us to study whether the best-fitting RI mixing rule is conserved across different specimen.

Finally, we want to point out that, if we use the customary simplifying assumption that the solute composition of the trunk tissue is only given by proteins, employing Eq. 9, the RI increment and PSV given in Table 1 and the measured RI data (Table S4), we obtain $\rho_{p}=\left(1.040\pm 0.004\right)\;g/\text{mL}$, using Gaussian propagation of uncertainty. Apparently, this value does not coincide well with the value determined above. In fact, as outlined earlier, not taking lipids into account results in a systematic overestimation of the MD of biological matter.

## Discussion

Considering the complexity of the chemical composition of biological matter, to infer the optical MD from an RI measurement is not well established in contemporary literature. Here, we present a theoretical macroscopic model that is capable of describing the problem, employing a minimal set of assumptions, namely the Biot mixing rule of RIs and the assumption of volume additivity.

We evaluated the possible sources of uncertainties associated with the model and showed that, based on the chemical composition of the sample and the associated degree of inhomogeneity, the resulting RI and MD distributions might drastically differ from the customary assumption of biological matter consisting of proteins and water only. For that purpose, we provided analytical solutions and consistent simulation results for the case of a binary solute composed of proteins and lipids.

Although it is shown that, for singular proteins in solution, the assumption of volume additivity might not be justified (60) (see Supporting Material), we provided experimental evidence that, for the set of validation samples under investigation (bovine SM and 20% IL), it holds well within the measurement uncertainties.

Further, we provided evidence that the predictions of the correlative MD and RI based on our model agree with the experimentally obtained values of the validation samples, thus establishing confidence in the application of the theoretical considerations presented here to estimate MD in dependence of the RI, given the chemical composition of the sample under study.

When applying the model to an in vivo specimen (i.e., the trunk tissue of the larval zebrafish), we observed that the mean value of RI measurements of (21) coincides within the 68% CIs of our predictions, which were based on the estimations of the biochemical composition of the tissue, employing the measurements of Long et al. (58), where the authors determined the masses of water, proteins, and lipids of whole animals. The lipid composition of the tissue was estimated based on the measurements of Hachicho et al. (59). Although our initial predictions are remarkably close to the measurements, given the multitude of assumptions and simplifications about the biochemical composition of the tissue employed, we cannot completely exclude crude uncertainties.

Going forward, the estimation of the MD of biological matter from RI measurements, as outlined in this study, will have interesting implications for inferring the mechanical properties from opto-acoustical measurements (e.g., via Brillouin microscopy). The problem of a varying solute composition within a sample is well appreciated (see, e.g., (12)) but is not resolved in a cohesive manner. Evidently, combining Brillouin microscopy not only with ODT but also incorporating the local biochemical composition (e.g., via (S)RS measurements) allows for a better estimation of the longitudinal (elastic) modulus. We note that the aforementioned problem could also be resolved by performing stimulated Brillouin microscopy in combination with ODT. Here, the MD can be obtained directly from measurements of the Brillouin resonance gain factor, which in turn is connected to the Brillouin gain and the pump laser power, as well as the RI, Brillouin frequency shift, and Brillouin line width (61). Accurately determining the local (in vivo) MD will conceivably enable a more profound interpretation of functional mechanisms at play in biological matter.

To make the application of the findings of this study more accessible, we delineate strategies on how to estimate MD, given certain experimental paradigms, in the Supporting Material (see Fig. S2).

## Data and code availability

The data and code that support the findings of this study are available upon reasonable request from the authors.

## Author contributions

J.G., C.M., and T.B. conceptualized the project. C.M. developed the methodology and conducted the experiments. C.M., J.K., and D.W. performed formal analysis of the data. All authors interpreted the experimental results. C.M. conducted the theoretical considerations. C.M., T.B., S.K., S.A., and V.Z. interpreted the theoretical results. C.M. wrote the original draft of this manuscript. All authors edited and improved the manuscript. J.G., D.W., and V.Z. supervised the project.

## Supporting citations

The following references appear in the Supporting Material (62,63,64,65,66,67(c),67(d),67(e),67(a),67(b),68(b),68(a)).

## Supporting material

Document S1. Figures S1 and S2 and Tables S1–S4

Document S2. Article plus supporting material

## References

[1] Guck J., Schinkinger S., Bilby C. (2005). Optical Deformability as an Inherent Cell Marker for Testing Malignant Transformation and Metastatic. CompetenceBiophys. J..

[2] Engler A.J., Sen S., Discher D.E. (2006). Matrix Elasticity Directs Stem Cell Lineage Specification. Cell.

[3] Egan P., Sinko R., Keten S. (2015). The role of mechanics in biological and bio-inspired systems. Nat. Commun..

[4] Biswas A., Munoz O., Reber S. (2023). Conserved nucleocytoplasmic density homeostasis drives cellular organization across eukaryotes. bioRxiv.

[5] Iida S., Ide S., Maeshima K. (2024). Orientation-Independent-DIC imaging reveals that a transient rise in depletion force contributes to mitotic chromosome condensation. bioRxiv.

[6] Watson J.L., Seinkmane E., Derivery E. (2023). Macromolecular condensation buffers intracellular water potential. Nature.

[7] Schürmann M., Scholze J., Chan C.J. (2016). Cell nuclei have lower refractive index and mass density than cytoplasm. J. Biophot..

[8] Biswas A., Kim K., Reber S. (2021). The Xenopus spindle is as dense as the surrounding cytoplasm. Dev. Cell.

[9] Antonacci G., Braakman S. (2016). Biomechanics of subcellular structures by non-invasive Brillouin microscopy. Sci. Rep..

[10] Yan G., Monnier S., Dehoux T. (2022). Probing molecular crowding in compressed tissues with Brillouin light scattering. Proc. Natl. Acad. Sci. USA.

[11] Scarcelli G., Polacheck W.J., Yun S.H. (2015). Noncontact three-dimensional mapping of intracellular hydromechanical properties by Brillouin microscopy. Nat. Methods.

[12] Schlüßler R., Kim K., Guck J. (2022). Correlative all-optical quantification of mass density and mechanics of subcellular compartments with fluorescence specificity. Elife.

[13] Kennedy B.F., Bamber J.C. (2021). “emph ”bibinfo booktitle Optical Coherence Elastography: Imaging Tissue Mechanics on the Micro-Scale.

[14] Larin K.V., Sampson D.D. (2017). Optical coherence elastography – OCT at work in tissue biomechanics [Invited]. Biomed. Opt Express.

[15] Park Y., Depeursinge C., Popescu G. (2018). Quantitative phase imaging in biomedicine. Nat. Photonics.

[16] Kim K., Guck J. (2020). The Relative Densities of Cytoplasm and Nuclear Compartments Are Robust against Strong Perturbation. Biophys. J..

[17] BARER R. (1952). Interference Microscopy and Mass Determination. Nature.

[18] Bhaduri B., Pham H., Popescu G. (2012). Diffraction phase microscopy with white light. Opt. Lett..

[19] Schlüßler R., Möllmert S., Guck J. (2018). Mechanical Mapping of Spinal Cord Growth and Repair in Living Zebrafish Larvae by Brillouin Imaging. Biophys. J..

[20] Rollin R., Joanny J.-F., Sens P. (2023). Physical basis of the cell size scaling laws. Elife.

[21] Kolb J., Tsata V., Wehner D. (2023). Small leucine-rich proteoglycans inhibit CNS regeneration by modifying the structural and mechanical properties of the lesion environment. Nat. Commun..

[22] Oh S., Lee C., Kirschner M.W. (2022). Protein and lipid mass concentration measurement in tissues by stimulated Raman scattering microscopy. Proc Natl Acad Sci USA.

[23] Alunni Cardinali M., Di Michele A., Fioretto D. (2022). Brillouin–Raman microspectroscopy for the morpho-mechanical imaging of human lamellar bone. J. R. Soc. Interface.

[24] Abuhattum S., Kotzbeck P., Taubenberger A.V. (2022). Adipose cells and tissues soften with lipid accumulation while in diabetes adipose tissue stiffens. Sci. Rep..

[25] Zhao H., Brown P.H., Schuck P. (2011). On the distribution of protein refractive index increments. Biophys. J..

[26] W. R. Inc. (2020).

[27] Durchschlag H. (1986).

[28] Heller W., The Journal of Physical Chemistry (1965). Remarks on Refractive Index Mixture Rules. J. Phys. Chem..

[29] Hanley B.F. (2020). A practical method for estimating specific refractive index increments for flexible non-electrolyte polymers and copolymers in pure and mixed solvents using the Gladstone-Dale and Lorentz-Lorenz equations in conjunction with molar refraction structural constants, and solvent physical property databases. Mater. Today Commun..

[30] PERKINS S.J. (1986). Protein volumes and hydration effects. The calculations of partial specific volumes, neutron scattering matchpoints and 280-nm absorption coefficients for proteins and glycoproteins from amino acid sequences. Eur. J. Biochem..

[31] Aknoun S., Yonnet M., Pognonec P. (2021). Quantitative phase microscopy for non-invasive live cell population monitoring. Sci. Rep..

[32] Rogers J.D., Radosevich A.J., Backman V. (2014). Modeling Light Scattering in Tissue as Continuous Random Media Using a Versatile Refractive Index Correlation Function. IEEE J. Sel. Top. Quant. Electron..

[33] Mir M., Wang Z., Popescu G. (2011). Optical measurement of cycle-dependent cell growth. Proc. Natl. Acad. Sci. USA.

[34] Popescu G., Park Y., Badizadegan K. (2008). Optical imaging of cell mass and growth dynamics. Am. J. Physiol. Cell Physiol..

[35] Cooper K.L., Oh S., Tabin C.J. (2013). Multiple phases of chondrocyte enlargement underlie differences in skeletal proportions. Nature.

[36] Barr E.S. (1955). Concerning Index of Refraction and Density. Am. J. Phys..

[37] Consortium T.U. (2022). Nucleic Acids Res. UniProt: the Universal Protein Knowledgebase in 2023.

[38] Mayerhöfer T.G., Popp J. (2020). Beyond Beer's Law: Revisiting the Lorentz-Lorenz Equation. ChemPhysChem.

[39] C. E. J. (1943). Proteins, Amino Acids and Peptides as Ions and Dipolar Ionsnewspace.

[40] Pretorius F., Focke W.W., du Toit E. (2021). Estimating binary liquid composition from density and refractive index measurements: A comprehensive review of mixing rules. J. Mol. Liq..

[41] Reis J.C.R., Lampreia I.M.S., Douhéret G. (2010). Refractive Index of Liquid Mixtures: Theory and Experiment. ChemPhysChem.

[42] Brocos P., Piñeiro Á., Amigo A. (2003). Refractive indices, molar volumes and molar refractions of binary liquid mixtures: concepts and correlations. Phys. Chem. Chem. Phys..

[43] Sihvola A. (2000).

[44] Acevedo-Barrera A., Garcia-Valenzuela A. (2019). Analytical approximation to the complex refractive index of nanofluids with extended applicability. Opt Express.

[45] Meiers D.T., von Freymann G. (2023). Mixing rule for calculating the effective refractive index beyond the limit of small particles. Opt Express.

[46] Garahan A., Pilon L., Saxena I. (2007). Effective optical properties of absorbing nanoporous and nanocomposite thin films. J. Appl. Phys..

[47] Smith D., Myers J.S., Goodman-Strauss C. (2023).

[48] Chorlay A., Thiam A.R. (2020). Neutral lipids regulate amphipathic helix affinity for model lipid droplets. J. Cell Biol..

[49] Onal G., Kutlu O., Dokmeci Emre S. (2017). Lipid Droplets in Health and Disease. Lipids Health Dis..

[50] Thiam A.R., Ikonen E. (2021). Lipid Droplet Nucleation. Trends Cell Biol..

[51] Kim K., Gade V.R., Guck J. (2022). Quantitative imaging of Caenorhabditis elegans dauer larvae during cryptobiotic transition. Biophys. J..

[52] Vidallon M.L.P., Salimova E., Bishop A.I. (2022). Enhanced photoacoustic imaging in tissue-mimicking phantoms using polydopamine-shelled perfluorocarbon emulsion droplets. Ultrason. Sonochem..

[53] Lai P., Xu X., Wang L.V. (2014). Dependence of optical scattering from Intralipid in gelatin-gel based tissue-mimicking phantoms on mixing temperature and time. J. Biomed. Opt..

[54] Cook J.R., Bouchard R.R., Emelianov S.Y. (2011). Tissue-mimicking phantoms for photoacoustic and ultrasonic imaging. Biomed. Opt Express.

[55] Bachir W., khir R. (2022). Characterization of pasteurized milk in the near infrared range for construction of tissue-mimicking optical phantoms. Opt. Mater. X.

[56] Stiles T.A., Madsen E.L., Lucey J.A. (2005). Tissue-Mimicking Liquid for Use in Exposimetry. J. Ultrasound Med..

[57] McGarry C.K., Grattan L.J., Clark C.H. (2020). Tissue mimicking materials for imaging and therapy phantoms: a review. Phys. Med. Biol..

[58] Long Y., Li L., Cui Z. (2012). Transcriptomic Characterization of Temperature Stress Responses in Larval Zebrafish. PLoS One.

[59] Hachicho N., Reithel S., Luckenbach T. (2015). Body Mass Parameters, Lipid Profiles and Protein Contents of Zebrafish Embryos and Effects of 2,4-Dinitrophenol Exposure. PLoS One.

[60] Zamyatnin A. (1972).

[61] Boyd R.W., Boyd R.W. (2008). “emph ”bibinfo booktitle Nonlinear Optics.

[62] Kim S., Chen J., Bolton E.E. (2022). PubChem 2023 update. Nucleic Acids Res..

[63] (2022).

[64] McMeekin T.L., Wilensky M., Groves M.L. (1962). Refractive indices of proteins in relation to amino acid composition and specific volume. Biochem. Biophys. Res. Commun..

[65] Bansal N., Truong T., Bhandari B. (2017). Feasibility study of lecithin nanovesicles as spacers to improve the solubility of milk protein concentrate powder during storage. Dairy Sci. Technol..

[66] Oxford Lab Fine Chem LLP. F020Soybean lecithin. https://www.oxfordlabchem.com/msds/(S-08441)SOYA%20LECITHIN%2030%20Extra%20Pure.pdf.

[bib267a] ChemSpider. Triolein, CSID:4593733. http://www.chemspider.com/Chemical-Structure.4593733.html.

[bib267b] ChemSpider. Palmitic acid , CSID:960. http://www.chemspider.com/Chemical-Structure.960.html.

[bib267c] ChemSpider. Oleic acid, CSID:393217. http://www.chemspider.com/Chemical-Structure.393217.html.

[bib267d] ChemSpider. Docosahexaenoic acid, CSID:393183. http://www.chemspider.com/Chemical-Structure.393183.html.

[bib267e] ChemSpider. Stearic acid, CSID:5091. http://www.chemspider.com/Chemical-Structure.5091.html.

[bib268a] Merck. Soybean oil, 47122. https://www.sigmaaldrich.com/DE/de/product/supelco/47122,

[bib268b] Merck. Glycerol, 104057. https://www.merckmillipore.com/DE/de/product/Glycerol,MDA_CHEM-104057.

