# Supplementary material for: Estimation of the mass density of biological matter from refractive index measurements
Source: Biophys Rep (N Y). 2024 Apr 24;4(2):100156. doi: 10.1016/j.bpr.2024.100156 (PMC11090064; doi:10.1016/j.bpr.2024.100156)
Supplement: Document S1. Figures S1 and S2 and Tables S1–S4 [file mmc1.pdf]

Biophysical Reports, Volume *4*

Supplemental information

Estimation of the mass density of biological matter from refractive index measurements

Conrad Möckel, Timon Beck, Sara Kaliman, Shada Abuhattum, Kyoohyun Kim, Julia Kolb, Daniel Wehner, Vasily Zaburdaev, and Jochen Guck

Supporting Material: Estimations of the mass density of biological matter from refractive index measurements

I. PARTIAL AND APPARENT SPECIFIC VOLUMES AND THE REFRACTIVE INDEX INCREMENT

Based on derivations in [1], for a binary solution consistent of N_1 moles of solvent with molar volume V_1 and N_2 moles of solute, the total molar volume is given by

$$\begin{aligned} V &= N_1 V_1 + N_2 \tilde{\Theta}, \text{ or} \\ \tilde{\Theta} &= \frac{V - N_1 V_1}{N_2}, \end{aligned} \tag{S1}$$

where $\tilde{\Theta}$ denotes the apparent molar volume of the solute. This parameter accounts for the potential volume-inadditivity; in other words, it is a macroscopic quantity that describes the volume of the solute in dilution. The partial molar volume is given by

$$\Theta = \left(\frac{\partial V}{\partial N_2}\right)_{T,p,N_1} = N_2 \left(\frac{\partial \tilde{\Theta}}{\partial N_2}\right)_{T,p,N_1} + \tilde{\Theta}, \tag{S2}$$

and the apparent and partial specific volume can be expressed with the molar mass of the solute M_2 as $\vartheta = \tilde{\Theta}/M_2$ and $\theta = \Theta/M_2$, respectively.

A. Volume additivity

If the volume additivity is given, then

$$\begin{aligned} \Theta = V_2 = \tilde{\Theta} &= \frac{M_2}{\rho_2}, \\ \theta = \vartheta = 1/\rho_2 &= \frac{V_2 N_{\mathrm{A}}}{M_2}. \end{aligned} \tag{S3}$$

B. Protein PSV and RI increment

As employed in [1–4], the PSV and refraction per gram of a protein may be interpreted the weight average of all amino acids composing this protein as

$$\begin{aligned} \theta_{\mathrm{p}} &= \frac{\sum_i V_{\mathrm{r},i} N_{\mathrm{A}}}{\sum_i M_{\mathrm{r},i} + M_{\mathrm{w}}}, \\ R_{\mathrm{p}} &= \frac{\sum_i R^*_{\mathrm{r},i}}{\sum_i M_{\mathrm{r},i} + M_{\mathrm{w}}}, \end{aligned} \tag{S4}$$

where $M_{\mathrm{w}} = 18.01$ g/mol is the molecular mass of water [5, 6] and N_{A} is the Avogadro constant. We note that, without loss of generality, setting $m_i = M_i/N_{\mathrm{A}}$, i.e. $n_i = 1/N_{\mathrm{A}}$, Eq. (S4) can be expressed as

$$\begin{aligned} \theta_{\mathrm{p}} &= \sum_i \frac{m_{\mathrm{r},i}\theta_{\mathrm{r,i}}}{m_{\mathrm{p}}}, \\ R_{\mathrm{p}} &= \sum_i \frac{m_{\mathrm{r},i} R_{\mathrm{r,i}}}{m_{\mathrm{p}}}, \end{aligned} \tag{S5}$$

which coincides with the Lorentz–Lorenz mixing rule. This can be seen by

$$R^*_{\mathrm{p}} = \sum_i a_i R^*_{\mathrm{r},i}, \tag{S6}$$

where a_i is the respective mole fraction.

TABLE S1: Values of amino acid residues (AAR) employed for calculating the PSV θ and RI increment for Wiener approximation and volume additivity argument α_r^W and α_r^B, respectively. See text for further details. Values obtained from §[5, 6], †[3] (consensus average), ‡[7].

AAR	$M_r^§$ in g/mol	$V_r^†$ in 10^{-3}nm^3	θ_r in ml/g	$R_r^{*‡}$ in cm^3	α_r^W in ml/g	α_r^B in ml/g
Arg	156.19	188 ± 10	0.73 ± 0.04	39.47 ± 0.10	0.194 ± 0.016	0.202 ± 0.018
His	137.14	156 ± 6	0.686 ± 0.027	34.62 ± 0.15	0.210 ± 0.012	0.221 ± 0.014
Lys	128.17	173 ± 6	0.811 ± 0.028	34.10 ± 0.20	0.184 ± 0.012	0.191 ± 0.013
Asp	115.09	115.4 ± 2.2	0.604 ± 0.012	26.06	0.193 ± 0.005	0.204 ± 0.006
Glu	129.12	141 ± 5	0.657 ± 0.025	30.07 ± 0.15	0.183 ± 0.011	0.192 ± 0.012
Ser	87.08	91.7 ± 1.8	0.634 ± 0.012	19.16 ± 0.10	0.168 ± 0.006	0.175 ± 0.006
Thr	101.10	118.3 ± 2.3	0.705 ± 0.014	23.82 ± 0.10	0.169 ± 0.006	0.175 ± 0.007
Asn	114.10	120 ± 4	0.634 ± 0.022	26.09 ± 0.20	0.185 ± 0.010	0.194 ± 0.011
Gln	128.13	145 ± 5	0.682 ± 0.024	30.37 ± 0.20	0.181 ± 0.011	0.189 ± 0.012
2Cys	204.26	211 ± 10	0.621 ± 0.029	48.58	0.209 ± 0.013	0.221 ± 0.016
Gly	57.05	59.9 ± 2.2	0.632 ± 0.023	12.81 ± 0.10	0.177 ± 0.011	0.186 ± 0.012
Pro	97.12	123.2 ± 1.8	0.764 ± 0.011	23.74 ± 0.10	0.161 ± 0.005	0.167 ± 0.005
Ala	71.08	87.8 ± 2.3	0.744 ± 0.019	17.15 ± 0.15	0.164 ± 0.009	0.169 ± 0.010
Ile	113.16	166.1 ± 3.4	0.884 ± 0.018	31.87 ± 0.20	0.184 ± 0.008	0.191 ± 0.009
Leu	113.16	168 ± 4	0.894 ± 0.023	31.59 ± 0.15	0.176 ± 0.010	0.181 ± 0.010
Met	131.19	165.2 ± 1.8	0.758 ± 0.008	34.45 ± 0.05	0.199 ± 0.004	0.208 ± 0.004
Phe	147.18	190 ± 7	0.776 ± 0.030	42.21 ± 0.15	0.240 ± 0.013	0.253 ± 0.016
Trp	186.21	228 ± 4	0.737 ± 0.012	55.24 ± 0.30	0.278 ± 0.007	0.297 ± 0.008
Tyr	163.18	191 ± 8	0.706 ± 0.030	44.34	0.240 ± 0.013	0.255 ± 0.016
Val	99.13	139 ± 4	0.843 ± 0.022	26.73 ± 0.13	0.178 ± 0.009	0.184 ± 0.010

C. Errors with assuming volume additivity

- Errors in the PSV considerations
  - The following effects are stated in [1]
    - conformational protein formation from amino acids: ~ 0.012 ml/g
    - volume changes of proteins in solution:
      - swelling and dissolution: $\sim -5 \times 10^{-2}$ ml/g
      - thermal denaturation: $\sim \pm 10^{-2}$ ml/g
      - Coil-helix transition: $\sim +10^{-2}$ ml/g
      - Aggregation: $\sim +5 \times 10^{-3}$ ml/g
      - Sol-gel transition: $\sim -10^{-4}$ ml/g
- Errors in the RI increment considerations
  - [4] found that the predicted RI increment values using the Wiener relation for different proteins are systematically higher than the experimental values. Further they found a linear relationship between predicted and experimental values. Further they propose a $\pi - \pi$ interaction term correction which slightly improves the agreement. We do not employ this correction here. However, we note that this discrepancy could also be partially resolved by not employing the Wiener equation but rather relying on the Biot equation, as well as a different choice of experimental values of R_r^* for the amino acid residues.

D. Employed amino acid residues values

We omitted the contributions of selenocysteine since we could not obtain data on molar refractivity, mass density nor refractive index. For all other amino acid residues (AAR) the employed values are given in table S1.

II. PARAMETER VALUES

In the following we provide the mean values ± standard deviations of the following quantities; relative volume fraction x, refractive index n, refraction per gram R in cm^3/g, PSV θ in ml/g and RI increment α in ml/g of the

different macro molecules employed throughout this study in Table S2. The RI and refraction per gram values of the lipids employed in the sections *Lipids and proteins in water* and *Larval zebrafish trunk tissue* were obtained from [8] (predicted data by `ACD/Labs Percepta Platform - PhysChem Module`, at $T = 20\,^{\circ}\mathrm{C}$ and a wavelength of $\lambda = 589\,\mathrm{nm}$), from which we computed the PSV *via* the Lorentz–Lorenz relation (Eq. (10)). The associated standard deviation was then estimated *via* Gaussian propagation of uncertainty. For all the computations presented in this study, we interpret a value that has a uncertainty attached to it as a normal distribution, where the standard deviation is given by said uncertainty. For values that were assumed to be precise, we assume a delta distribtuion.

TABLE S2: Rounded mean values ± standard deviations of relative volume fraction x, refractive index n, refraction per gram R in cm^3/g, PSV θ in ml/g and RI increment α in ml/g of the different macro molecules employed throughout this study. Entries without footnote indicate assumptions or derived values (see main text for further information). Assumed to be precise values are stated without standard deviation.

	$\overline{x} \pm \Delta x$	$\overline{n} \pm \Delta n$	$\overline{R} \pm \Delta R$ in cm^3/g	$\overline{\theta} \pm \Delta\theta$ in ml/g	$\overline{\alpha} \pm \Delta\alpha$ in ml/g
Water		1.334		1/0.997	
Lipids and proteins in water					
Proteins (human proteome)	$(1 - \overline{x}_{\mathrm{lip}}) \pm \Delta x_{\mathrm{lip}}$	1.603 ± 0.007[a]	0.2520 ± 0.0033[a]	0.734 ± 0.012[a]	0.197 ± 0.004[a]
Triolein (neutral lipid)	$\overline{x}_{\mathrm{lip}} \pm \Delta x_{\mathrm{lip}}$	1.477[b]	0.30674 ± 0.00034[b]	1.086 ± 0.0012	0.1552 ± 0.0008
Bovine skim milk					
Proteins	0.33[g]				
Caseins	0.20[c]	1.597 ± 0.006[a]	0.2531 ± 0.0006[a]	0.743 ± 0.007[a]	0.1954 ± 0.0029[a]
α_{S1}-Casein	0.42[c]	1.600[a]	0.253[a]	0.740[a]	0.198[a]
α_{S2}-Casein	0.11[c]	1.602[a]	0.253[a]	0.737[a]	0.199[a]
β-Casein	0.35[c]	1.588[a]	0.254[a]	0.753[a]	0.192[a]
κ-Casein	0.12[c]	1.597[a]	0.252[a]	0.740[a]	0.195[a]
Other proteins	0.08[c]	1.604 ± 0.007[a]	0.2530 ± 0.0030[a]	0.736 ± 0.011[a]	0.198 ± 0.004[a]
Fat	0.01[g]	1.462[c]	0.299	1.09[c]	0.141
Lactose	0.52[g]	1.582	0.187	0.562[c]	0.140[c]
Ash	0.06[g]	1.647	0.196	0.541[c]	0.170[c]
Intralipid solution					
Soybean oil	0.89[g]	1.474[h]	0.31	1.09[h]	0.15
Glycerol	0.07[g]	1.474[h]	0.22	0.79[h]	0.11
Lecithin	0.05[g]	1.459[i]	0.27	0.97[j]	0.12
Larval zebrafish trunk tissue					
Proteins (trunk tissue)	0.780 ± 0.022[d]	1.602 ± 0.005[e]	0.2520 ± 0.0025[e]	0.734 ± 0.009[e]	0.1971 ± 0.0033[e]
Lipids	0.220 ± 0.022[d]	1.473 ± 0.016	0.308 ± 0.06	1.100 ± 0.023	0.153 ± 0.016
Triolein	0.28[f]	1.477[b]	0.30674 ± 0.00034[b]	1.0856 ± 0.0012	0.1552 ± 0.0008
Palmitic acid	0.24[f]	1.454[b]	0.3030 ± 0.0012[b]	1.119 ± 0.004	0.1343 ± 0.0029
Oleic acid	0.20[f]	1.467[b]	0.3084 ± 0.0011[b]	1.111 ± 0.004	0.1478 ± 0.0026
Docosahexaenoic acid	0.15[f]	1.521[b]	0.3224 ± 0.0009[b]	1.0587 ± 0.0030	0.1980 ± 0.0023
Stearic acid	0.12[f]	1.456[b]	0.3058 ± 0.0011[b]	1.125 ± 0.004	0.1373 ± 0.0026

[a] computed from AA sequences obtained from [9]
[b] obtained from [8]
[c] obtained from [10]
[g] obtained from the manufacturer
[h] obtained from [11]
[i] obtained from [12]
[j] obtained from [13]
[d] computed from values obtained from [14]
[e] computed from AA sequences obtained from [15] and [9]
[f] computed from values obtained from [16]

III. REFRACTIVE INDEX MIXING MODEL COMPARISONS

Following [17, 18], assuming volume additivity, we may express the different mixing rules as

$$f_i(n) = (1-\varphi_\mathrm{s})f_i(n_1) + \varphi_\mathrm{s} f_i(n_\mathrm{s}), \tag{S7}$$

where the different f_i are specific for each mixing rule, φ_s and n_s denotes the solute volume fraction and RI, respectively. The f_i under study are listed in table S3.

By solving Eqs. (S7) of the respective mixing rule for the mixture RI, we obtain a functional relationship $n(n_\mathrm{s}, c_\mathrm{s}, \rho_\mathrm{s})$ that can be used to fit experimental data, as shown in Fig. 4A. Since we obtained the solute MD ρ_s from measurements of the mixture MD in dependence of c_s (see Fig. 4B), by fitting the data with $n(n_\mathrm{s}, c_\mathrm{s}, \rho_\mathrm{s})$, we obtain a value of solute RI n_s for each model. We then evaluate the difference between the respective fitted solute RIs and the -based on the biochemical composition- predicted solute RI of the samples under study, using different mixing rules. The results are given in Table S3.

TABLE S3: f_i as defined in Eq. (S7) for different mixing rules and difference between fitted and predicted solute RIs $n_\mathrm{s}^\mathrm{fit} - n_\mathrm{s}^\mathrm{pred}$ for the respective RI mixing rules. The uncertainty intervals were computed employing Gaussian propagation of uncertainty. The predicted RI distributions were obtain as outlined in the main text for $N_0 = 10^3$ and $N_\mathrm{v} = 10^3$. RI Mixture rules were adapted from [17].

		$n_\mathrm{s}^\mathrm{fit} - n_\mathrm{s}^\mathrm{pred}$	
Mixing rule	$f(n)$	Skim milk	20 % intralipid emulsion
Biot	n	-0.005 ± 0.006	-0.0014 ± 0.0023
Series	$1/n$	-0.066 ± 0.008	-0.0141 ± 0.0025
Lichtenecker	$\log n$	-0.032 ± 0.007	-0.0074 ± 0.0024
Lorentz–Lorenz	$\left(n^2-1\right)/\left(n^2+2\right)$	-0.032 ± 0.007	-0.0072 ± 0.0024
Wiener	$\left(n^2-n_1^2\right)/\left(n^2+2n_1^2\right)$	-0.016 ± 0.006	-0.0038 ± 0.0023

IV. DERIVATION OF THE MASS DENSITY–REFRACTIVE INDEX RELATION FOR THE LORENTZ-LORENZ MIXING RULE

Following [18], assuming volume additivity, the Lorentz–Lorenz mixing rule of RIs can be expressed as

$$f_\mathrm{LL}(n) = (1-\varphi_2)\, f_\mathrm{LL}(n_1) + \varphi_2 f_\mathrm{LL}(n_2), \tag{S8}$$

with $f_\mathrm{LL}(n_i) \equiv \left(n_i^2-1\right)/\left(n_i^2+2\right)$. Next, we solve above equation for n_LL to obtain

$$n_\mathrm{LL} = \left[\frac{2c_2\left(n_2^2-n_1^2\right)+n_1^2\left(n_2^2+2\right)\rho_2}{c_2\left(n_1-n_2\right)\left(n_1+n_2\right)+\left(n_2^2+2\right)\rho_2}\right]^{-1/2}, \tag{S9}$$

from which we compute the RI increment $\alpha_\mathrm{LL} \equiv \partial n_\mathrm{LL}/\partial c_2$ as

$$\alpha_\mathrm{LL} = \frac{\left(n_1^2+2\right)\left(n_2^2+2\right)\left(n_2^2-n_1^2\right)\rho_2}{2\left[c_2\left(n_1^2-n_2^2\right)+\rho_2\left(n_2^2+2\right)\right]^{3/2}\sqrt{n_1^2\rho_2\left(n_2^2+2\right)-2c_2\left(n_1^2-n_2^2\right)}}. \tag{S10}$$

Now, Eq.(S10) has to be solved for the solute concentration c_2. While this is analytically possible, the resulting expression is quite lengthy, so we refer to the resulting expression as $c_2\left(\alpha_\mathrm{LL}, n_1, n_2, \theta\right)$. Of course, this solving step can be done numerically, given a set of parameter values. Finally, following the rational of the main text, we insert the resulting expression for the solute concentration in Eq. (6) to obtain

$$\rho = \rho_1(1 - c_2\left(\alpha_\mathrm{LL}, n_1, n_2, \theta\right)\theta) + c_2\left(\alpha_\mathrm{LL}, n_1, n_2, \theta\right). \tag{S11}$$

We note that all other considerations presented in the main text can be adapted for Eq. (S11), while the results will be, unfortunately, more lengthy and difficult to handle.

V. PDF OF A TRUNCATED NORMAL DISTRIBUTION $\mathcal{T}$

For a normal distribution with mean μ and standard deviation σ, the corresponding truncated distribution is denoted by $\mathcal{T}(\mu,\sigma)$. The probability density function (PDF) of $\mathcal{T}$ is defined as

$$t(y) \equiv \begin{cases} \frac{f(y)}{F(1)-F(0)} & 0 \le y \le 1 \\ 0 & \text{else} \end{cases}, \tag{S12}$$

where $f(y)$ and $F(y)$ denote the PDF and cumulative distribution function (CDF), respectively, of said normal distribution $\mathcal{N}(\mu,\sigma)$.

VI. MEAN AND STANDARD DEVIATION OF NORMAL MIXTURE DISTRIBUTIONS

Let's consider a one dimensional mixture distribution of two Normal distributions,

$$\mathcal{P}(\mu_1,\mu_2,\sigma_1,\sigma_2;x) = w\mathcal{N}(\mu_1,\sigma_1;x) + (1-w)\mathcal{N}(\mu_2,\sigma_2;x), \tag{S13}$$

with $0 \le w \le 1$. The mean and standard deviation of this mixture distribtion is given by

$$\begin{aligned} \mu' &= w\mu_1 + (1-w)\mu_2, \\ \sigma' &= \sqrt{\sigma_2^2 + w\left(\mu_1^2 - 2\mu_1\mu_2 + \mu_2^2 + \sigma_1^2 - \sigma_2^2\right) - w^2\left(\mu_1^2 - \mu_2^2\right)^2}, \end{aligned} \tag{S14}$$

respectively. For the special case of $\sigma_1 = \sigma_2 = \sigma$ we obtain for the standard deviation of the mixture distribution

$$\sigma' = \sqrt{\sigma^2 - \left((w-1)\,w\,(\mu_1-\mu_2)^2\right)} > \sigma, \tag{S15}$$

for all $\mu_1 \ne \mu_2$ and $0 \le w \le 1$, which is maximized for $w = 0.5$.

VII. EFFECTIVE PARAMETERS

From the mixing rule of the solute density

$$\rho_{\mathrm{s}} = \sum_{i=1}^{N_{\mathrm{s}}} x_{i+1}\rho_{i+1}, \tag{S16}$$

we obtain that the effective PSV is

$$\theta_{\mathrm{eff}} = 1/\rho_{\mathrm{s}} = \left(\sum_{i=1}^{N_{\mathrm{s}}} \frac{x_{i+1}}{\theta_{i+1}}\right)^{-1} = \sum_{i=1}^{N_{\mathrm{s}}} y_{i+1}\theta_{i+1} = \sum_j \frac{N_j m_j}{m_{\mathrm{s}}}\theta_j, \tag{S17}$$

where N_{s} is the nuber of solute voxelinos per voxel and j denotes the different types of solute molecules. The effective RI increment for the Biot equation is then

$$\begin{aligned} \alpha_{\mathrm{eff}} = \frac{n-n_1}{c} &= \frac{n_1\left(1 - c\sum_{i=1}^{N_{\mathrm{s}}} y_{i+1}\theta_{i+1}\right) + c\sum_{i=1}^{N_{\mathrm{s}}} y_{i+1}\theta_{i+1}n_{i+1} - n_1}{c} \\ &= n_1\sum_{i=1}^{N_{\mathrm{s}}} y_{i+1}\theta_{i+1} - \sum_{i=1}^{N_{\mathrm{s}}} n_{i+1}y_{i+1}\theta_{i+1} \\ &= \sum_{i=1}^{N_{\mathrm{s}}}(n_1 - n_{i+1})y_{i+1}\theta_{i+1} \\ &= \sum_{i=1}^{N_{\mathrm{s}}} y_{i+1}\alpha_{i+1} = \sum_j \frac{N_j m_j}{m_{\mathrm{s}}}\alpha_j = \theta_{\mathrm{eff}}\left(n_{\mathrm{s}} - n_1\right). \end{aligned} \tag{S18}$$

VIII. UNCERTAINTIES OF THE EFFECTIVE PARAMETERS FOR LIPIDS AND PROTEINS IN WATER

The uncertainties of the effective RI increment and the PSV, introduced in Eq. (21) can be computed as follows. The terms $\Delta\alpha^0_{\rm eff}$ and $\Delta\theta^0_{\rm eff}$ refer to the standard deviations of the mixture distribution, as given in Eq. (S14).

The uncertainties associated of the effective PSV to deviations in the relative lipid volume fraction $\Delta x_{\rm lip}$, can be evaluated by employing Eq. (16), from which we obtain that

$$\frac{\partial\overline{\theta}_{\rm eff}}{\partial\overline{x}_{\rm lip}} = \frac{\overline{\theta}_{\rm p}\overline{\theta}_{\rm lip}\left(\overline{\theta}_{\rm lip}-\overline{\theta}_{\rm p}\right)}{\left(\left(1-\overline{x}_{\rm lip}\right)\overline{\theta}_{\rm lip}+\overline{x}_{\rm lip}\overline{\theta}_{\rm p}\right)^2}. \tag{S19}$$

We may write the effective RI increment, using Eq. (16), as

$$\overline{\alpha}_{\rm eff} = \overline{\theta}_{\rm eff}\left(\overline{n}_{\rm s}-n_1\right) = \overline{\theta}_{\rm eff}\left(\left(1-\overline{x}_{\rm lip}\right)\overline{n}_{\rm p}+\overline{x}_{\rm lip}\overline{n}_{\rm lip}-n_1\right). \tag{S20}$$

Hence, we have

$$\frac{\partial\overline{\alpha}_{\rm eff}}{\partial\overline{x}_{\rm lip}} = \overline{\theta}_{\rm eff}\left(\overline{n}_{\rm lip}-\overline{n}_{\rm p}\right)+\frac{\partial\overline{\theta}_{\rm eff}}{\partial\overline{x}_{\rm lip}}\frac{\overline{\alpha}_{\rm eff}}{\overline{\theta}_{\rm eff}}. \tag{S21}$$

IX. REFRACTIVE INDEX VALUES OF LARVAL ZEBRAFISH TRUNK TISSUE AT 96 PF FROM [15]

The RI values of larval zebrafish trunk tissue at 96 hpf employed in this study are not explicitly stated in [15], hence we list them here.

TABLE S4: Refractive indices n and according standard deviations Δn of the trunk tissue of $N = 20$ zebrafish larvae at 96 hpf from [15].

N	1	2	3	4	5	6	7	8	9	10
n	1.36501	1.36524	1.36691	1.36679	1.36556	1.36251	1.36319	1.36098	1.36112	1.36342
Δn	0.00225	0.00369	0.00307	0.00315	0.00268	0.00151	0.00122	0.00210	0.00241	0.00229
N	11	12	13	14	15	16	17	18	19	20
n	1.36763	1.36604	1.36673	1.36670	1.36550	1.36640	1.36633	1.36632	1.36662	1.36693
Δn	0.00182	0.00205	0.00233	0.00211	0.00219	0.00238	0.00220	0.00172	0.00130	0.00238

X. ESTIMATIONS OF $x_{\rm lip}$ AND φ_1 OF LARVAL ZEBRAFISH AT 96 HPF

Based on the measurements of [14], where they determined the wet mass $m_{\rm tot}$, dry mass $m_{\rm dry}$, protein mass $m_{\rm p}$ and the lipid mass $m_{\rm lip}$ of larval zebrafish at 96 hpf, we define the relative lipid mass fraction $y_{\rm lip} \equiv m_{\rm lip}/(m_{\rm lip}+m_{\rm p})$ and consider the ratio

$$\frac{y_{\rm lip}}{y_{\rm p}} = \frac{m_{\rm lip}}{m_{\rm p}} = \frac{\theta^{\rm eff}_{\rm p}x_{\rm lip}}{\theta^{\rm eff}_{\rm lip}(1-x_{\rm lip})}, \tag{S22}$$

from which we obtain

$$x_{\rm lip} = \frac{m_{\rm lip}\theta^{\rm eff}_{\rm lip}}{m_{\rm lip}\theta^{\rm eff}_{\rm lip}+m_{\rm p}\theta^{\rm eff}_{\rm p}}, \tag{S23}$$

and analogously

$$\varphi_1 = \frac{(m_{\rm tot}-m_{\rm dry})}{m_{\rm tot}+m_{\rm dry}\left(\rho_1\theta^{\rm eff}_{\rm dry}-1\right)}. \tag{S24}$$

Based on this dry mass composition we estimate the distributions of $\theta^{\rm eff}_{\rm p}$ and $\theta^{\rm eff}_{\rm lip}$ by running the MC simulation of the extended mixture model with $x_{\rm lip} = 0$ and $x_{\rm lip} = 1$, respectively. We may now obtain the distribution of $x_{\rm lip}$ by a MC sampling approach, considering the distributions of all individual parameters. Next, we compute $\theta^{\rm eff}_{\rm dry}$ by repeating the procedure described above for $x_{\rm lip}$ following the distribution determined previously. With that we obtain the distribution of φ_1.

XI. RELATIVE UNCERTAINTY OF THE RI FOR DIFFERENT MATERIAL PROPERTIES

The dependence of the relative RI deviation on the deviation of the relative lipid volume fraction Δx_{lip} for different deviations of the water volume fraction $\Delta\varphi_1$, as pointed out in the main text, can be qualitatively understood with Fig. S1.

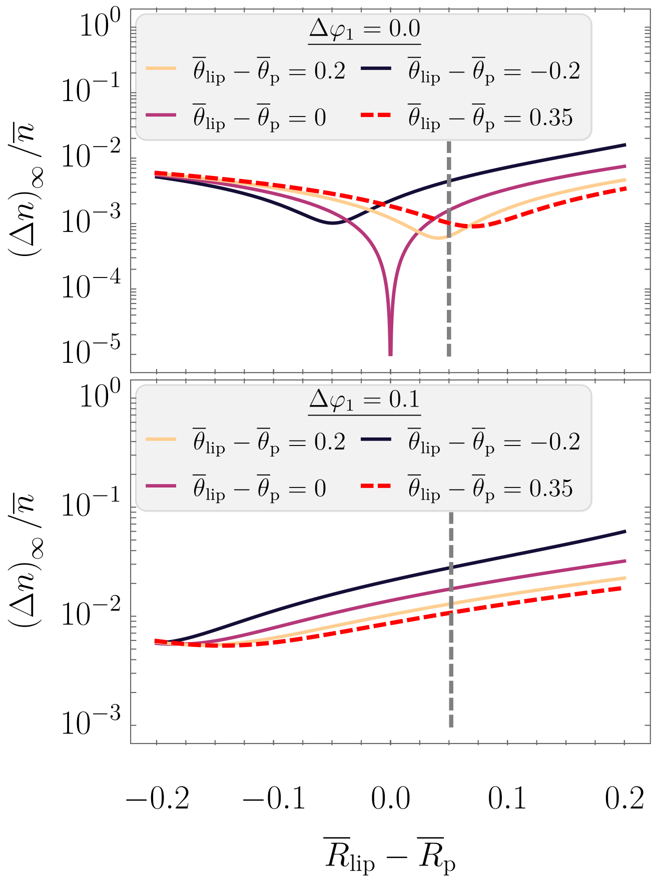

FIG. S1: Asymptotic relative deviation of the RI $(\Delta n)_\infty/\overline{n}$ for different potential differences of lipid and protein PSVs $\overline{\theta}_{\text{lip}} - \overline{\theta}_{\text{p}}$ in dependence of the difference of lipid and protein refractions per gram $\overline{R}_{\text{lip}} - \overline{R}_{\text{p}}$ for the cases of a non-fluctuating water volume fraction $\Delta\varphi_1 = 0$ (top) and a fluctuating water volume fraction $\Delta\varphi_1 = 0.1$ (bottom).

XII. STRATEGIES FOR ESTIMATING THE MD FOR CERTAIN EXPERIMENTAL PARADIGMS

In Fig. S2, we outline possible strategies for estimating the MD, given certain experimental insights. In the following, we use the same abbreviations as in the main text, namely,

- RI = refractive index,
- (S)RS = (stimulated) Raman spectroscopy,
- MS = mass spectrometetry.

We want to point out that the estimation process is heavily dependent on identifying relevant solute constituents of the sample, as well as their PSVs, RIs and/or refractions per gram. For the latter, secondary data bases, such as `ChemSpider` [8], are invaluable. Furthermore, although RI measurements are not necessary to predict $\rho(\delta n)$, as outlined in the main text, comparing predicted and measured RI distributions gives necessary insight on the validity of the prediction.

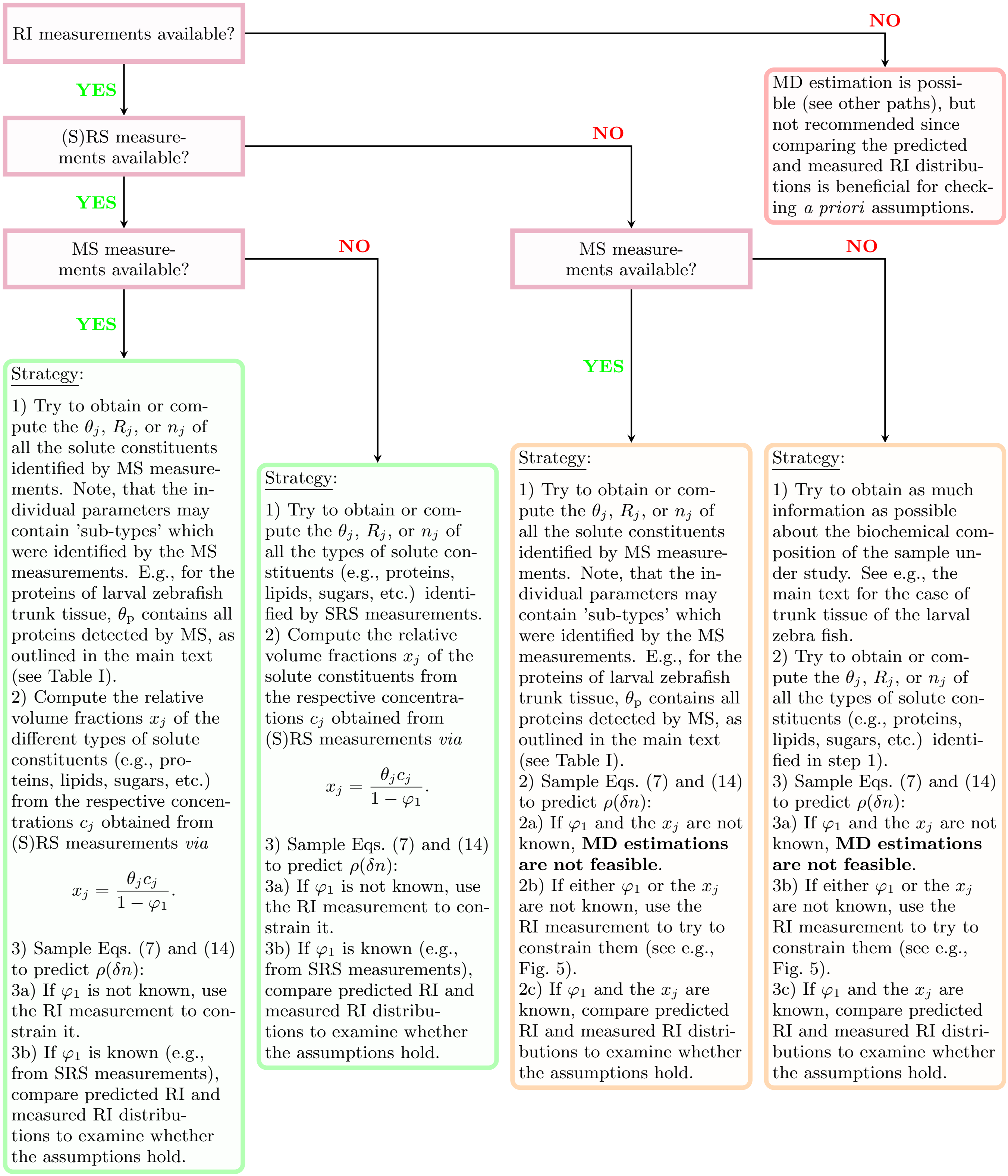

FIG. S2: Flowchart of mass density estimation approaches outlined in this study, based on different experimental paradigms. The abbreviations and symbols are as defined in the main text.

SUPPORTING REFERENCES

[1] A. Zamyatnin, Progress in Biophysics and Molecular Biology **24**, 107 (1972).
[2] H. Zhao, P. H. Brown, and P. Schuck, Biophysical Journal **100**, 2309 (2011).
[3] S. J. PERKINS, European Journal of Biochemistry **157**, 169 (1986).
[4] D. Khago, J. C. Bierma, K. W. Roskamp, N. Kozlyuk, and R. W. Martin, Journal of Physics: Condensed Matter **30**, 435101 (2018).
[5] Wolfram Research, Inc., Wolfram|Alpha Knowledgebase, Champaign, IL (2022). (2022).
[6] S. Kim, J. Chen, T. Cheng, A. Gindulyte, J. He, S. He, Q. Li, B. A. Shoemaker, P. A. Thiessen, B. Yu, L. Zaslavsky, J. Zhang, and E. E. Bolton, Nucleic Acids Research **51**, D1373 (2022).
[7] T. L. McMeekin, M. Wilensky, and M. L. Groves, Biochemical and Biophysical Research Communications **7**, 151 (1962).
[8] ChemSpider

- Triolein, CSID:4593733, http://www.chemspider.com/Chemical-Structure.4593733.html
- Palmitic acid , CSID:960, http://www.chemspider.com/Chemical-Structure.960.html
- Oleic acid, CSID:393217, http://www.chemspider.com/Chemical-Structure.393217.html
- Docosahexaenoic acid, CSID:393183, http://www.chemspider.com/Chemical-Structure.393183.html
- Stearic acid, CSID:5091, http://www.chemspider.com/Chemical-Structure.5091.html

(Accessed Feb 2, 2023).
[9] T. U. Consortium, Nucleic Acids Research **51**, D523 (2022).
[10] P. Walstra, J. T. M. Wouters, and T. J. Geurts, *Dairy Science and Technology* (CRC Press, 2005).
[11] Merck

- Soybean oil, 47122, https://www.sigmaaldrich.com/DE/de/product/supelco/47122
- Glycerol, 104057, https://www.merckmillipore.com/DE/de/product/Glycerol,MDA_CHEM-104057?ReferrerURL=https%3A%2F%2Fwww.google.com%2F

(Accessed Aug 31, 2023).
[12] N. Bansal, T. Truong, and B. Bhandari, Dairy Science & Technology **96**, 861 (2017).
[13] Oxford Lab Fine Chem LLP

- Soybean lecithin, https://www.oxfordlabchem.com/msds/(S-08441)SOYA%20LECITHIN%2030%20Extra%20Pure.pdf

(Accessed Aug 31, 2023).
[14] Y. Long, L. Li, Q. Li, X. He, and Z. Cui, PLOS ONE **7**, 1 (2012).
[15] J. Kolb, V. Tsata, N. John, K. Kim, C. Möckel, G. Rosso, V. Kurbel, A. Parmar, G. Sharma, K. Karandasheva, S. Abuhattum, O. Lyraki, T. Beck, P. Müller, R. Schlüßler, R. Frischknecht, A. Wehner, N. Krombholz, B. Steigenberger, D. Beis, A. Takeoka, I. Blümcke, S. Möllmert, K. Singh, J. Guck, K. Kobow, and D. Wehner, Nature Communications **14**, 6814 (2023).
[16] N. Hachicho, S. Reithel, A. Miltner, H. J. Heipieper, E. Küster, and T. Luckenbach, PLOS ONE **10**, 1 (2015).
[17] F. Pretorius, W. W. Focke, R. Androsch, and E. du Toit, Journal of Molecular Liquids **332**, 115893 (2021).
[18] P. Brocos, A. Piñeiro, R. Bravo, and A. Amigo, Phys. Chem. Chem. Phys. **5**, 550 (2003).
