# Supplementary material for: Estimation of the mass density of biological matter from refractive index measurements
Source: Biophys Rep (N Y). 2024 Apr 24;4(2):100156. doi: 10.1016/j.bpr.2024.100156 (PMC11090064; doi:10.1016/j.bpr.2024.100156)
Supplement: Document S2. Article plus supporting material [file mmc2.pdf]

Biophysical Reports

ARTICLE

Estimation of the mass density of biological matter from refractive index measurements

Conrad Möckel,[1,2,3] Timon Beck,[1,2] Sara Kaliman,[1,2] Shada Abuhattum,[1,2] Kyoohyun Kim,[1,2] Julia Kolb,[1,2,3] Daniel Wehner,[1,2] Vasily Zaburdaev,[2,3] and Jochen Guck[1,2,3,*]

[1]Max Planck Institute for the Science of Light, Erlangen, Germany; [2]Max-Planck-Zentrum für Physik und Medizin, Erlangen, Germany; and [3]Friedrich-Alexander-Universität Erlangen-Nürnberg, Erlangen, Germany

ABSTRACT The quantification of physical properties of biological matter gives rise to novel ways of understanding functional mechanisms. One of the basic biophysical properties is the mass density (MD). It affects the dynamics in sub-cellular compartments and plays a major role in defining the opto-acoustical properties of cells and tissues. As such, the MD can be connected to the refractive index (RI) via the well known Lorentz-Lorenz relation, which takes into account the polarizability of matter. However, computing the MD based on RI measurements poses a challenge, as it requires detailed knowledge of the biochemical composition of the sample. Here we propose a methodology on how to account for assumptions about the biochemical composition of the sample and respective RI measurements. To this aim, we employ the Biot mixing rule of RIs alongside the assumption of volume additivity to find an approximate relation of MD and RI. We use Monte-Carlo simulations and Gaussian propagation of uncertainty to obtain approximate analytical solutions for the respective uncertainties of MD and RI. We validate this approach by applying it to a set of well-characterized complex mixtures given by bovine milk and intralipid emulsion and employ it to estimate the MD of living zebrafish (*Danio rerio*) larvae trunk tissue. Our results illustrate the importance of implementing this methodology not only for MD estimations but for many other related biophysical problems, such as mechanical measurements using Brillouin microscopy and transient optical coherence elastography.

WHY IT MATTERS Mass density is a fundamental property of living matter. It is of central importance for understanding inherent functional mechanisms by quantifying (statistical) processes and associated mechanical properties. Currently, there is no experimental paradigm available to measure in vivo mass density directly. Indirectly, the mass density can be inferred from refractive index measurements. However, we lack a robust framework that is capable of accounting for the complex chemical composition of biological matter. Our manuscript directly addresses this gap by establishing an experimentally validated, cohesive theoretical framework on how mass density of biological matter can be estimated using (in vivo) refractive index measurements.

INTRODUCTION

Quantifying the physical properties of biological matter has become increasingly important over recent decades. The notion that biological function of cells and tissues is affected by their physical phenotype and vice versa has been validated in many experimental studies (see, e.g., (1–3)). A fundamental property of matter, including living matter, is the mass density (MD) (4), which not only is associated with buoyancy, crowdedness (5), biomolecular condensation (6), and inherent dynamical processes of the sample of interest (7,8) but also plays a major role in elastography, particularly Brillouin microscopy (9–12) and transient optical coherence elastography (see, e.g., (13,14)). However, measuring the in vivo MD distribution in a direct manner poses a challenge that has not been resolved so far. One way of inferring the in vivo MD of a sample is to measure the refractive index (RI) via microscopy techniques, such as quantitative phase imaging (QPI), particularly optical diffraction tomography (ODT) (15,16). The Lorentz-Lorenz relation then connects the RI with the MD if the molar refractivity and partial specific volume (PSV) of the dry mass composition and the solvent content are known. This knowledge, however, is not trivially obtainable. A customary assumption regarding

Submitted January 12, 2024, and accepted for publication April 19, 2024.

*Correspondence: jochen.guck@mpl.mpg.de

Editor: Jorg Enderlein.

https://doi.org/10.1016/j.bpr.2024.100156

© 2024 The Author(s).

This is an open access article under the CC BY license (http://creativecommons.org/licenses/by/4.0/).

biological matter is that the dry mass composition is given by proteins only (16–19) and that the solvent content is then indirectly constrained by the measured RI. Although this approximation holds for binary solutions, it cannot be directly extended to samples with a complex dry mass composition.

In the context of cells and tissues, the dry mass composition may be thought of as a mixture of (phase-separated) proteins, lipids, sugars, etc. (20). By employing, e.g., mass spectrometry (MS) and/or (stimulated) Raman spectroscopy ((S)RS), individual components and their respective concentrations in the sample can be identified (21–23). Additionally, correlative fluorescence information could be employed to segment RI maps acquired by ODT (12,24). However, these experimental modalities might not be available or applicable for certain samples, which creates a degree of ignorance about the dry mass composition that should be considered in the inference process of obtaining an MD estimate. Another closely related aspect is the robust estimation of the uncertainty of the MD. Considering the previously mentioned degree of ignorance, these uncertainties are clearly not only of statistical but also of a systematic nature. Further, even if universal knowledge about the true distributions of the molar refractivity and PSV were available, to estimate the uncertainty of the MD adequately, the uncertainties of the individual parameters should be propagated.

Here, we present a robust methodology for estimating the uncertainties of the MD and the correlative RI by employing Monte-Carlo (MC) simulations. We provide analytical approximations for both the MD and RI distributions in dependence of the dry mass composition and the solvent content, employing Gaussian propagation of uncertainty.

To this end, we first motivate a simple mixture model to estimate the relationship between the MD and the RI from two material constants, namely the RI increment α and the PSV θ. We then extend the model toward unimodal distributions of α and θ, for which previously only precise values were assumed.

The distributions of the RI increment and PSV are remarkably narrow when only considering proteins in the mixture (15,25), resulting in sharp distributions of RI and MD. However, taking a second type of molecule, such as lipids or sugars, into account adds an additional complexity to the MD estimations since their values of RI increment and PSV differ drastically from those of proteins. Therefore, we derive an effective description of the system based on weighted mixture distributions. This allows for a correlative prediction of the RI and the MD, accounting, e.g., for the lipid and water content of the sample and fluctuations in both quantities. We then apply this approach to 1) bovine milk, a well-characterized mixture of water, proteins, and lipids; and 2) 20% intralipid emulsion (IL), which mainly consists of water and soybean oil. Comparing the measured values of the MD and RI acquired by pycnometry and Abbe refractometry, respectively, with our theoretical estimates, we find both to be in good agreement with each other.

After demonstrating the applicability of our model on bovine milk and IL, we explore the MD distribution of larval zebrafish trunk (comprising major tissue, including muscle and spinal cord) employing the recent RI and MS measurements of (21). Although the accurate determination of the biochemical composition of the tissue poses a challenge, beset by numerous (crude) uncertainties, the measured RI distribution and our prediction coincide within one standard deviation (SD). This purely optical and computational approach shows how the MD can be estimated in complex in vivo specimens, enabling a more profound interpretation of mechanical measurements.

MATERIALS AND METHODS

Sample preparation

The skim-milk powder (SM) was dissolved in distilled water while carefully stirring the solution to avoid foaming. The solution was then left on a tilt/roller mixer for approximately 30 min. For the IL, according amounts of water were added to the emulsion and the solution was left on a tilt/roller mixer for approximately 30 min as well. All samples were freshly prepared before the measurements were performed.

Abbe refractometry and pycnometry

For measuring the RI of a liquid sample, 100 μL of the sample were loaded into an Abbe refractometer (KERN ORT 1RS) and a commercially available flashlight light-emitting diode (LED) was employed as illumination source.

To determine the density of a liquid sample, a pycnometer (Blaubrand 43305) was employed. First, the volume of the pycnometer was determined by employing distilled water as a calibration sample ($N = 10$ technical repetitions) as

$$V_{pyc} = \frac{m_w - m_{pyc}}{\rho_{w,lit.}} = (4.9455 \pm 0.0017)\ \mathrm{mL}, \quad (1)$$

where m_w is the mass of the pycnometer filled with water, m_{pyc} is the mass of the empty pycnometer, and $\rho_{w,lit.} = 0.997$ g/mL is the literature value of the density of water at 23°C. The respective masses were measured using a high-precision lab scale (Ohaus Pioneer PX124). The density of the liquid sample under study was then computed by

$$\rho = \frac{m - m_{pyc}}{V_{pyc}}. \quad (2)$$

Measurement uncertainties

The systematic uncertainties of the respective measurement devices under use were taken from the manuals and considered in all calculations together with the statistical uncertainties as

$$\Delta z = \sqrt{\Delta z_{\mathrm{sys}}^2 + \Delta z_{\mathrm{stat}}^2}, \quad (3)$$

where z is an arbitrary observable.

Data analysis

All data analysis, plotting, and simulations were performed using custom scripts in Wolfram Research, Mathematica, Version 12.2 (26).

BACKGROUND

A binary mixture model for MD estimations

In the following, we consider a mixture of a solute (denoted by index 2) in a solvent (denoted by index 1) with total mass m and volume v. The MD $\rho = m/v$ of the mixture can be expressed in terms of the MDs ρ_i and volume fractions $\varphi_i = c_i/\rho_i$ of its components as

$$\rho = \varphi_1\rho_1 + \varphi_2\rho_2, \quad (4)$$

where the $c_i = m_i/v$ are the concentrations of the respective constituents and we denote the total volume of the mixture by $v = v_1 + m_2\vartheta$. Here, ϑ is the so-called apparent specific volume (ASV) of the solute, which describes the volume per gram of the solute in solution. As such, the ASV may be dependent on the mass of the solute m_2, since it accounts for solute-solute interactions under constant temperature T, pressure p, and solvent mass m_1. The change of the total volume of the solution v with respect to a change in the mass of the solute is then characterized by the PSV via

$$\theta \equiv \left(\frac{\partial v}{\partial m_2}\right)_{T,p,m_1} = \vartheta + m_2\left(\frac{\partial \vartheta}{\partial m_2}\right)_{T,p,m_1}, \quad (5)$$

as motivated in (27). For the sake of simplicity, for all the following considerations, we employ the concept of volume additivity, for which it is straightforward to show that $\theta = \vartheta = 1/\rho_2$ (see Eq. S3 in the Supporting Material).

By assuming volume additivity $[(1 - \varphi_2) = \varphi_1]$, we may express Eq. 4 in terms of the solvent MD, PSV, and solute concentration as

$$\rho = \rho_1(1 - c_2\theta) + c_2. \quad (6)$$

In the next step, we connect the RI of the solution to the solute concentration c_2. For that purpose we employ the phenomenological mixing rule

$$n = \varphi_1 n_1 + \varphi_2 n_2, \quad (7)$$

where we assumed that the RI follows the same mixture rule as the MD, given in Eq. (4). In the following, we refer to Eq. 7 as Biot equation or mixing rule (28), which is commonly employed in the context of QPI and ODT (15). Note that, in a typical ODT experiment, we determine the RI contrast $\delta n \equiv n - n_1$, which has to fulfill a nonnegativity constraint (i.e., $\delta n \geq 0$). Evaluating Eq. 7 while assuming volume additivity, we arrive at

$$\frac{\delta n}{c_2} = \theta(n_2 - n_1) = \alpha, \quad (8)$$

where we identify the customarily designated RI increment $\alpha \equiv \partial n/\partial c_2$.

Finally, by replacing c_2 in Eq. 6 with the expression given in Eq. 8, we obtain an estimate of the solution MD in dependence of the RI as

$$\rho = \frac{\delta n}{\alpha} + \rho_1\left(1 - \theta\frac{\delta n}{\alpha}\right), \quad (9)$$

which has been employed in (19), or stated equivalently in (29). Eq. 9 is the basis of all the following considerations and will be employed frequently throughout this study. We note that the relationship between MD and RI, given in Eq. 9 is linear and the slope $\partial\rho/\partial n = (1 - \rho_1\theta)/\alpha$ is only determined by the PSV θ and the RI increment α of the solute. In biological matter, the solvent is assumed to be water, for which the MD ρ_1 and RI n_1 are accurately known.

Experimentally, the PSV of a solute is determined by measuring the density of the solution ρ in dependence of the solute concentration c_2 (27,30). A straightforward and common approach to obtain the MD of a liquid is given by pycnometry, where the mass of a precisely fixed volume is measured. The MD is then simply given by the mass-to-volume ratio. Further methods to measure MD are reviewed in (27) and references therein. Similarly, the RI increment is customarily determined by measuring the RI of the solution in dependence of the solute concentration (see Eq. 8), e.g., by employing an Abbe refractometer.

To our knowledge, the justification for or against the assumption of volume additivity in complex biological matter is yet to be given (experimentally). We may interpret Eq. 5 in first-order approximation as $\theta \approx \vartheta \pm \Delta\vartheta$, where any potential deviation from the volume additivity $\Delta\vartheta$ could be treated as an additional factor that will also contribute to the uncertainty of the RI and MD estimates.

As pointed out earlier, Eq. 8 is commonly used in the context of QPI and ODT to determine solute densities ($\rho_2 = 1/\theta$) and subsequently solute masses of complex biological samples from tomographic RI measurements (15). To that aim the RI increments of various proteins are customarily employed (17, 18, 31–35). Although this approach holds for the assumption that biological matter consists of protein and water, we will show later that it is not sufficient for

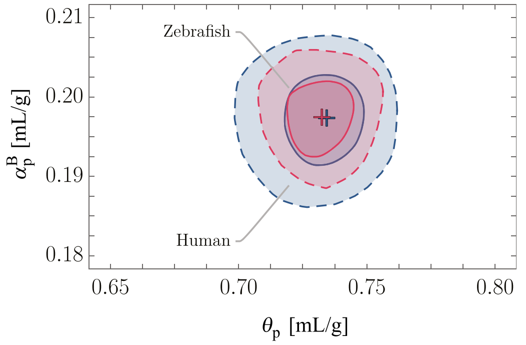

FIGURE 1 Correlative distributions $\mathcal{P}(\alpha_p^B, \theta_p)$ of the RI increment α_p^B and PSV θ_p for the human proteome (*blue*; 82,127 proteins included) and the zebrafish proteome (*red*; 46,517 proteins included). The solid and dashed lines indicate the 68% and 95% confidence contours, respectively.

inferences about complex solute composition with more than one constituent. In brief, this is because there exists no universal relationship $\alpha \sim \theta$ (i.e., $n \sim \rho$) for different materials (see, e.g., (36); Fig. 1).

Complex mixtures

In the context of biological matter, one may think of the solute as a complex composition of many constituents, which makes the experimental determination of both PSV and RI increment for all components and their combinations practically impossible; the human proteome alone consists of $\sim 10^4$ proteins (20,37). To resolve this problem, at least partially, without accounting for volume inadditivities, a method of determining the correlative distributions $\mathcal{P}(\alpha, \theta)$ of the proteomes of different organisms was introduced and experimentally validated for two amino acid sequences in (25). The authors computed the molar mass averages of the residue refractivity per gram and according PSV θ of proteins based on their respective amino acid sequence. The refractivity per gram R_i of a molecule i is proportional to its polarizability $\widehat{\alpha}_i$ and molar mass M_i as $R_i \sim \widehat{\alpha}_i / M_i$. The refractivity per gram can then be connected to the RI and the MD of the solute via the Lorentz-Lorenz (or Clausius-Mossotti) relation (see, e.g., (38)) as

$$R_i = \frac{1}{\rho_i} \frac{n_i^2 - 1}{n_i^2 + 2}. \tag{10}$$

We note that taking the mass averages over the amino acid residue refractivities per gram in combination with the Lorentz-Lorenz relation Eq. 10 leads to the Lorentz-Lorenz mixing rule of RIs Eq. S8.

However, assuming dilute solutions ($n \to n_1$) and volume additivity, the authors of (25) computed the RI increment for each protein (denoted by the index p) by employing the Wiener mixing rule of RIs (see, e.g., (28)) and Eq. 10 to compute the RI of each protein n_p from the mass averaged refractions per gram and PSVs of the respective amino acid sequences, as described earlier. Repeating this procedure for all proteins presumed to be abundant in the different organisms under study, they obtained the bivariate distribution of RI increment and PSV. Zhao et al. (25) then fitted normal distributions to the univariate histograms to obtain the means and SDs for different organisms, of which we depict two in Table 1. We repeated the computations presented in (25) (see Supporting Material) for the updated human and zebrafish proteomes obtained from (37). Besides computing the RI increment from the Wiener relation for dilute solutions, we also employed the Biot Eq. 7 in combination with the volume additivity assumption to obtain an expression of the RI increment as given in Eq. 8.

Additionally, different from (25), we employed the consensus averages for the amino acid residue molecular volumes of (30) instead of the ones of Cohn and Edsall (39). The full list of parameters employed here is given in Table S1. The resulting bivariate distributions $\mathcal{P}(\alpha, \theta)$ are given in Fig. 1. The corresponding mean values and SDs of the fits of the univariate histograms with a normal distribution are given in Table 1. Evidently, the PSV and RI increment values obtained here, using the Wiener equation for dilute solutions, coincide well with the values from (25). We note that the RI increments obtained using Eq. 8 are systematically higher than the ones obtained from the Wiener equation for dilute solutions.

Although it is not clear why the calculations proposed in Zhao et al. (25) result in distributions $\mathcal{P}(\alpha, \theta)$ that resemble uncorrelated bivariate normal distributions for the proteomes of different species, they facilitate the idea of taking the whole distribution $\mathcal{P}(\alpha, \theta)$ into account when estimating the MD via Eq. 9. Consistently, to obtain a reliable estimate of the MD distribution, a precise characterization of the solute composition is needed.

We note that, although relations similar to Eq. 9 can be found for different (phenomenological) RI mixing rules (see, e.g., (40–42)), or effective (nonlocal) RI descriptions based on light scattering theory (see, e.g., (32,43–46)), we expect limited additional qualitative insight compared to the Biot Eq. 7; ultimately, for each theory one may compute the RI increment $\alpha(c_2)$, (numerically) solve the expression for the solute concentration c_2, and substitute it in Eq. 6 to find a relationship $\rho(\delta n)$ for the given RI mixture rule. To illustrate this procedure, we derive such an expression for the Lorentz-Lorenz mixing rule in the Supporting Material (see Eq. S11).

In the following, we construct and employ a theoretical framework in which the information of the solute

TABLE 1 Mean and SD of the RI increments α_p^i and PSVs θ_p Distributions Based on Amino Acid Sequences of the Proteome of the Human and Zebrafish, and the Trunk Tissue of the Larval Zebrafish at 96 hpf

	$\overline{\alpha}_p^W$ in mL/g	$\Delta\alpha_p^W$ in mL/g	$\overline{\alpha}_p^B$ in mL/g	$\Delta\alpha_p^B$ in mL/g	$\overline{\theta}_p$ in mL/g	$\Delta\theta_p$ in mL/g
Human						
Proteome[a]	0.1899	0.0030	N/A	N/A	0.735	0.010
Proteome[b]	0.188	0.004	0.197	0.004	0.734	0.012
Zebrafish						
Proteome[a]	0.1904	0.0030	N/A	N/A	0.735	0.010
Proteome[b]	0.1887	0.0031	0.1974	0.0034	0.732	0.010
Trunk tissue[b]	0.1883	0.0029	0.1971	0.0033	0.734	0.009

The calculation of α_p^B employed the Biot equation given in Eq. 7, whereas α_p^W was derived from the dilute limit of the Wiener mixing rule of refractive indices (28).
[a]Zhao et al. (25)
[b]This work

composition is incorporated into the prediction of the MD. For that purpose, we employ the Biot Eq. 7 for the majority of our further considerations and denote $\alpha = \alpha^B$.

RESULTS

Extension of the binary mixture model

When dealing with biological matter, the complexity of the solute composition should be taken into account to obtain reliable estimates of the MD. To that aim, we first extend Eq. 9 to the case of different solute constituents (e.g., proteins of the human proteome, lipids, and sugars) being dissolved in a solvent with corresponding RI n_1 and MD ρ_1.

We describe this problem by discretizing the total sample volume into N_v voxels with volumes v_v

$$v = N_v v_v. \tag{11}$$

Furthermore, we discretize the voxels into N_0 "voxelinos" with volumes v_0 as

$$v_v = N_0 v_0. \tag{12}$$

The motivation to divide a voxel into N_0 voxelinos is to obtain small, yet macroscopic, volume fractions with a constant volume v_0 that contain one and only one solution constituent. Hence, each voxelino can be either a solvent or solute voxelino and is inherently characterized by its respective PSV θ_i and refraction per gram R_i (i.e., its MD and RI). The number of solvent voxelinos in the voxel, N_1, is given by $N_1 = N_0 - N_s$, where N_s is the number of solute voxelinos (see Fig. 2 for a visual interpretation).

Accordingly, the solvent volume fraction of a voxel is given by

$$\varphi_1 \equiv 1 - \frac{N_s}{N_0}. \tag{13}$$

Accounting for multiple types of solute molecules (e.g., proteins, lipids, sugars) being present in the solution, we chose the PSVs and refractions per gram of the solute voxelinos θ_{i+1} and R_{i+1} to be random values, drawn from a weighted mixture distribution

$$\mathcal{P}_{mix}(R, \theta) = \sum_j x_j \mathcal{P}_j(R, \theta), \tag{14}$$

where j denotes the solute constituents. Further, the $\mathcal{P}_j(R, \theta)$ represent the bivariate probability distributions of the refraction per gram and PSV of the respective constituents, and the associated weights x_j are given by the relative volume fractions as $x_j \equiv N_j / N_s$. We note that Eq. 14 should be seen as a way of denoting that N_j out of N_s solute voxelinos of constituent j are present in a voxel. Consequently, N_j solute voxelinos have the RI n_j and MD ρ_j and we have that $N_s = \sum_j N_j$, i.e., $\sum_j x_j = 1$.

Employing Eqs. 6 and 7, we readily obtain the relation between the MD ρ and the RI n of one voxel

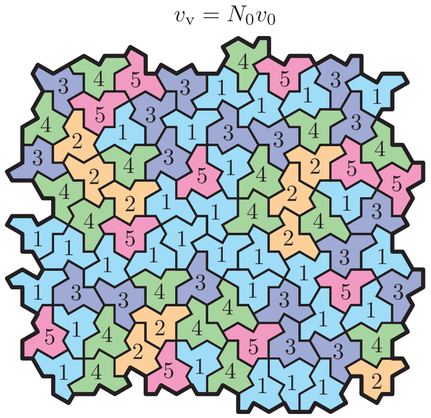

FIGURE 2 2D visual interpretation of a voxel with volume $v_v = N_0 v_0$ consisting of N_0 voxelinos with volumes v_0. Each number stands for one type of voxelino; 1 corresponds to the solvent voxelinos and 2 – 5 correspond to the solute voxelinos, e.g., four different proteins. Each voxelino is characterized by its PSV θ_i, refraction per gram R_i and has a corresponding mass of $m_i = v_0/\theta_i$. This illustrative depiction is based on (47).

$$\rho = \sum_{i=1}^{N_s} c_{i+1} + \rho_1 \left(1 - \sum_{i=1}^{N_s} \theta_{i+1} c_{i+1}\right) \equiv \frac{\delta n}{\alpha_{\text{eff}}} + \rho_1 \left(1 - \theta_{\text{eff}} \frac{\delta n}{\alpha_{\text{eff}}}\right), \tag{15}$$

where we defined the effective RI increment α_{eff} and PSV θ_{eff}. The RI of the solution n is then given by the Biot Eq. 7, where the RIs of the individual solute voxelinos n_{i+1} could be known directly from RI measurements, or may be computed by employing the Lorentz-Lorenz relation, given in Eq. 10.

It can be shown (see Eqs. S17 and S18) that the effective parameters may be expressed by the mass averages of the respective parameters of the solute voxelinos as

$$\theta_{\text{eff}} = \sum_{i=1}^{N_s} y_{i+1} \theta_{i+1}, \quad \alpha_{\text{eff}} = \sum_{i=1}^{N_s} y_{i+1} \alpha_{i+1}, \tag{16}$$

where we denoted the relative mass fraction of a solute voxelino by $y_i \equiv m_i / m_s$, with m_s being the total solute mass. Hence, the effective parameters absorb the complexity of the mixture, whereas the functional relationship of Eq. 6 is obeyed. With this at our disposal, we are able to compute the MD ρ and the corresponding RI n of a voxel, given a distribution $\mathcal{P}_{\text{mix}}(R, \theta)$ and a solvent volume fraction of the voxels φ_1 (i.e., the ratio N_s/N_0). Repeating this procedure for N_v voxels provides a map of RI values in resemblance of an ODT measurement alongside the corresponding MDs.

Since the framework introduced above is strongly dependent on a priori assumptions of the individual model parameters (φ_1, x_j, α_j, θ_j) for different complex mixtures, in the next step, we discuss the impact of these assumptions on the uncertainties of the RI and MD predictions.

Uncertainty quantification of the extended model

Given access to experimental data (i.e., ODT tomograms of a sample of interest), we may compare not only the mean values of the measured and predicted RI distributions but also their widths. This in turn enables a more reliable estimate of the MD distribution. Hence, in the next step, we study the dependence of the uncertainty of the MD estimate ρ, defined in Eq. 15, and the RI n on the sample properties, namely, the solvent volume fraction φ_1, the effective RI increment α_{eff}, the effective PSV θ_{eff}, and their respective associated uncertainties.

To that aim, we investigated the impact of the presence of a second type of macro molecule in the mixture of proteins and water. Since lipids make up for about 13% of the solute mass fraction in mammalian cells (20) and typically exhibit an MD lower than water, they merit detailed scrutiny. In the following, we assume that the lipids are present in the form of lipid droplets and form an emulsion in the water + protein phase. For the sake of simplicity, we further assume that the lipid droplets consist only of the neutral lipid triolein, neglecting sterol esters, triacylglycerols, and phospholipids (48–50). The corresponding values of the RI, refraction per gram, PSV, and RI increment of the two types of solute molecules under investigation are given in Table S2.

Throughout this study, we assume that both RI and MD of the solvent are precisely known. We further point out that we implement the values given in Table S2 in our calculations as follows: if a quantity is stated as mean ± SD, we account for it as normally distributed with respective mean and SD. For the cases where we could not estimate an uncertainty, we assume the quantity to be delta distributed.

Effective RI increment and PSV

Examining the definitions of α_{eff} and θ_{eff}, given in Eq. 16, we observe a dependence of both quantities on the number of solute voxelinos per voxel N_s, given as the upper limit of the sum. To obtain an intuition about the implications on the respective uncertainties, we first consider the case of proteins dissolved in water, which may be approximated by uncorrelated normal distributions $\mathcal{N}(\mu, \sigma)$ of the RI increment and PSV, as shown in Fig. 1

$$\alpha^{\text{N}} \sim \mathcal{N}(\overline{\alpha}, \Delta\alpha), \quad \theta^{\text{N}} \sim \mathcal{N}(\overline{\theta}, \Delta\theta), \tag{17}$$

with respective mean values and SDs (see, e.g., Table 1). Applying Gaussian propagation of uncertainty to Eq. 16, we have

$$\Delta\theta_{\text{eff}} = \frac{\Delta\theta}{\sqrt{N_s}}, \quad \Delta\alpha_{\text{eff}} = \frac{\Delta\alpha}{\sqrt{N_s}}, \tag{18}$$

in which the relative mass fraction of each solute voxelino is given by $y_{i+1} = 1/N_s$. The result of Eq. 18 is in concordance with the central limit theorem. In other words, the SD of the effective distributions corresponds to the SE of the mean of the initial distributions, given that the voxel contains N_s protein voxelinos. Thus, considering that, in biological matter, the number of voxelinos per voxel would be typically larger than $\sim 10^8$ for experimentally accessible voxel

sizes in the order of 1 μm^3, the deviations of the effective RI increment and PSV for the case of proteins dissolved in water are negligibly small.

Next, we study the impact of lipids in the protein + water mixture. In this scenario, Eq. 14 takes the form

$$\mathcal{P}_{\mathrm{p+lip}}(R,\theta) = (1 - x_{\mathrm{lip}})\mathcal{P}_{\mathrm{p}}(R,\theta) + x_{\mathrm{lip}}\mathcal{P}_{\mathrm{lip}}(R,\theta). \quad (19)$$

Assuming that $\mathcal{P}_{\mathrm{p}}$ and $\mathcal{P}_{\mathrm{lip}}$ follow uncorrelated bivariate normal distributions, using Eq. 18, we find that the distributions of the effective PSV and RI increment follow normal distributions as

$$\begin{aligned} \theta_{\mathrm{eff}} &\sim \mathcal{N}\left(\overline{\theta}_{\mathrm{eff}}, \Delta\theta^0_{\mathrm{eff}} \Big/ \sqrt{N_{\mathrm{s}}}\right), \\ \alpha_{\mathrm{eff}} &\sim \mathcal{N}\left(\overline{\alpha}_{\mathrm{eff}}, \Delta\alpha^0_{\mathrm{eff}} \Big/ \sqrt{N_{\mathrm{s}}}\right), \end{aligned} \quad (20)$$

where the respective mean values of effective PSV and RI increment are given in Eq. 16 and the SDs $\Delta\theta^0_{\mathrm{eff}}$, $\Delta\alpha^0_{\mathrm{eff}}$ follow the SD of a mixture distribution (see Eq. S14).

With this, we further examine the implications of deviations of the relative lipid volume fraction Δx_{lip} from voxel to voxel. Such deviations may be interpreted as a form of inhomogeneity of the system, which have been experimentally quantified in cells and tissues by stimulated Raman spectroscopy (SRS) measurements (22). Employing Gaussian propagation of uncertainty, we find the following analytical expression of the deviation of the mean effective PSV and RI increment with respect to Δx_{lip} as

$$\begin{aligned} \Delta\theta_{\mathrm{eff}} &= \left[\left(\frac{\Delta\theta^0_{\mathrm{eff}}}{\sqrt{N_{\mathrm{s}}}}\right)^2 + \left(\frac{\partial\overline{\theta}_{\mathrm{eff}}}{\partial\overline{x}_{\mathrm{lip}}}\Delta x_{\mathrm{lip}}\right)^2\right]^{1/2}, \\ \Delta\alpha_{\mathrm{eff}} &= \left[\left(\frac{\Delta\alpha^0_{\mathrm{eff}}}{\sqrt{N_{\mathrm{s}}}}\right)^2 + \left(\frac{\partial\overline{\alpha}_{\mathrm{eff}}}{\partial\overline{x}_{\mathrm{lip}}}\Delta x_{\mathrm{lip}}\right)^2\right]^{1/2}. \end{aligned} \quad (21)$$

The partial derivatives in Eq. 21 are given in the Supporting Material (Eqs. S19 and S20). A visual depiction of Eq. 21 in dependence of the number of voxelinos per voxel $N_0 = N_{\mathrm{s}}/(1 - \varphi_1)$ and the corresponding results of MC simulations for certain parameter configurations are shown in Fig. 3 *A*.

As becomes apparent, the analytical solution employing Gaussian propagation of uncertainty is in concordance with the simulated values. However, we note that this is due to the assumption of normal distributions for the individual effective RI increments and PSVs. For nonnormal distributions, Gaussian propagation of uncertainty might not be applicable.

By assuming the mixture distribution given by Eq. 19, consequently, the deviations $\Delta\alpha_{\mathrm{eff}}$ and $\Delta\theta_{\mathrm{eff}}$ are maximized for $\overline{x}_{\mathrm{lip}} = 0.5$. Secondly, although for nonfluctuating x_{lip} (i.e., $\Delta x_{\mathrm{lip}} = 0$) the respective deviations of the effective RI increment and the PSV exhibit the $1/\sqrt{N_0}$ proportionality, for $\Delta x_{\mathrm{lip}} > 0$ we obtain nonvanishing deviations for a large number of voxelinos per voxel N_0.

Throughout this study we assume that all volume fractions, in particular x_{lip}, follow a normal distribution, truncated in the domain $[0,1]$, since values outside of this interval are nonphysical under the assumption of volume additivity. For a normal distribution with mean μ and SD σ, the corresponding truncated distribution is denoted by $\mathcal{T}(\mu,\sigma)$ (see Supporting Material). We note that another choice of distribution could be given by the beta distribution $\mathcal{B}(a,b)$ with shape parameters a and b, which is inherently bounded between 0 and 1. However, the interpretation of the shape parameters in this context is not as straightforward as the truncated normal distribution. Alternatively, a uniform distribution with a certain domain $[x^{\mathrm{min}}_{\mathrm{lip}}, x^{\mathrm{max}}_{\mathrm{lip}}]$ could be employed. For more than two types of macro molecules, the Dirichlet distribution could be of use. However, we expect a similar qualitative behavior for all mentioned distributions.

Solvent volume fraction

Next, we study the uncertainty associated to the solvent volume fraction φ_1, defined in Eq. 13, in dependence of the number of solute voxelinos N_{s} per voxel. By employing Gaussian propagation of uncertainty we find

$$\Delta\varphi_1 = (1 - \overline{\varphi}_1)\frac{\Delta N_{\mathrm{s}}}{\overline{N}_{\mathrm{s}}}, \quad (22)$$

where we used the presumption that the number of voxelinos per voxel N_0 is constant. From Eq. 22, we obtain that a change in the solvent content from voxel to voxel is directly proportional to a relative change in the number of solute voxelinos per voxel $\Delta N_{\mathrm{s}}/\overline{N}_{\mathrm{s}}$. However, it is not clear whether N_{s} follows a statistical distribution and, if so, how this distribution would be governed by active/passive processes in biological matter. An ingenuous guess is given by the equilibrium assumption that the number of solute voxelinos per voxel N_{s} is binomially distributed as

$$N_{\mathrm{s}} \sim \mathrm{Bin}(N_0, (1 - \overline{\varphi}_1)), \quad (23)$$

which may be interpreted as finding N_{s} out of N_0 voxelinos in a voxel with a probability of $1 - \overline{\varphi}_1$.

Besides a statistical argument, we may also compute a change in the number of solute voxelinos from voxel to voxel by considering $N_{\mathrm{s}} = (m_{\mathrm{s}}\theta_{\mathrm{eff}})/v_0$. Herewith, Eq. 22 may be written as

$$\Delta\varphi_1 = (1 - \overline{\varphi}_1)\sqrt{(\Delta\varphi^0_1)^2 + (\Delta\varphi^\infty_1)^2}, \quad (24)$$

with

$$\Delta\varphi_1^0 = \left\{ \frac{1}{N_0(1-\overline{\varphi}_1)} \left[\overline{\varphi}_1 + \left(\frac{\Delta\theta_{\text{eff}}^0}{\overline{\theta}_{\text{eff}}} \right)^2 \right] \right\}^{1/2},$$

$$\Delta\varphi_1^\infty = \left[\left(\frac{\Delta m_s}{\overline{m}_s} \right)^2 + \left(\frac{\partial\overline{\theta}_{\text{eff}}}{\partial\overline{x}_{\text{lip}}} \frac{\Delta x_{\text{lip}}}{\overline{\theta}_{\text{eff}}} \right)^2 \right]^{1/2}, \tag{25}$$

using the presumption of a constant voxelino volume $\Delta v_0 = 0$. This indicates that, similar to the effective RI increment and PSV, given by Eq. 18, the deviation of the solvent volume fraction $\Delta\varphi_1$ has two components; for once $\Delta\varphi_1^0$, which shows the $1/\sqrt{N_0}$ dependence, following the central limit theorem. Second, $\Delta\varphi_1^\infty$, which connects fluctuations in the solvent volume fraction to fluctuations in the solute mass and/or fluctuations in the solute composition. Hence, $\Delta\varphi_1^\infty$ may be interpreted as quantification of the degree of inhomogeneity of the sample connected to the amount of solute and its composition. Consequently, for a large number of voxelinos per voxel N_0, these inhomogeneities become the dominant contribution to the deviation of the solvent volume fraction.

We note that the presumption of a constant voxelino volume $\Delta v_0 = 0$ and a constant number of voxelinos per voxel $\Delta N_0 = 0$ from voxel to voxel is necessitated by the experimental boundary condition that all voxels have the same volume, i.e., $\Delta v_v = 0$.

RI

Considering the previous derivations of the uncertainties of the effective PSV and RI increment and the water volume fraction, we now examine the refractive index of the solution. Based on Eq. 7, we readily obtain an estimate of the uncertainty of n by employing Gaussian propagation of uncertainty (neglecting potential correlations) as

$$\Delta n = \left[\sum_i \left(\frac{\partial n}{\partial \beta_i} \Delta\beta_i \right)^2 \right]^{1/2}, \tag{26}$$

where the sum is taken over all $\beta = \{\alpha_{\text{eff}}, \theta_{\text{eff}}, \varphi_1\}$ and the respective deviations $\Delta\beta_i$ are given in Eqs. 21 and 24. The graphical representation of Eq. 26 in dependence of N_0 is given in Fig. 3 *A*.

As a consequence, for a vanishing relative deviation of the solute $\Delta m_s/\overline{m}_s$, the $1/\sqrt{N_0}$ scaling behavior of the effective RI increment and PSV determines the scaling behavior of the deviations in the RI Δn. However, for $\Delta m_s/\overline{m}_s > 0$, we obtain a constant deviation of the RI for large N_0, consistent with the broad RI distributions obtained by ODT measurements of different cells and tissues (12,16,21,51) and fluctuations in the water volume fraction measured by SRS (22). Furthermore, although the dependence of Δn on the mean relative lipid volume fraction $\overline{x}_{\text{lip}}$ is determined by the deviations of the effective RI increment and PSV, we observe a vanishing impact of the deviation of the relative lipid volume fraction Δx_{lip}. This fact is due to the numerically small differences of the mean refractions per gram of the particular choice of proteins and the lipid $|\overline{R}_{\text{lip}} - \overline{R}_{\text{p}}| \approx 0.05$ in combination with larger differences of the mean PSVs $|\overline{\theta}_{\text{lip}} - \overline{\theta}_{\text{p}}| \approx 0.35$ (see Fig. S1).

MD

Finally, we investigate the uncertainty associated with the MD. From Eq. 15 we obtain the corresponding deviation, employing Gaussian propagation of uncertainty, as

$$\Delta\rho = \left[\left(\frac{\overline{\theta}_{\text{eff}}\rho_1 - 1}{\overline{\theta}_{\text{eff}}} \Delta\varphi_1 \right)^2 + \left(\frac{\overline{\varphi}_1 - 1}{\overline{\theta}_{\text{eff}}^2} \Delta\theta_{\text{eff}} \right)^2 \right]^{1/2}, \tag{27}$$

displayed in Fig. 3 *A*. As discussed for Δn, the magnitude of $\Delta\rho$ is affected by the mean relative lipid fraction $\overline{x}_{\text{lip}}$ as a consequence of the mixture distribution, given in Eq. 14. Furthermore, we have a nonvanishing deviation of the MD for $\Delta m_s/\overline{m}_s > 0$ and large N_0. To no surprise, we observe a remarkable impact of deviation of the relative lipid fraction Δx_{lip} on $\Delta\rho$ due to the strong scaling with the deviation of the effective PSV $\Delta\theta_{\text{eff}}$.

Correlation of RI and MD

Having studied the deviations associated with RI and MD, we next sought to illuminate the correlation between the distributions of the RI contrast δn, and the MD ρ, denoted by $\mathcal{P}(\rho, \delta n)$, in dependence of the model parameters introduced earlier. To that aim, we performed MC simulations of Eq. 15 for the case of human proteins and triolein in water for a range of different mean relative lipid and water volume fractions, as shown in Fig. 3 *B*.

Besides the intuitive behavior of $\rho(\delta n)$ for the cases of $\overline{x}_{\text{lip}} = 0$ (MD increases with increasing RI) and $\overline{x}_{\text{lip}} = 1$ (MD decreases with increasing RI), for $\overline{x}_{\text{lip}} = 0.8$ the MD is roughly constant for all RI values. This feature is quite remarkable since it demonstrates that, for certain solute compositions, the MD is decoupled from the RI for all possible water volume fractions, i.e., $\partial\rho/\partial n = 0 \Rightarrow \theta_{\text{eff}}(\overline{x}_{\text{lip}}) = 1/\rho_1$. Furthermore, as motivated earlier, and shown in Fig. 3 *C*, measuring a RI distribution (e.g., via ODT) may correspond to a range of different water and relative lipid volume fractions, resulting in drastically different estimates on the distribution of the MD from case to case. This in turn

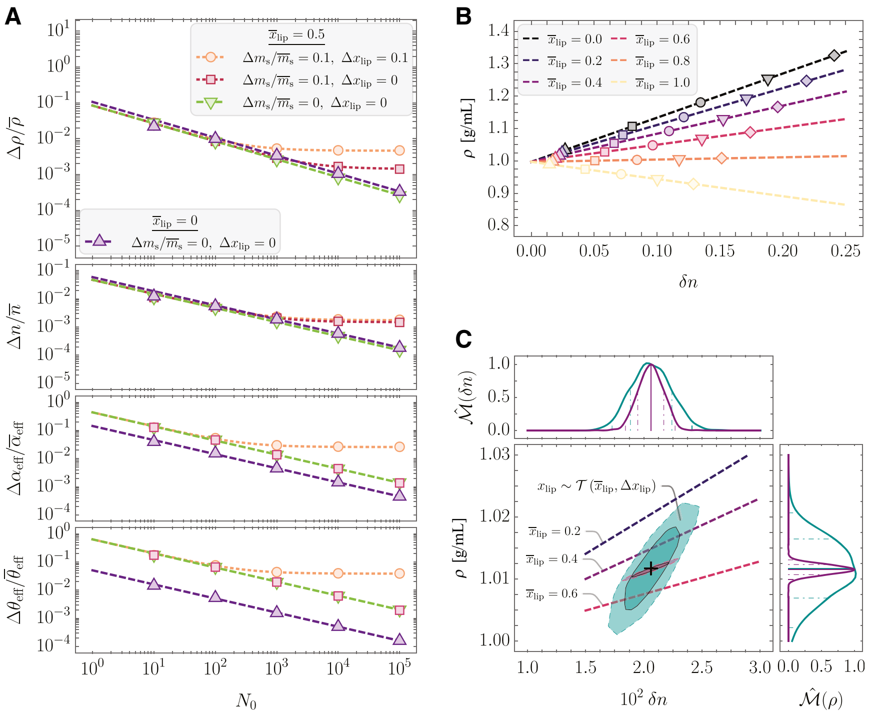

FIGURE 3 Results of MC simulations and according analytical solutions for the mixture of human proteins and the neutral lipid triolein in water. (*A*) Relative deviations of the MD ρ, the RI n, the effective RI increment $\alpha_{\rm eff}$, and PSV $\theta_{\rm eff}$ in dependence of the number of voxelinos per voxel N_0 obtained from MC simulations (*symbols*) and analytical solutions (*dashed lines*) for different mean relative lipid volume fractions $\overline{x}_{\rm lip}$, associated deviations $\Delta x_{\rm lip}$, and relative deviations of the number of the solute mass $\Delta m_{\rm s}/\overline{m}_{\rm s}$. The MC simulations were performed for a mean water volume fraction of $\overline{\varphi}_1 = 0.9$ and $N_{\rm v} = 10^3$. (*B*) MD ρ in dependence of the RI contrast δn for different mean relative lipid volume fractions $\overline{x}_{\rm lip}$ and mean water volume fractions (◇ $\overline{\varphi}_1 = 0.1$, ▽ $\overline{\varphi}_1 = 0.3$, ◯ $\overline{\varphi}_1 = 0.5$, □ $\overline{\varphi}_1 = 0.7$, △ $\overline{\varphi}_1 = 0.9$) for $\Delta x_{\rm lip} = 0$, $N_0 = 10^3$ and $N_{\rm v} = 10^2$. The dashed lines indicate the analytical solutions of Eq. 15. (*C*) Correlative distribution of the MD ρ and the RI contrast δn (the solid and dashed lines indicate the 68% and 95% confidence contours) with the corresponding normalized marginal probability density distributions $\widehat{\mathcal{M}}(\rho)$ and $\widehat{\mathcal{M}}(\delta n)$, respectively (the solid line represents the median, the dash-dotted and dashed lines indicate the 68% and 95% CIs), for the exact relative lipid volume fraction $\overline{x}_{\rm lip} = 0.5$ (*purple*) and the relative lipid volume fraction following a truncated normal distribution $x_{\rm lip} \sim \mathcal{T}(\overline{x}_{\rm lip} = 0.5, \Delta x_{\rm lip} = 0.1)$ (*cyan*; see main text). The MC simulations were performed for a water volume fraction following a truncated normal distribution $\varphi_1 \sim \mathcal{T}(\overline{\varphi}_1 = 0.9, \Delta\varphi_1 = 0.1)$, $N_0 = 10^5$ and $N_{\rm v} = 10^3$.

strongly motivates the necessity for detailed knowledge about not only the solute composition but also the solvent content of the sample.

In light of the theoretical implications delineated above, in the next step we want to examine the predictive capabilities of the model for a set of physiological complex mixtures that are well characterized in terms of their solute composition.

Experimental validation and application

In the following, we scrutinize the applicability of previous findings to a set of well-characterized physiological substances: bovine SM (Millipore 70166) and 20% IL (Sigma-Aldrich I141), commonly used as compounds in tissue-mimicking samples because of their optical properties (52–57).

To that aim, we measured the solute-concentration-dependent RI and MD with an Abbe refractometer and a pycnometer, respectively. Both samples are particularly intriguing since they should exhibit different $\rho(\delta n)$ dependencies; SM mainly consists of lactose and milk proteins, whereas IL is a stabilized emulsion of soybean oil (see Fig. 3 *B* for a reference).

According to the chemical certificate of analysis provided by the manufacturers, the SM exhibits $y_1^{\rm SM} = 4\%$ and the IL exhibits $y_1^{\rm IL} = 76\%$ of water. Hence, we computed the respective solute concentrations as $c_{\rm s}^{\rm k} = y_{\rm s}^{\rm k}\rho_{\rm k}$, where $\rho_{\rm k}$ is the measured MD of the sample (SM or IL) at a given dilution, $y_{\rm s}^{\rm k} = m_{\rm k}(1 - y_1^{\rm k})/(m_{\rm k} + m_{\rm w}^{\rm k})$ is the solute mass fraction, $m_{\rm k}$ denotes the mass of the sample, and $m_{\rm w}^{\rm k}$ is the mass of water added to the sample. The results of the measurements are shown in Fig. 4 *A* and *B*, where each point represents $N = 5$ technical repetitions.

Using Eqs. 6 and 8, we fitted the data to obtain experimental values of $\theta_{\rm eff}$ and $\alpha_{\rm eff}$ with according uncertainties, respectively, for both SM and IL. Examining the fitting residuals, the RI contrast and MD scale linearly with the solute concentration, justifying our model assumptions of the Biot Eq. 7 rule and volume additivity.

We then employed the information about the chemical composition of the respective substances, as provided by the manufacturers (see Table S2), to compute the correlative RI contrast and MD according to Eq. 15, employing MC sampling. A graphical representation of the respective RI increments and PSVs of all substances considered, as well as the experimentally determined and predicted values of SM and IL, is given in Fig. 4 *C*.

As becomes apparent, the measured and predicted values of the SM and IL are in good agreement, whereas potential uncertainties regarding the exact chemical composition might be underappreciated here, as we have no means of estimating them. To

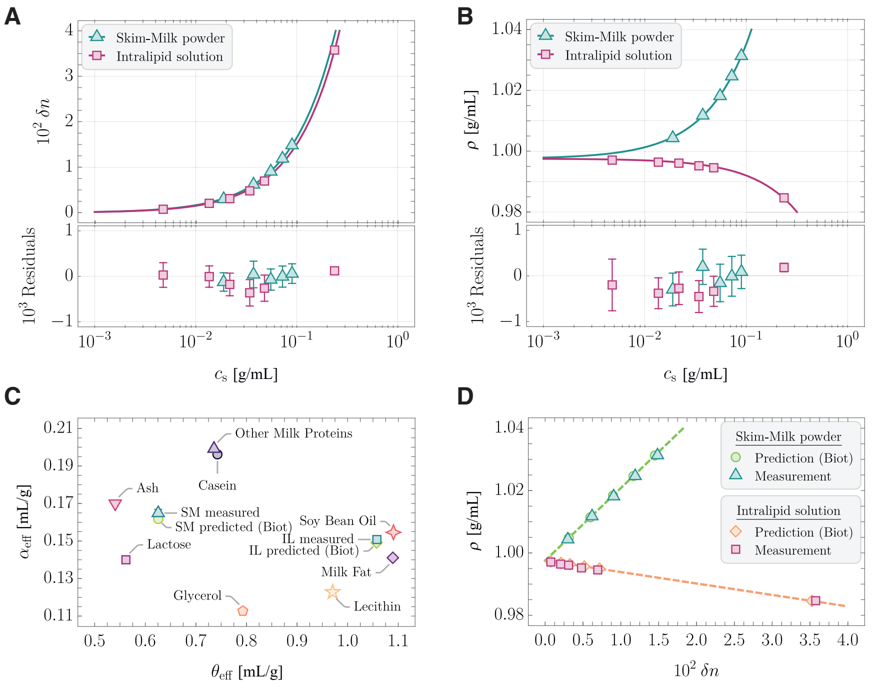

FIGURE 4 Results of the concentration-dependent measurements of the MD and RI of bovine SM and 20% IL in water as well as the theoretical predictions based on the chemical composition using Eq. 15. (*A*) RI contrast δn in dependence of the solute concentration c_s of SM ($\triangle$, $N = 5$ technical repetitions, mean $\pm$ SD) and IL ($\square$, $N = 5$ technical repetitions, mean $\pm$ SD) with the respective fits of Eq. 8 (*solid lines*) and fit residuals. (*B*) MD ρ in dependence of the solute concentration c_s of SM ($\triangle$, $N = 5$ technical repetitions, mean $\pm$ SD) and IL ($\square$, $N = 5$ technical repetitions, mean $\pm$ SD) with the respective fits of Eq. 6 (*solid lines*) and fit residuals. (*C*) Effective RI increment α_{eff} and PSV θ_{eff} of various substances that compose SM and IL, as well as the measured and predicted values for SM and IL. (*D*) MD ρ in dependence of the RI contrast δn for different concentrations of SM in water and IL. The symbols represent measured values ($N = 5$ technical repetitions, mean $\pm$ SD) and the predicted values using the Biot mixing rule (Eq. 7) for $N_0 = 10^4$ and $N_v = 10^3$. The dashed lines indicate Eq. 15 for the predicted values of the effective RI increment and PSV.

further examine the goodness of our model, we compared the solute RIs obtained from fitting the data with various RI mixing rules and the according prediction, based on the chemical composition of the samples, as described in the Supporting Material (Table S3). By this analysis, we found the Biot Eq. 7 to yield the best agreement among a selection of RI mixture rules.

Furthermore, matching the experimental water volume fractions $\varphi_1 = (\rho_k - c_s^k)/\rho_1$, we obtained a prediction of the MD in dependence of the RI, which was found to coincide well with the measurements for both SM and IL, as shown in Fig. 4 *D*.

Larval zebrafish trunk tissue

Having delineated the validity of Eq. 9 in the context of complex mixtures, we examined the capability of the proposed model in an in vivo scenario. To that aim, we chose the larval zebrafish model system at 96 h post fertilization (hpf), for which MS and RI data (employing ODT) of the trunk tissue were recently obtained (21). The RI data are given in Table S4. Because the MS data provide the protein content, we are able to estimate the RI increment and PSV distribution of the proteins present in the tissue, as demonstrated earlier, following Zhao et al. (25) (see Table 1).

In the following, we assume that the larval zebrafish trunk tissue primarily consists of mentioned proteins, lipids, and water, based on the findings of (58,59).

Long et al. (58) determined the wet mass m_{wet} and dry mass m_{dry} of larval zebrafish at 96 hpf by weighting (dried) pooled larvae (see their Table 1). Furthermore, they estimated the protein mass m_p and lipid mass m_{lip} of larvae by measuring the optical absorbance using bovine serum albumin and corn oil as calibration materials, respectively. Notably, the measurements of Long et al. (58) were performed on whole animals, including the yolk sac, which is rich in lipids. Hence, the assumed relative volume fraction of the lipids and the water volume fraction are likely to be different from the trunk tissue.

The lipid composition of zebrafish larvae was determined by Hachicho et al. (59), from which we adopted the four phospholipid fatty acids (PLFAs) with the highest abundance. The respective relative volume fractions of the PLFAs for zebrafish larvae at 96 hpf were roughly digitally obtained from Fig. 3 of Hachicho et al. (59) and are provided in Table S2. These four PLFAs make up about 72% of the total PLFA amount in the zebrafish larva. Additionally, based on (59), we assumed that the overall lipid composition of the tissue is only given by triolein and mentioned PLFAs.

Using these estimations of the biochemical composition of the larvae and the corresponding material properties given in Table S2, we inferred the distributions of the relative lipid volume fraction $x_{lip} = 0.220 \pm 0.022$ and the water volume fraction $\varphi_1 = 0.860 \pm 0.006$ (Eqs. S23 and S24)). Employing Eq. 15, we obtained the correlative RI and MD distribution $\mathcal{P}(\delta n, \rho)$ by MC sampling shown in Fig. 5. We then computed the marginalized distributions $\widehat{\mathcal{M}}(\delta n)$ and $\widehat{\mathcal{M}}(\rho)$ from which we obtained the following median values and 68%

confidence intervals (CIs) $\rho = (1.0341 \pm 0.0024)$ g/mL and $n_B = 1.3675 \pm 0.0017$. Comparing n_B with the RI measurements $n_{meas} = 1.3655^{+0.0028}_{-0.0032}$, evidently, although both values coincide within the 68% CIs, our prediction yields a narrower distribution compared to the measurement, which may be attributed to the narrow assumed distribution of φ_1. Using the Lorentz-Lorenz mixing rule of RIs (Eq. S8), we obtained $n_{LL} = 1.3650 \pm 0.0014$, which coincides better with the measured value. However, as pointed out earlier, the Lorentz-Lorenz mixing rule did not yield results that coincided well with the measurements of the validation samples shown above.

In essence, at present, it is not clear which RI mixing rule should be employed in this in vivo scenario without a more comprehensive and quantitative understanding of the biochemical composition of the sample. However, once this insight becomes available (e.g., by measuring (S)RS), different mixing rules could be compared to each other, as presented earlier. This in turn would also allow us to study whether the best-fitting RI mixing rule is conserved across different specimen.

Finally, we want to point out that, if we use the customary simplifying assumption that the solute composition of the trunk tissue is only given by proteins, employing Eq. 9, the RI increment and PSV given in Table 1 and the measured RI data (Table S4), we obtain $\rho_p = (1.040 \pm 0.004)$ g/mL, using Gaussian propagation of uncertainty. Apparently, this value does not coincide well with the value determined above. In fact, as outlined earlier, not taking lipids into account results in a systematic overestimation of the MD of biological matter.

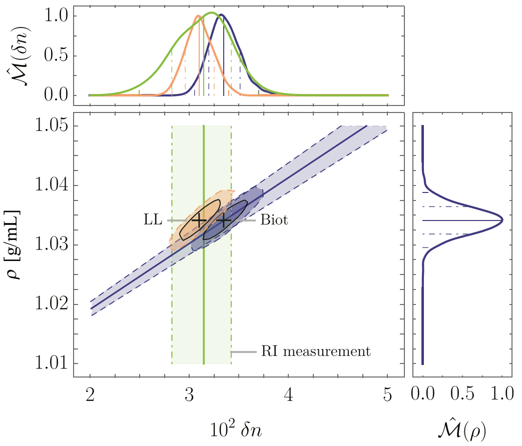

FIGURE 5 Results of MC simulations and according analytical solutions for the trunk tissue of larval zebrafish. Predicted correlative distributions of the MD ρ and the RI contrast δn of larval zebrafish trunk tissue with the corresponding normalized marginal probability density distributions $\widehat{\mathcal{M}}(\rho)$ and $\widehat{\mathcal{M}}(\delta n)$, respectively (the solid line represents the median, the dash-dotted and dashed lines indicate the 68% and 95% CIs). The ellipsoids indicate the predictions using Eq. 9 (blue) and the Lorentz-Lorenz mixing rule of RIs Eq. S8 (*orange*). The green band indicates the RI measurement of (21). The MC simulations were performed for $N_0 = 10^5$ and $N_v = 10^3$.

DISCUSSION

Considering the complexity of the chemical composition of biological matter, to infer the optical MD from an RI measurement is not well established in contemporary literature. Here, we present a theoretical macroscopic model that is capable of describing the problem, employing a minimal set of assumptions, namely the Biot mixing rule of RIs and the assumption of volume additivity.

We evaluated the possible sources of uncertainties associated with the model and showed that, based on the chemical composition of the sample and the associated degree of inhomogeneity, the resulting RI and MD distributions might drastically differ from the customary assumption of biological matter consisting of proteins and water only. For that purpose, we provided analytical solutions and consistent simulation results for the case of a binary solute composed of proteins and lipids.

Although it is shown that, for singular proteins in solution, the assumption of volume additivity might not be justified (60) (see Supporting Material), we provided experimental evidence that, for the set of validation samples under investigation (bovine SM and 20% IL), it holds well within the measurement uncertainties.

Further, we provided evidence that the predictions of the correlative MD and RI based on our model agree with the experimentally obtained values of the validation samples, thus establishing confidence in the application of the theoretical considerations presented here to estimate MD in dependence of the RI, given the chemical composition of the sample under study.

When applying the model to an in vivo specimen (i.e., the trunk tissue of the larval zebrafish), we observed that the mean value of RI measurements of (21) coincides within the 68% CIs of our predictions, which were based on the estimations of the biochemical composition of the tissue, employing the measurements of Long et al. (58), where the authors determined the masses of water, proteins, and lipids of whole animals. The lipid composition of the tissue was estimated based on the measurements of Hachicho et al. (59). Although our initial predictions are remarkably close to the measurements, given the multitude of assumptions and simplifications about the biochemical composition of the tissue employed, we cannot completely exclude crude uncertainties.

Going forward, the estimation of the MD of biological matter from RI measurements, as outlined in this study, will have interesting implications for inferring the mechanical properties from opto-acoustical

measurements (e.g., via Brillouin microscopy). The problem of a varying solute composition within a sample is well appreciated (see, e.g., (12)) but is not resolved in a cohesive manner. Evidently, combining Brillouin microscopy not only with ODT but also incorporating the local biochemical composition (e.g., via (S)RS measurements) allows for a better estimation of the longitudinal (elastic) modulus. We note that the aforementioned problem could also be resolved by performing stimulated Brillouin microscopy in combination with ODT. Here, the MD can be obtained directly from measurements of the Brillouin resonance gain factor, which in turn is connected to the Brillouin gain and the pump laser power, as well as the RI, Brillouin frequency shift, and Brillouin line width (61). Accurately determining the local (in vivo) MD will conceivably enable a more profound interpretation of functional mechanisms at play in biological matter.

To make the application of the findings of this study more accessible, we delineate strategies on how to estimate MD, given certain experimental paradigms, in the Supporting Material (see Fig. S2).

DATA AND CODE AVAILABILITY

The data and code that support the findings of this study are available upon reasonable request from the authors.

SUPPORTING MATERIAL

Supporting material can be found online at https://doi.org/10.1016/j.bpr.2024.100156.

AUTHOR CONTRIBUTIONS

J.G., C.M., and T.B. conceptualized the project. C.M. developed the methodology and conducted the experiments. C.M., J.K., and D.W. performed formal analysis of the data. All authors interpreted the experimental results. C.M. conducted the theoretical considerations. C.M., T.B., S.K., S.A., and V.Z. interpreted the theoretical results. C.M. wrote the original draft of this manuscript. All authors edited and improved the manuscript. J.G., D.W., and V.Z. supervised the project.

ACKNOWLEDGMENTS

We thank Abin Biswas, Giulia Zanini, and Giuliano Scarcelli for the helpful and illuminating discussions on the topic and Thomas Fleming for enhancing the language and clarity of this manuscript. The authors gratefully acknowledge the funding from the Max Planck Society to J.G. and the Deutsche Forschungsgemeinschaft (project number 460333672 – CRC1540 Exploring Brain Mechanics (subproject B05 to D.W.)).

DECLARATION OF INTERESTS

The authors declare no competing interests.

SUPPORTING CITATIONS

The following references appear in the Supporting Material (62–68).

REFERENCES

1. Guck, J., S. Schinkinger, C. Bilby…, 2005. Optical Deformability as an Inherent Cell Marker for Testing Malignant Transformation and Metastatic. *CompetenceBiophys. J.* 88:3689.
2. Engler, A. J., S. Sen, D. E. Discher…, 2006. Matrix Elasticity Directs Stem Cell Lineage Specification. *Cell.* 126:677.
3. Egan, P., R. Sinko, …, S. Keten. 2015. The role of mechanics in biological and bio-inspired systems. *Nat. Commun.* 6:7418.
4. Biswas, A., O. Munoz, S. Reber…, 2023. Conserved nucleocytoplasmic density homeostasis drives cellular organization across eukaryotes. Preprint at bioRxiv. https://doi.org/10.1101/2023.09.05.556409.
5. Iida, S., S. Ide, …, K. Maeshima. 2024. Orientation-Independent-DIC imaging reveals that a transient rise in depletion force contributes to mitotic chromosome condensation. Preprint at bioRxiv. https://doi.org/10.1101/2023.11.11.566679.
6. Watson, J. L., E. Seinkmane, …, E. Derivery. 2023. Macromolecular condensation buffers intracellular water potential. *Nature.* 623:842–852.
7. Schürmann, M., J. Scholze, …, C. J. Chan. 2016. Cell nuclei have lower refractive index and mass density than cytoplasm. *J. Biophot.* 9:1068–1076.
8. Biswas, A., K. Kim, …, S. Reber. 2021. The Xenopus spindle is as dense as the surrounding cytoplasm. *Dev. Cell.* 56:967–975.e5.
9. Antonacci, G., and S. Braakman. 2016. Biomechanics of subcellular structures by non-invasive Brillouin microscopy. *Sci. Rep.* 6:1.
10. Yan, G., S. Monnier, T. Dehoux…, 2022. Probing molecular crowding in compressed tissues with Brillouin light scattering. *Proc. Natl. Acad. Sci. USA.* 119, e2113614119.
11. Scarcelli, G., W. J. Polacheck, …, S. H. Yun. 2015. Noncontact three-dimensional mapping of intracellular hydromechanical properties by Brillouin microscopy. *Nat. Methods.* 12:1132–1134.
12. Schlüßler, R., K. Kim, …, J. Guck. 2022. Correlative all-optical quantification of mass density and mechanics of subcellular compartments with fluorescence specificity. *Elife.* 11, e68490.
13. Kennedy, B. F., and J. C. Bamber. 2021. *In* "emph "bibinfo booktitle Optical Coherence Elastography: Imaging Tissue Mechanics on the Micro-Scale AIP Publishing LLC.
14. Larin, K. V., and D. D. Sampson. 2017. Optical coherence elastography – OCT at work in tissue biomechanics [Invited]. *Biomed. Opt Express.* 8:1172.
15. Park, Y., C. Depeursinge, and G. Popescu. 2018. Quantitative phase imaging in biomedicine. *Nat. Photonics.* 12:578–589.
16. Kim, K., and J. Guck. 2020. The Relative Densities of Cytoplasm and Nuclear Compartments Are Robust against Strong Perturbation. *Biophys. J.* 119:1946–1957.
17. BARER, R. 1952. Interference Microscopy and Mass Determination. *Nature.* 169:366–367.
18. Bhaduri, B., H. Pham, …, G. Popescu. 2012. Diffraction phase microscopy with white light. *Opt. Lett.* 37:1094.
19. Schlüßler, R., S. Möllmert, …, J. Guck. 2018. Mechanical Mapping of Spinal Cord Growth and Repair in Living Zebrafish Larvae by Brillouin Imaging. *Biophys. J.* 115:911–923.
20. Rollin, R., J.-F. Joanny, and P. Sens. 2023. Physical basis of the cell size scaling laws. *Elife.* 12, e82490.
21. Kolb, J., V. Tsata, …, D. Wehner. 2023. Small leucine-rich proteoglycans inhibit CNS regeneration by modifying the structural

and mechanical properties of the lesion environment. *Nat. Commun.* 14:6814.

22. Oh, S., C. Lee, …, M. W. Kirschner. 2022. Protein and lipid mass concentration measurement in tissues by stimulated Raman scattering microscopy. *Proc Natl Acad Sci USA.* 119. e2117938119. https://doi.org/10.1073/pnas.2117938119.

23. Alunni Cardinali, M., A. Di Michele, …, D. Fioretto. 2022. Brillouin–Raman microspectroscopy for the morpho-mechanical imaging of human lamellar bone. *J. R. Soc. Interface.* 19, 20210642.

24. Abuhattum, S., P. Kotzbeck, …, A. V. Taubenberger. 2022. Adipose cells and tissues soften with lipid accumulation while in diabetes adipose tissue stiffens. *Sci. Rep.* 12, 10325.

25. Zhao, H., P. H. Brown, and P. Schuck. 2011. On the distribution of protein refractive index increments. *Biophys. J.* 100:2309–2317.

26. W. R. Inc.. 2020. Mathematica, Version 12.2.

27. Durchschlag, H. 1986. Specific volumes of biological macromolecules and some other molecules of biological interest. Springer Berlin Heidelberg, Berlin, Heidelberg, pp. 45–128.

28. Heller, W.; The Journal of Physical Chemistry. 1965. Remarks on Refractive Index Mixture Rules. *J. Phys. Chem.* 69:1123–1129.

29. Hanley, B. F. 2020. A practical method for estimating specific refractive index increments for flexible non-electrolyte polymers and copolymers in pure and mixed solvents using the Gladstone-Dale and Lorentz-Lorenz equations in conjunction with molar refraction structural constants, and solvent physical property databases. *Mater. Today Commun.* 25, 101644.

30. PERKINS, S. J. 1986. Protein volumes and hydration effects. The calculations of partial specific volumes, neutron scattering matchpoints and 280-nm absorption coefficients for proteins and glycoproteins from amino acid sequences. *Eur. J. Biochem.* 157:169–180.

31. Aknoun, S., M. Yonnet, …, P. Pognonec. 2021. Quantitative phase microscopy for non-invasive live cell population monitoring. *Sci. Rep.* 11:4409.

32. Rogers, J. D., A. J. Radosevich, …, V. Backman. 2014. Modeling Light Scattering in Tissue as Continuous Random Media Using a Versatile Refractive Index Correlation Function. *IEEE J. Sel. Top. Quant. Electron.* 20:173–186.

33. Mir, M., Z. Wang, G. Popescu…, 2011. Optical measurement of cycle-dependent cell growth. *Proc. Natl. Acad. Sci. USA.* 108:13124.

34. Popescu, G., Y. Park, …, K. Badizadegan. 2008. Optical imaging of cell mass and growth dynamics. *Am. J. Physiol. Cell Physiol.* 295:C538–C544.

35. Cooper, K. L., S. Oh, …, C. J. Tabin. 2013. Multiple phases of chondrocyte enlargement underlie differences in skeletal proportions. *Nature.* 495:375–378.

36. Barr, E. S. 1955. Concerning Index of Refraction and Density. *Am. J. Phys.* 23:623–624.

37. Consortium, T. U. 2022. *Nucleic Acids Res. UniProt: the Universal Protein Knowledgebase in 2023.* 51:D523.

38. Mayerhöfer, T. G., and J. Popp. 2020. Beyond Beer's Law: Revisiting the Lorentz-Lorenz Equation. *ChemPhysChem.* 21:1218–1223.

39. C. E. J.. 1943. Proteins, Amino Acids and Peptides as Ions and Dipolar Ionsnewspace 370.

40. Pretorius, F., W. W. Focke, …, E. du Toit. 2021. Estimating binary liquid composition from density and refractive index measurements: A comprehensive review of mixing rules. *J. Mol. Liq.* 332, 115893.

41. Reis, J. C. R., I. M. S. Lampreia, …, G. Douhéret. 2010. Refractive Index of Liquid Mixtures: Theory and Experiment. *ChemPhysChem.* 11:3722–3733.

42. Brocos, P., Á. Piñeiro, …, A. Amigo. 2003. Refractive indices, molar volumes and molar refractions of binary liquid mixtures: concepts and correlations. *Phys. Chem. Chem. Phys.* 5:550–557.

43. Sihvola, A. 2000. Subsurface Sensing Technologies and Applications, 1, p. 393.

44. Acevedo-Barrera, A., and A. Garcia-Valenzuela. 2019. Analytical approximation to the complex refractive index of nanofluids with extended applicability. *Opt Express.* 27:28048–28061.

45. Meiers, D. T., and G. von Freymann. 2023. Mixing rule for calculating the effective refractive index beyond the limit of small particles. *Opt Express.* 31, 32067.

46. Garahan, A., L. Pilon, …, I. Saxena. 2007. Effective optical properties of absorbing nanoporous and nanocomposite thin films. *J. Appl. Phys.* 101, 014320.

47. Smith, D., J. S. Myers, …, C. Goodman-Strauss. 2023. An aperiodic monotile, arXiv:2303.10798 (math.CO).

48. Chorlay, A., and A. R. Thiam. 2020. Neutral lipids regulate amphipathic helix affinity for model lipid droplets. *J. Cell Biol.* 219, e201907099. https://doi.org/10.1083/jcb.201907099.

49. Onal, G., O. Kutlu, …, S. Dokmeci Emre. 2017. Lipid Droplets in Health and Disease. *Lipids Health Dis.* 16:128.

50. Thiam, A. R., and E. Ikonen. 2021. Lipid Droplet Nucleation. *Trends Cell Biol.* 31:108–118.

51. Kim, K., V. R. Gade, …, J. Guck. 2022. Quantitative imaging of Caenorhabditis elegans dauer larvae during cryptobiotic transition. *Biophys. J.* 121:1219–1229, "natexlaba).

52. Vidallon, M. L. P., E. Salimova, …, A. I. Bishop. 2022. Enhanced photoacoustic imaging in tissue-mimicking phantoms using polydopamine-shelled perfluorocarbon emulsion droplets. *Ultrason. Sonochem.* 86, 106041.

53. Lai, P., X. Xu, and L. V. Wang. 2014. Dependence of optical scattering from Intralipid in gelatin-gel based tissue-mimicking phantoms on mixing temperature and time. *J. Biomed. Opt.* 19, 035002.

54. Cook, J. R., R. R. Bouchard, and S. Y. Emelianov. 2011. Tissue-mimicking phantoms for photoacoustic and ultrasonic imaging. *Biomed. Opt Express.* 2:3193.

55. Bachir, W., and R. khir. 2022. Characterization of pasteurized milk in the near infrared range for construction of tissue-mimicking optical phantoms. *Opt. Mater. X.* 14, 100154.

56. Stiles, T. A., E. L. Madsen, …, J. A. Lucey. 2005. Tissue-Mimicking Liquid for Use in Exposimetry. *J. Ultrasound Med.* 24:501–516.

57. McGarry, C. K., L. J. Grattan, C. H. Clark…, 2020. Tissue mimicking materials for imaging and therapy phantoms: a review. *Phys. Med. Biol.* 65:23TR01.

58. Long, Y., L. Li, Z. Cui…, 2012. Transcriptomic Characterization of Temperature Stress Responses in Larval Zebrafish. *PLoS One.* 7:1.

59. Hachicho, N., S. Reithel, T. Luckenbach…, 2015. Body Mass Parameters, Lipid Profiles and Protein Contents of Zebrafish Embryos and Effects of 2,4-Dinitrophenol Exposure. *PLoS One.* 10:1.

60. Zamyatnin, A. 1972. Progress in Biophysics and Molecular Biology, 24, p. 107.

61. Boyd, R. W. 2008. *In* "emph "bibinfo booktitle Nonlinear Optics, Third Edition, R. W. Boyd, ed Academic Press, pp. 429–471.

62. Kim, S., J. Chen, …, E. E. Bolton. 2022. PubChem 2023 update. *Nucleic Acids Res.* 51:D1373–D1380.

63. 2022. Wolfram Research, Inc., Wolfram |Alpha Knowledgebase, Champaign, IL (2022).

64. McMeekin, T. L., M. Wilensky, and M. L. Groves. 1962. Refractive indices of proteins in relation to amino acid composition and specific volume. *Biochem. Biophys. Res. Commun.* 7:151–156.

65. Bansal, N., T. Truong, and B. Bhandari. 2017. Feasibility study of lecithin nanovesicles as spacers to improve the solubility of milk protein concentrate powder during storage. *Dairy Sci. Technol.* 96:861–872.

66. Oxford Lab Fine Chem LLP. F020Soybean lecithin. https://www.oxfordlabchem.com/msds/(S-08441)SOYA%20LECITHIN%2030%20Extra%20Pure.pdf.

67(a) ChemSpider. Triolein, CSID:4593733. http://www.chemspider.com/Chemical-Structure.4593733.html.

67(b) ChemSpider. Palmitic acid , CSID:960. http://www.chemspider.com/Chemical-Structure.960.html.

67(c) ChemSpider. Oleic acid, CSID:393217. http://www.chemspider.com/Chemical-Structure.393217.html.

67(d) ChemSpider. Docosahexaenoic acid, CSID:393183. http://www.chemspider.com/Chemical-Structure.393183.html.

67(e) ChemSpider. Stearic acid, CSID:5091. http://www.chemspider.com/Chemical-Structure.5091.html.

68(a) Merck. Soybean oil, 47122. https://www.sigmaaldrich.com/DE/de/product/supelco/47122,

68(b) Merck. Glycerol, 104057. https://www.merckmillipore.com/DE/de/product/Glycerol,MDA_CHEM-104057.

Biophysical Reports, Volume *4*

Supplemental information

Estimation of the mass density of biological matter from refractive index measurements

Conrad Möckel, Timon Beck, Sara Kaliman, Shada Abuhattum, Kyoohyun Kim, Julia Kolb, Daniel Wehner, Vasily Zaburdaev, and Jochen Guck

Supporting Material: Estimations of the mass density of biological matter from refractive index measurements

I. PARTIAL AND APPARENT SPECIFIC VOLUMES AND THE REFRACTIVE INDEX INCREMENT

Based on derivations in [1], for a binary solution consistent of N_1 moles of solvent with molar volume V_1 and N_2 moles of solute, the total molar volume is given by

$$\begin{aligned} V &= N_1 V_1 + N_2 \tilde{\Theta}, \text{ or} \\ \tilde{\Theta} &= \frac{V - N_1 V_1}{N_2}, \end{aligned} \tag{S1}$$

where $\tilde{\Theta}$ denotes the apparent molar volume of the solute. This parameter accounts for the potential volume-inadditivity; in other words, it is a macroscopic quantity that describes the volume of the solute in dilution. The partial molar volume is given by

$$\Theta = \left(\frac{\partial V}{\partial N_2}\right)_{T,p,N_1} = N_2 \left(\frac{\partial \tilde{\Theta}}{\partial N_2}\right)_{T,p,N_1} + \tilde{\Theta}, \tag{S2}$$

and the apparent and partial specific volume can be expressed with the molar mass of the solute M_2 as $\vartheta = \tilde{\Theta}/M_2$ and $\theta = \Theta/M_2$, respectively.

A. Volume additivity

If the volume additivity is given, then

$$\begin{aligned} \Theta = V_2 = \tilde{\Theta} &= \frac{M_2}{\rho_2}, \\ \theta = \vartheta = 1/\rho_2 &= \frac{V_2 N_\mathrm{A}}{M_2}. \end{aligned} \tag{S3}$$

B. Protein PSV and RI increment

As employed in [1–4], the PSV and refraction per gram of a protein may be interpreted the weight average of all amino acids composing this protein as

$$\begin{aligned} \theta_\mathrm{p} &= \frac{\sum_i V_{\mathrm{r},i} N_\mathrm{A}}{\sum_i M_{\mathrm{r},i} + M_\mathrm{w}}, \\ R_\mathrm{p} &= \frac{\sum_i R^*_{\mathrm{r},i}}{\sum_i M_{\mathrm{r},i} + M_\mathrm{w}}, \end{aligned} \tag{S4}$$

where $M_\mathrm{w} = 18.01$ g/mol is the molecular mass of water [5, 6] and N_A is the Avogadro constant. We note that, without loss of generality, setting $m_i = M_i/N_\mathrm{A}$, i.e. $n_i = 1/N_\mathrm{A}$, Eq. (S4) can be expressed as

$$\begin{aligned} \theta_\mathrm{p} &= \sum_i \frac{m_{\mathrm{r},i} \theta_{\mathrm{r,i}}}{m_\mathrm{p}}, \\ R_\mathrm{p} &= \sum_i \frac{m_{\mathrm{r},i} R_{\mathrm{r,i}}}{m_\mathrm{p}}, \end{aligned} \tag{S5}$$

which coincides with the Lorentz–Lorenz mixing rule. This can be seen by

$$R^*_\mathrm{p} = \sum_i a_i R^*_{\mathrm{r},i}, \tag{S6}$$

where a_i is the respective mole fraction.

TABLE S1: Values of amino acid residues (AAR) employed for calculating the PSV θ and RI increment for Wiener approximation and volume additivity argument α_r^W and α_r^B, respectively. See text for further details. Values obtained from §[5, 6], †[3] (consensus average), ‡[7].

AAR	$M_r^\S$ in g/mol	$V_r^\dagger$ in 10^{-3}nm^3	θ_r in ml/g	$R_r^{*\ddagger}$ in cm^3	α_r^W in ml/g	α_r^B in ml/g
Arg	156.19	188 ± 10	0.73 ± 0.04	39.47 ± 0.10	0.194 ± 0.016	0.202 ± 0.018
His	137.14	156 ± 6	0.686 ± 0.027	34.62 ± 0.15	0.210 ± 0.012	0.221 ± 0.014
Lys	128.17	173 ± 6	0.811 ± 0.028	34.10 ± 0.20	0.184 ± 0.012	0.191 ± 0.013
Asp	115.09	115.4 ± 2.2	0.604 ± 0.012	26.06	0.193 ± 0.005	0.204 ± 0.006
Glu	129.12	141 ± 5	0.657 ± 0.025	30.07 ± 0.15	0.183 ± 0.011	0.192 ± 0.012
Ser	87.08	91.7 ± 1.8	0.634 ± 0.012	19.16 ± 0.10	0.168 ± 0.006	0.175 ± 0.006
Thr	101.10	118.3 ± 2.3	0.705 ± 0.014	23.82 ± 0.10	0.169 ± 0.006	0.175 ± 0.007
Asn	114.10	120 ± 4	0.634 ± 0.022	26.09 ± 0.20	0.185 ± 0.010	0.194 ± 0.011
Gln	128.13	145 ± 5	0.682 ± 0.024	30.37 ± 0.20	0.181 ± 0.011	0.189 ± 0.012
2Cys	204.26	211 ± 10	0.621 ± 0.029	48.58	0.209 ± 0.013	0.221 ± 0.016
Gly	57.05	59.9 ± 2.2	0.632 ± 0.023	12.81 ± 0.10	0.177 ± 0.011	0.186 ± 0.012
Pro	97.12	123.2 ± 1.8	0.764 ± 0.011	23.74 ± 0.10	0.161 ± 0.005	0.167 ± 0.005
Ala	71.08	87.8 ± 2.3	0.744 ± 0.019	17.15 ± 0.15	0.164 ± 0.009	0.169 ± 0.010
Ile	113.16	166.1 ± 3.4	0.884 ± 0.018	31.87 ± 0.20	0.184 ± 0.008	0.191 ± 0.009
Leu	113.16	168 ± 4	0.894 ± 0.023	31.59 ± 0.15	0.176 ± 0.010	0.181 ± 0.010
Met	131.19	165.2 ± 1.8	0.758 ± 0.008	34.45 ± 0.05	0.199 ± 0.004	0.208 ± 0.004
Phe	147.18	190 ± 7	0.776 ± 0.030	42.21 ± 0.15	0.240 ± 0.013	0.253 ± 0.016
Trp	186.21	228 ± 4	0.737 ± 0.012	55.24 ± 0.30	0.278 ± 0.007	0.297 ± 0.008
Tyr	163.18	191 ± 8	0.706 ± 0.030	44.34	0.240 ± 0.013	0.255 ± 0.016
Val	99.13	139 ± 4	0.843 ± 0.022	26.73 ± 0.13	0.178 ± 0.009	0.184 ± 0.010

C. Errors with assuming volume additivity

- Errors in the PSV considerations
  - The following effects are stated in [1]
    - conformational protein formation from amino acids: ~ 0.012 ml/g
    - volume changes of proteins in solution:
      - swelling and dissolution: $\sim -5 \times 10^{-2}$ ml/g
      - thermal denaturation: $\sim \pm 10^{-2}$ ml/g
      - Coil-helix transition: $\sim +10^{-2}$ ml/g
      - Aggregation: $\sim +5 \times 10^{-3}$ ml/g
      - Sol-gel transition: $\sim -10^{-4}$ ml/g
- Errors in the RI increment considerations
  - [4] found that the predicted RI increment values using the Wiener relation for different proteins are systematically higher than the experimental values. Further they found a linear relationship between predicted and experimental values. Further they propose a $\pi - \pi$ interaction term correction which slightly improves the agreement. We do not employ this correction here. However, we note that this discrepancy could also be partially resolved by not employing the Wiener equation but rather relying on the Biot equation, as well as a different choice of experimental values of R_r^* for the amino acid residues.

D. Employed amino acid residues values

We omitted the contributions of selenocysteine since we could not obtain data on molar refractivity, mass density nor refractive index. For all other amino acid residues (AAR) the employed values are given in table S1.

II. PARAMETER VALUES

In the following we provide the mean values $\pm$ standard deviations of the following quantities; relative volume fraction x, refractive index n, refraction per gram R in cm^3/g, PSV θ in ml/g and RI increment α in ml/g of the

different macro molecules employed throughout this study in Table S2. The RI and refraction per gram values of the lipids employed in the sections *Lipids and proteins in water* and *Larval zebrafish trunk tissue* were obtained from [8] (predicted data by `ACD/Labs Percepta Platform - PhysChem Module`, at $T = 20\,^{\circ}\mathrm{C}$ and a wavelength of $\lambda = 589\,\mathrm{nm}$), from which we computed the PSV *via* the Lorentz–Lorenz relation (Eq. (10)). The associated standard deviation was then estimated *via* Gaussian propagation of uncertainty. For all the computations presented in this study, we interpret a value that has a uncertainty attached to it as a normal distribution, where the standard deviation is given by said uncertainty. For values that were assumed to be precise, we assume a delta distribtuion.

TABLE S2: Rounded mean values ± standard deviations of relative volume fraction x, refractive index n, refraction per gram R in cm^3/g, PSV θ in ml/g and RI increment α in ml/g of the different macro molecules employed throughout this study. Entries without footnote indicate assumptions or derived values (see main text for further information). Assumed to be precise values are stated without standard deviation.

	$\overline{x} \pm \Delta x$	$\overline{n} \pm \Delta n$	$\overline{R} \pm \Delta R$ in cm^3/g	$\overline{\theta} \pm \Delta\theta$ in ml/g	$\overline{\alpha} \pm \Delta\alpha$ in ml/g
Water		1.334		1/0.997	
Lipids and proteins in water					
Proteins (human proteome)	$(1 - \overline{x}_{\mathrm{lip}}) \pm \Delta x_{\mathrm{lip}}$	1.603 ± 0.007[a]	0.2520 ± 0.0033[a]	0.734 ± 0.012[a]	0.197 ± 0.004[a]
Triolein (neutral lipid)	$\overline{x}_{\mathrm{lip}} \pm \Delta x_{\mathrm{lip}}$	1.477[b]	0.30674 ± 0.00034[b]	1.086 ± 0.0012	0.1552 ± 0.0008
Bovine skim milk					
Proteins	0.33[g]				
Caseins	0.20[c]	1.597 ± 0.006[a]	0.2531 ± 0.0006[a]	0.743 ± 0.007[a]	0.1954 ± 0.0029[a]
α_{S1}-Casein	0.42[c]	1.600[a]	0.253[a]	0.740[a]	0.198[a]
α_{S2}-Casein	0.11[c]	1.602[a]	0.253[a]	0.737[a]	0.199[a]
β-Casein	0.35[c]	1.588[a]	0.254[a]	0.753[a]	0.192[a]
κ-Casein	0.12[c]	1.597[a]	0.252[a]	0.740[a]	0.195[a]
Other proteins	0.08[c]	1.604 ± 0.007[a]	0.2530 ± 0.0030[a]	0.736 ± 0.011[a]	0.198 ± 0.004[a]
Fat	0.01[g]	1.462[c]	0.299	1.09[c]	0.141
Lactose	0.52[g]	1.582	0.187	0.562[c]	0.140[c]
Ash	0.06[g]	1.647	0.196	0.541[c]	0.170[c]
Intralipid solution					
Soybean oil	0.89[g]	1.474[h]	0.31	1.09[h]	0.15
Glycerol	0.07[g]	1.474[h]	0.22	0.79[h]	0.11
Lecithin	0.05[g]	1.459[i]	0.27	0.97[j]	0.12
Larval zebrafish trunk tissue					
Proteins (trunk tissue)	0.780 ± 0.022[d]	1.602 ± 0.005[e]	0.2520 ± 0.0025[e]	0.734 ± 0.009[e]	0.1971 ± 0.0033[e]
Lipids	0.220 ± 0.022[d]	1.473 ± 0.016	0.308 ± 0.06	1.100 ± 0.023	0.153 ± 0.016
Triolein	0.28[f]	1.477[b]	0.30674 ± 0.00034[b]	1.0856 ± 0.0012	0.1552 ± 0.0008
Palmitic acid	0.24[f]	1.454[b]	0.3030 ± 0.0012[b]	1.119 ± 0.004	0.1343 ± 0.0029
Oleic acid	0.20[f]	1.467[b]	0.3084 ± 0.0011[b]	1.111 ± 0.004	0.1478 ± 0.0026
Docosahexaenoic acid	0.15[f]	1.521[b]	0.3224 ± 0.0009[b]	1.0587 ± 0.0030	0.1980 ± 0.0023
Stearic acid	0.12[f]	1.456[b]	0.3058 ± 0.0011[b]	1.125 ± 0.004	0.1373 ± 0.0026

[a] computed from AA sequences obtained from [9]
[b] obtained from [8]
[c] obtained from [10]
[g] obtained from the manufacturer
[h] obtained from [11]
[i] obtained from [12]
[j] obtained from [13]
[d] computed from values obtained from [14]
[e] computed from AA sequences obtained from [15] and [9]
[f] computed from values obtained from [16]

III. REFRACTIVE INDEX MIXING MODEL COMPARISONS

Following [17, 18], assuming volume additivity, we may express the different mixing rules as

$$f_i(n) = (1-\varphi_\mathrm{s}) f_i(n_1) + \varphi_\mathrm{s} f_i(n_\mathrm{s}), \tag{S7}$$

where the different f_i are specific for each mixing rule, φ_s and n_s denotes the solute volume fraction and RI, respectively. The f_i under study are listed in table S3.

By solving Eqs. (S7) of the respective mixing rule for the mixture RI, we obtain a functional relationship $n(n_\mathrm{s}, c_\mathrm{s}, \rho_\mathrm{s})$ that can be used to fit experimental data, as shown in Fig. 4A. Since we obtained the solute MD ρ_s from measurements of the mixture MD in dependence of c_s (see Fig. 4B), by fitting the data with $n(n_\mathrm{s}, c_\mathrm{s}, \rho_\mathrm{s})$, we obtain a value of solute RI n_s for each model. We then evaluate the difference between the respective fitted solute RIs and the -based on the biochemical composition- predicted solute RI of the samples under study, using different mixing rules. The results are given in Table S3.

TABLE S3: f_i as defined in Eq. (S7) for different mixing rules and difference between fitted and predicted solute RIs $n_\mathrm{s}^\mathrm{fit} - n_\mathrm{s}^\mathrm{pred}$ for the respective RI mixing rules. The uncertainty intervals were computed employing Gaussian propagation of uncertainty. The predicted RI distributions were obtain as outlined in the main text for $N_0 = 10^3$ and $N_\mathrm{v} = 10^3$. RI Mixture rules were adapted from [17].

		$n_\mathrm{s}^\mathrm{fit} - n_\mathrm{s}^\mathrm{pred}$	
Mixing rule	$f(n)$	Skim milk	20 % intralipid emulsion
Biot	n	-0.005 ± 0.006	-0.0014 ± 0.0023
Series	$1/n$	-0.066 ± 0.008	-0.0141 ± 0.0025
Lichtenecker	$\log n$	-0.032 ± 0.007	-0.0074 ± 0.0024
Lorentz–Lorenz	$\left(n^2-1\right) / \left(n^2+2\right)$	-0.032 ± 0.007	-0.0072 ± 0.0024
Wiener	$\left(n^2-n_1^2\right) / \left(n^2+2n_1^2\right)$	-0.016 ± 0.006	-0.0038 ± 0.0023

IV. DERIVATION OF THE MASS DENSITY–REFRACTIVE INDEX RELATION FOR THE LORENTZ-LORENZ MIXING RULE

Following [18], assuming volume additivity, the Lorentz–Lorenz mixing rule of RIs can be expressed as

$$f_\mathrm{LL}(n) = (1-\varphi_2)\, f_\mathrm{LL}(n_1) + \varphi_2 f_\mathrm{LL}(n_2), \tag{S8}$$

with $f_\mathrm{LL}(n_i) \equiv \left(n_i^2 - 1\right) / \left(n_i^2 + 2\right)$. Next, we solve above equation for n_LL to obtain

$$n_\mathrm{LL} = \left[\frac{2c_2\left(n_2^2 - n_1^2\right) + n_1^2\left(n_2^2+2\right)\rho_2}{c_2\left(n_1-n_2\right)\left(n_1+n_2\right) + \left(n_2^2+2\right)\rho_2}\right]^{-1/2}, \tag{S9}$$

from which we compute the RI increment $\alpha_\mathrm{LL} \equiv \partial n_\mathrm{LL}/\partial c_2$ as

$$\alpha_\mathrm{LL} = \frac{\left(n_1^2+2\right)\left(n_2^2+2\right)\left(n_2^2-n_1^2\right)\rho_2}{2\left[c_2\left(n_1^2-n_2^2\right)+\rho_2\left(n_2^2+2\right)\right]^{3/2}\sqrt{n_1^2\rho_2\left(n_2^2+2\right) - 2c_2\left(n_1^2-n_2^2\right)}}. \tag{S10}$$

Now, Eq.(S10) has to be solved for the solute concentration c_2. While this is analytically possible, the resulting expression is quite lengthy, so we refer to the resulting expression as $c_2\left(\alpha_\mathrm{LL}, n_1, n_2, \theta\right)$. Of course, this solving step can be done numerically, given a set of parameter values. Finally, following the rational of the main text, we insert the resulting expression for the solute concentration in Eq. (6) to obtain

$$\rho = \rho_1(1 - c_2\left(\alpha_\mathrm{LL}, n_1, n_2, \theta\right)\theta) + c_2\left(\alpha_\mathrm{LL}, n_1, n_2, \theta\right). \tag{S11}$$

We note that all other considerations presented in the main text can be adapted for Eq. (S11), while the results will be, unfortunately, more lengthy and difficult to handle.

V. PDF OF A TRUNCATED NORMAL DISTRIBUTION $\mathcal{T}$

For a normal distribution with mean μ and standard deviation σ, the corresponding truncated distribution is denoted by $\mathcal{T}(\mu, \sigma)$. The probability density function (PDF) of $\mathcal{T}$ is defined as

$$t(y) \equiv \begin{cases} \frac{f(y)}{F(1)-F(0)} & 0 \leq y \leq 1 \\ 0 & \text{else} \end{cases}, \tag{S12}$$

where $f(y)$ and $F(y)$ denote the PDF and cumulative distribution function (CDF), respectively, of said normal distribution $\mathcal{N}(\mu, \sigma)$.

VI. MEAN AND STANDARD DEVIATION OF NORMAL MIXTURE DISTRIBUTIONS

Let's consider a one dimensional mixture distribution of two Normal distributions,

$$\mathcal{P}(\mu_1, \mu_2, \sigma_1, \sigma_2; x) = w\mathcal{N}(\mu_1, \sigma_1; x) + (1-w)\mathcal{N}(\mu_2, \sigma_2; x), \tag{S13}$$

with $0 \leq w \leq 1$. The mean and standard deviation of this mixture distribtion is given by

$$\begin{aligned} \mu' &= w\mu_1 + (1-w)\mu_2, \\ \sigma' &= \sqrt{\sigma_2^2 + w\left(\mu_1^2 - 2\mu_1\mu_2 + \mu_2^2 + \sigma_1^2 - \sigma_2^2\right) - w^2\left(\mu_1^2 - \mu_2^2\right)^2}, \end{aligned} \tag{S14}$$

respectively. For the special case of $\sigma_1 = \sigma_2 = \sigma$ we obtain for the standard deviation of the mixture distribution

$$\sigma' = \sqrt{\sigma^2 - \left((w-1)\,w\left(\mu_1 - \mu_2\right)^2\right)} > \sigma, \tag{S15}$$

for all $\mu_1 \neq \mu_2$ and $0 \leq w \leq 1$, which is maximized for $w = 0.5$.

VII. EFFECTIVE PARAMETERS

From the mixing rule of the solute density

$$\rho_\mathrm{s} = \sum_{i=1}^{N_\mathrm{s}} x_{i+1}\rho_{i+1}, \tag{S16}$$

we obtain that the effective PSV is

$$\theta_\mathrm{eff} = 1/\rho_\mathrm{s} = \left(\sum_{i=1}^{N_\mathrm{s}} \frac{x_{i+1}}{\theta_{i+1}}\right)^{-1} = \sum_{i=1}^{N_\mathrm{s}} y_{i+1}\theta_{i+1} = \sum_j \frac{N_j m_j}{m_\mathrm{s}}\theta_j, \tag{S17}$$

where N_s is the nuber of solute voxelinos per voxel and j denotes the different types of solute molecules. The effective RI increment for the Biot equation is then

$$\begin{aligned} \alpha_\mathrm{eff} = \frac{n - n_1}{c} &= \frac{n_1\left(1 - c\sum_{i=1}^{N_\mathrm{s}} y_{i+1}\theta_{i+1}\right) + c\sum_{i=1}^{N_\mathrm{s}} y_{i+1}\theta_{i+1}n_{i+1} - n_1}{c} \\ &= n_1\sum_{i=1}^{N_\mathrm{s}} y_{i+1}\theta_{i+1} - \sum_{i=1}^{N_\mathrm{s}} n_{i+1}y_{i+1}\theta_{i+1} \\ &= \sum_{i=1}^{N_\mathrm{s}} (n_1 - n_{i+1})y_{i+1}\theta_{i+1} \\ &= \sum_{i=1}^{N_\mathrm{s}} y_{i+1}\alpha_{i+1} = \sum_j \frac{N_j m_j}{m_\mathrm{s}}\alpha_j = \theta_\mathrm{eff}\left(n_\mathrm{s} - n_1\right). \end{aligned} \tag{S18}$$

VIII. UNCERTAINTIES OF THE EFFECTIVE PARAMETERS FOR LIPIDS AND PROTEINS IN WATER

The uncertainties of the effective RI increment and the PSV, introduced in Eq. (21) can be computed as follows. The terms $\Delta\alpha^0_{\text{eff}}$ and $\Delta\theta^0_{\text{eff}}$ refer to the standard deviations of the mixture distribution, as given in Eq. (S14).

The uncertainties associated of the effective PSV to deviations in the relative lipid volume fraction Δx_{lip}, can be evaluated by employing Eq. (16), from which we obtain that

$$\frac{\partial\overline{\theta}_{\text{eff}}}{\partial\overline{x}_{\text{lip}}} = \frac{\overline{\theta}_{\text{p}}\overline{\theta}_{\text{lip}}\left(\overline{\theta}_{\text{lip}} - \overline{\theta}_{\text{p}}\right)}{\left(\left(1 - \overline{x}_{\text{lip}}\right)\overline{\theta}_{\text{lip}} + \overline{x}_{\text{lip}}\overline{\theta}_{\text{p}}\right)^2}. \tag{S19}$$

We may write the effective RI increment, using Eq. (16), as

$$\overline{\alpha}_{\text{eff}} = \overline{\theta}_{\text{eff}}\left(\overline{n}_{\text{s}} - n_1\right) = \overline{\theta}_{\text{eff}}\left(\left(1 - \overline{x}_{\text{lip}}\right)\overline{n}_{\text{p}} + \overline{x}_{\text{lip}}\overline{n}_{\text{lip}} - n_1\right). \tag{S20}$$

Hence, we have

$$\frac{\partial\overline{\alpha}_{\text{eff}}}{\partial\overline{x}_{\text{lip}}} = \overline{\theta}_{\text{eff}}\left(\overline{n}_{\text{lip}} - \overline{n}_{\text{p}}\right) + \frac{\partial\overline{\theta}_{\text{eff}}}{\partial\overline{x}_{\text{lip}}}\frac{\overline{\alpha}_{\text{eff}}}{\overline{\theta}_{\text{eff}}}. \tag{S21}$$

IX. REFRACTIVE INDEX VALUES OF LARVAL ZEBRAFISH TRUNK TISSUE AT 96 PF FROM [15]

The RI values of larval zebrafish trunk tissue at 96 hpf employed in this study are not explicitly stated in [15], hence we list them here.

TABLE S4: Refractive indices n and according standard deviations Δn of the trunk tissue of $N = 20$ zebrafish larvae at 96 hpf from [15].

N	1	2	3	4	5	6	7	8	9	10
n	1.36501	1.36524	1.36691	1.36679	1.36556	1.36251	1.36319	1.36098	1.36112	1.36342
Δn	0.00225	0.00369	0.00307	0.00315	0.00268	0.00151	0.00122	0.00210	0.00241	0.00229
N	11	12	13	14	15	16	17	18	19	20
n	1.36763	1.36604	1.36673	1.36670	1.36550	1.36640	1.36633	1.36632	1.36662	1.36693
Δn	0.00182	0.00205	0.00233	0.00211	0.00219	0.00238	0.00220	0.00172	0.00130	0.00238

X. ESTIMATIONS OF x_{lip} AND φ_1 OF LARVAL ZEBRAFISH AT 96 HPF

Based on the measurements of [14], where they determined the wet mass m_{tot}, dry mass m_{dry}, protein mass m_{p} and the lipid mass m_{lip} of larval zebrafish at 96 hpf, we define the relative lipid mass fraction $y_{\text{lip}} \equiv m_{\text{lip}}/(m_{\text{lip}} + m_{\text{p}})$ and consider the ratio

$$\frac{y_{\text{lip}}}{y_{\text{p}}} = \frac{m_{\text{lip}}}{m_{\text{p}}} = \frac{\theta^{\text{eff}}_{\text{p}} x_{\text{lip}}}{\theta^{\text{eff}}_{\text{lip}}(1 - x_{\text{lip}})}, \tag{S22}$$

from which we obtain

$$x_{\text{lip}} = \frac{m_{\text{lip}}\theta^{\text{eff}}_{\text{lip}}}{m_{\text{lip}}\theta^{\text{eff}}_{\text{lip}} + m_{\text{p}}\theta^{\text{eff}}_{\text{p}}}, \tag{S23}$$

and analogously

$$\varphi_1 = \frac{(m_{\text{tot}} - m_{\text{dry}})}{m_{\text{tot}} + m_{\text{dry}}\left(\rho_1\theta^{\text{eff}}_{\text{dry}} - 1\right)}. \tag{S24}$$

Based on this dry mass composition we estimate the distributions of $\theta^{\text{eff}}_{\text{p}}$ and $\theta^{\text{eff}}_{\text{lip}}$ by running the MC simulation of the extended mixture model with $x_{\text{lip}} = 0$ and $x_{\text{lip}} = 1$, respectively. We may now obtain the distribution of x_{lip} by a MC sampling approach, considering the distributions of all individual parameters. Next, we compute $\theta^{\text{eff}}_{\text{dry}}$ by repeating the procedure described above for x_{lip} following the distribution determined previously. With that we obtain the distribution of φ_1.

XI. RELATIVE UNCERTAINTY OF THE RI FOR DIFFERENT MATERIAL PROPERTIES

The dependence of the relative RI deviation on the deviation of the relative lipid volume fraction Δx_{lip} for different deviations of the water volume fraction $\Delta\varphi_1$, as pointed out in the main text, can be qualitatively understood with Fig. S1.

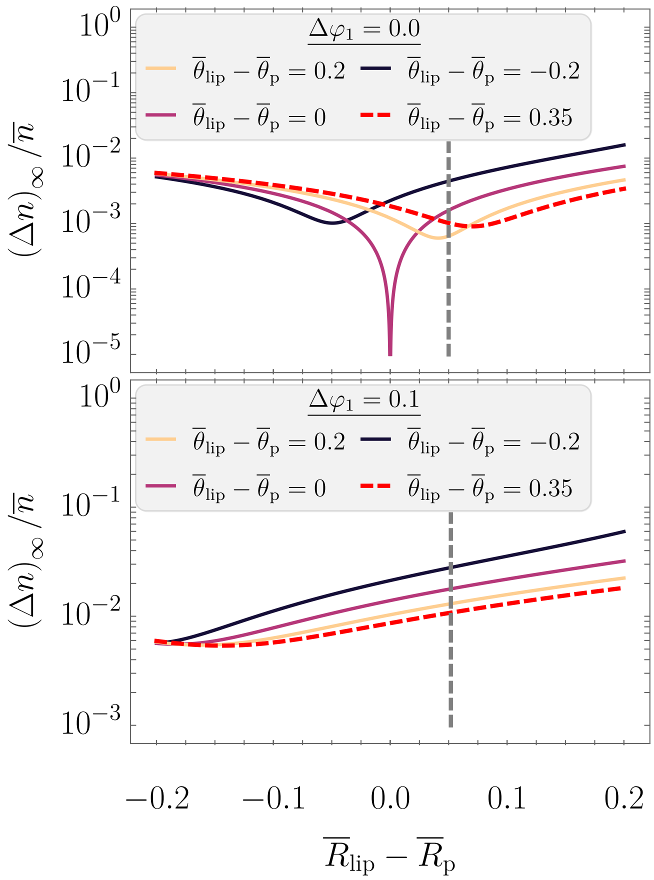

FIG. S1: Asymptotic relative deviation of the RI $(\Delta n)_\infty/\overline{n}$ for different potential differences of lipid and protein PSVs $\overline{\theta}_{\mathrm{lip}} - \overline{\theta}_{\mathrm{p}}$ in dependence of the difference of lipid and protein refractions per gram $\overline{R}_{\mathrm{lip}} - \overline{R}_{\mathrm{p}}$ for the cases of a non-fluctuating water volume fraction $\Delta\varphi_1 = 0$ (top) and a fluctuating water volume fraction $\Delta\varphi_1 = 0.1$ (bottom).

XII. STRATEGIES FOR ESTIMATING THE MD FOR CERTAIN EXPERIMENTAL PARADIGMS

In Fig. S2, we outline possible strategies for estimating the MD, given certain experimental insights. In the following, we use the same abbreviations as in the main text, namely,

- RI = refractive index,
- (S)RS = (stimulated) Raman spectroscopy,
- MS = mass spectrometetry.

We want to point out that the estimation process is heavily dependent on identifying relevant solute constituents of the sample, as well as their PSVs, RIs and/or refractions per gram. For the latter, secondary data bases, such as `ChemSpider` [8], are invaluable. Furthermore, although RI measurements are not necessary to predict $\rho(\delta n)$, as outlined in the main text, comparing predicted and measured RI distributions gives necessary insight on the validity of the prediction.

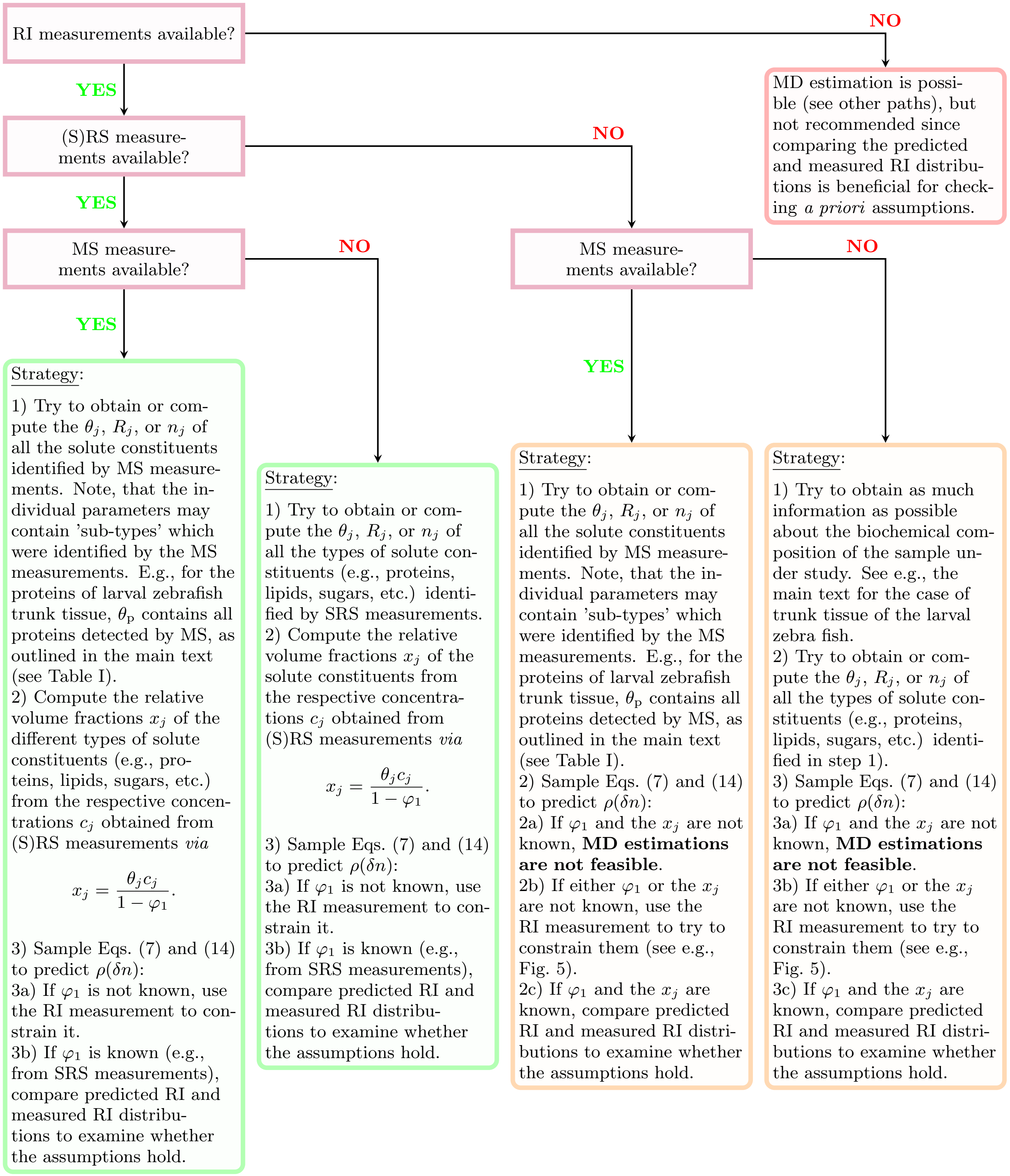

FIG. S2: Flowchart of mass density estimation approaches outlined in this study, based on different experimental paradigms. The abbreviations and symbols are as defined in the main text.

SUPPORTING REFERENCES

[1] A. Zamyatnin, Progress in Biophysics and Molecular Biology **24**, 107 (1972).
[2] H. Zhao, P. H. Brown, and P. Schuck, Biophysical Journal **100**, 2309 (2011).
[3] S. J. PERKINS, European Journal of Biochemistry **157**, 169 (1986).
[4] D. Khago, J. C. Bierma, K. W. Roskamp, N. Kozlyuk, and R. W. Martin, Journal of Physics: Condensed Matter **30**, 435101 (2018).
[5] Wolfram Research, Inc., Wolfram|Alpha Knowledgebase, Champaign, IL (2022). (2022).
[6] S. Kim, J. Chen, T. Cheng, A. Gindulyte, J. He, S. He, Q. Li, B. A. Shoemaker, P. A. Thiessen, B. Yu, L. Zaslavsky, J. Zhang, and E. E. Bolton, Nucleic Acids Research **51**, D1373 (2022).
[7] T. L. McMeekin, M. Wilensky, and M. L. Groves, Biochemical and Biophysical Research Communications **7**, 151 (1962).
[8] ChemSpider

- Triolein, CSID:4593733, http://www.chemspider.com/Chemical-Structure.4593733.html
- Palmitic acid , CSID:960, http://www.chemspider.com/Chemical-Structure.960.html
- Oleic acid, CSID:393217, http://www.chemspider.com/Chemical-Structure.393217.html
- Docosahexaenoic acid, CSID:393183, http://www.chemspider.com/Chemical-Structure.393183.html
- Stearic acid, CSID:5091, http://www.chemspider.com/Chemical-Structure.5091.html

(Accessed Feb 2, 2023).
[9] T. U. Consortium, Nucleic Acids Research **51**, D523 (2022).
[10] P. Walstra, J. T. M. Wouters, and T. J. Geurts, *Dairy Science and Technology* (CRC Press, 2005).
[11] Merck

- Soybean oil, 47122, https://www.sigmaaldrich.com/DE/de/product/supelco/47122
- Glycerol, 104057, https://www.merckmillipore.com/DE/de/product/Glycerol,MDA_CHEM-104057?ReferrerURL=https%3A%2F%2Fwww.google.com%2F

(Accessed Aug 31, 2023).
[12] N. Bansal, T. Truong, and B. Bhandari, Dairy Science & Technology **96**, 861 (2017).
[13] Oxford Lab Fine Chem LLP

- Soybean lecithin, https://www.oxfordlabchem.com/msds/(S-08441)SOYA%20LECITHIN%2030%20Extra%20Pure.pdf

(Accessed Aug 31, 2023).
[14] Y. Long, L. Li, Q. Li, X. He, and Z. Cui, PLOS ONE **7**, 1 (2012).
[15] J. Kolb, V. Tsata, N. John, K. Kim, C. Möckel, G. Rosso, V. Kurbel, A. Parmar, G. Sharma, K. Karandasheva, S. Abuhattum, O. Lyraki, T. Beck, P. Müller, R. Schlüßler, R. Frischknecht, A. Wehner, N. Krombholz, B. Steigenberger, D. Beis, A. Takeoka, I. Blümcke, S. Möllmert, K. Singh, J. Guck, K. Kobow, and D. Wehner, Nature Communications **14**, 6814 (2023).
[16] N. Hachicho, S. Reithel, A. Miltner, H. J. Heipieper, E. Küster, and T. Luckenbach, PLOS ONE **10**, 1 (2015).
[17] F. Pretorius, W. W. Focke, R. Androsch, and E. du Toit, Journal of Molecular Liquids **332**, 115893 (2021).
[18] P. Brocos, A. Piñeiro, R. Bravo, and A. Amigo, Phys. Chem. Chem. Phys. **5**, 550 (2003).
